# Supplementary material for: Differential stability and dynamics of DNA-based and RNA-based coacervates affect non-enzymatic RNA chemistry
Source: Nat Commun. 2025 Oct 21;16:9296. doi: 10.1038/s41467-025-64335-9 (PMC12540774; doi:10.1038/s41467-025-64335-9)
Supplement: Supplementary file 1 — Supplementary Information [file 41467_2025_64335_MOESM1_ESM.docx]

Differential stability and dynamics of DNA- and RNA-based coacervates affect non-enzymatic RNA chemistry

Karina K. Nakashima,^1,2,†^ Fatma Zohra Mihoubi,^1,2,†^ Jagandeep S. Saraya,^3^ Kieran O. Russell,^4^ Fidan Rahmatova,^1,2^ James D. Robinson,^3^ Maria Julia Maristany,^4,5^ Jan Huertas,^4,6^ Roger Rubio-Sánchez,^7^ Rosana Collepardo-Guevara,^4,5,6*^ Derek K. O’Flaherty,^3*^ and Claudia Bonfio^1,2,*^

^1^Department of Biochemistry, University of Cambridge, CB2 1GA Cambridge, UK

^2^Institut de Science et d’Ingénierie Supramoléculaires, CNRS UMR 7006, University of Strasbourg, 67000 Strasbourg, France

^3^Department of Chemistry, University of Guelph, ON N1G 2W1, Guelph, Canada

^4^Yusuf Hamied Department of Chemistry, University of Cambridge, CB2 1EW Cambridge, UK

^5^Cavendish Laboratory, Department of Physics, University of Cambridge, CB3 0HE, Cambridge, UK

^6^Department of Genetics, University of Cambridge, CB2 3EH, Cambridge, UK

^7^Department of Chemical Engineering and Biotechnology, University of Cambridge, CB3 0AS Cambridge, UK

^†^These authors contributed equally

^*^corresponding authors: [rc597@cam.ac.uk](mailto:rc597@cam.ac.uk), [doflaher@uoguelph.ca](mailto:doflaher@uoguelph.ca), [cb2036@cam.ac.uk](mailto:cb2036@cam.ac.uk)

Supplementary Information

Contents

[Supplementary Tables 2](#_Toc206681364)

[Supplementary Figures 27](#_Toc206681365)

# Supplementary Tables

**Table S1.** Peptide sequences used in the present work. All sequences are written *N*- to *C*-termini, are not protected (*i.e.*, H-peptide-OH) and used as TFA salts (SIH = Synthesised in-house).

| **Peptide sequence** | **Net charge** | **Charge density** | **Source** |
| --- | --- | --- | --- |
| **Dimers** |  |  |  |
| R_2_ | 2 | 1 | SIH |
| **Trimers** |  |  |  |
| R_3_ | 3 | 1 | GenScript |
| **Tetramers** |  |  |  |
| R_4_ | 4 | 1 | SIH, GenScript |
| **Pentamers** |  |  |  |
| R_5_ | 5 | 1 | GenScript |
| **Hexamers** |  |  |  |
| R_6_ | 6 | 1 | GenScript |
| **Other** **lengths** |  |  |  |
| R_7_ | 7 | 1 | GenScript |
| R_8_ | 8 | 1 | GenScript |
| R_9_ | 9 | 1 | GenScript |
| R_10_ | 10 | 1 | GenScript |
| E_10_ | -10 | 1 | GenScript |

**Table S2.** Oligonucleotide acronyms and sequences used in the present work (SIH = synthesised in-house).

| **Name** | **Sequence** | **Source** |
| --- | --- | --- |
| dA_3_ | 5′-AAA-3′ | SIH |
| DNA_5_ | 5′-ACTGA-3′ | IDT |
| DNA_6_ | 5′-ACTGAC-3′ | IDT |
| DNA_7_ | 5′-ACTGACT-3′ | IDT |
| DNA_8_ | 5′-ACTGACTG-3′ | IDT |
| DNA_10_ | 5′-ACTGACTGAC-3′ | IDT |
| DNA_12_ | 5′-ACTGACTGACTG -3′ | IDT |
| DNA_16_ | 5′-ACTGACTGACTGACTG-3′ | IDT |
| DNA_20_ | 5′-ACTGACTGACTGACTGACTG-3′ | IDT |
| DNA_40_ | 5′-ACTGACTGACTGACTGACTGACTGACTGACTGACTGACTG-3′ | IDT |
| dA_10_ | 5′-AAAAAAAAAA-3′ | IDT |
| dT_10_ | 5′-TTTTTTTTTT-3′ | IDT |
| dC_10_ | 5′-CCCCCCCCCC-3′ | IDT |
| dG_10_ | 5′-GGGGAGGGGA-3′ | IDT |
| Cy3-5nt | 5′-TCAGT-Cy_3_-3′ | IDT |
| Cy3-8nt | 5′-Cy3-TGACTGAC-3′ | IDT |
| Cy3-16nt | 5′-Cy3-TGACTGACTGACTGAC-3′ | IDT |
| Cy3-32nt | 5′-Cy3-TGACTGACTGACTGACTGAC-3′ | IDT |
| Cy3-dA_11_ | 5′-Cy3-AAAAAAAAAA-3′ | IDT |
| DNA_8_* ^a^ | 5′-CAGTCAGT-3′ | IDT |
| HNA_8_ | 5′-ArCrUGArCrUG-3′ | IDT |
| RNA_8_ | 5′-r(ACUGACUG)-3′ | IDT, SIH |
| HNA_12_ | 5′-ArCrUGArCrUGArCrUG-3′ | IDT |
| RNA_12_ | 5′-r(ACUGACUGACUG)-3′ | IDT, SIH |
| RNA_20_ | 5′- r(ACUGACUGACUGACUGACUG)-3′ | IDT, SIH |
| dA_11_ | 5′-AAAAAAAAAAA-3′ | Eurofins |
| dA_21_ | 5′-AAAAAAAAAAAAAAAAAAAAA-3′ | Eurofins |
| dA_31_ | 5′-AAAAAAAAAAAAAAAAAAAAAAAAAAAAAA-3′ | Eurofins |
| dA_41_ | 5′-AAAAAAAAAAAAAAAAAAAAAAAAAAAAAAAAAAAAAAAAA-3′ | Eurofins |
| dA_51_ | 5′-AAAAAAAAAAAAAAAAAAAAAAAAAAAAAAAAAAAAAAAAAAAAAAAAAAA-3′ | Eurofins |
| dA_12_ | 5′-AAAAAAAAAAAA-3′ | SIH |
| dA_16_ | 5′-AAAAAAAAAAAAAAAA-3′ | SIH |
| dT_16_ | 5′-TTTTTTTTTTTTTTTT-3′ | SIH |
| rA_12_ | 5′-r(AAAAAAAAAAAA)-3′ | SIH |
| rA_16_ | 5′-r(AAAAAAAAAAAAAAAA)-3′ | SIH |
| Arich_12_ | 5′-AAGTAAAGTAAA-3′ | SIH |
| Template^b^ | 5′-r(GGGUCGAGCG)-3′ | SIH |
| FAM-RNA_8_ (primer)^b^ | 5′-FAM-r(CGCUCGAC)-3′ | SIH |
| Template^c^ | 5′-r(GGCAGUCAGU)-3′ | SIH |
| FAM-RNA_8_ (primer)^c^ | 5′-FAM-r(ACUGACUG)-3′ | SIH |
| FAM-DNA_8_ | 5′-FAM-ACTGACTG-3′ | SIH |
| DNA_11_-Phos | 5′-ACTGACTGACT-Phos-3′ | IDT |
| Phos-DNA_10_-Phos | 5′-Phos-CTGACTGACT-Phos-3′ | IDT |
| Broccoli aptamer A | 5′-r(GCGGAGACGGUCGGGUCCAGAUA)-3′ | Eurofins |
| Broccoli aptamer B | 5′-r(UAUCUGUCGAGUAGAGUGUGGGCUCCGC)-3′ | Eurofins |

^a^ DNA_8_* is the complementary sequence to DNA_8_

^b^ primer/template system referred to as non-complementary to the host oligonucleotide

^c^ primer/template system referred to as complementary to the host oligonucleotide

**Table S3.** Critical salt concentrations (CSCs) measured for mixtures comprising 20 mM amino acid of Arg homopeptides and 5 mM nucleotide (unless otherwise stated) in 25 mM HEPES pH 7.4 and room temperature.

| **Peptide** | **Oligonucleotide** | **CSC (mM, NaCl)** |
| --- | --- | --- |
| R_3_ | DNA_8_ | 0 |
| R_3_ | HNA_8_ | 59.7 |
| R_3_ | RNA_8_ | 54.2 |
| R_3_ | DNA_12_ | 38.8 ± 4.7 |
| R_3_ | HNA_12_ | 52.7 |
| R_3_ | RNA_12_ | 205.2 |
| R_3_ | DNA_12_:RNA_12_ 1:1 | 44.7 |
| R_3_ | DNA_20_ | 85.0 |
| R_4_ | DNA_8_ | 99.3 ± 4.7 |
| R_4_ | HNA_8_ | 113.7 |
| R_4_ | RNA_8_ | 215.9 |
| R_4_ | DNA_12_ | 196.4 |
| R_4_ | HNA_12_ | 237.8 |
| R_4_ | RNA_12_ | 379.2 |
| R_4_ | DNA_12_:RNA_12_ 1:1 | 258.5 |
| R_4_ | DNA_16_ | 212.6 |
| R_4_ | DNA_20_ | 204.5 ± 23.3 |
| R_4_ | RNA_20_ | 430.7 |
| R_4_ | DNA_40_ | 278.1 |
| R_4_ | polyU | 492.9 |
| R_5_ | DNA_20_ | 348.1 |
| R_6_ | DNA_6_ | 143.6 |
| R_6_ | DNA_8_ | 313.7 |
| R_6_ | HNA_8_ | 222.1 |
| R_6_ | RNA_8_ | 210.7 |
| R_6_ | dA_10_ | 99.9 |
| R_6_ | dA_11_ | 123.4 |

| **Peptide** | **Oligonucleotide** | **CSC (mM, NaCl)** |
| --- | --- | --- |
| R_6_ | dA_12_^a^ | 280.1 |
| R_6_ | rA_12_^a^ | 508.4 |
| R_6_ | DNA_12_^a^ | 409.2 |
| R_6_ | DNA_12_ | 415.9 ± 6.1 |
| R_6_ | HNA_12_ | 431.9 |
| R_6_ | RNA_12_ | 693.8 |
| R_6_ | DNA_12_:RNA_12_ 1:1 | 422.4 |
| R_6_ | Arich_12_^a^ | 261.7 |
| R_6_ | dA_15_ | 153.5 |
| R_6_ | dA_16_^a^ | 296.4 |
| R_6_ | DNA_16_ | 475.1 |
| R_6_ | DNA_20_ | 473.8 |
| R_6_ | dA_31_ | 243.5 |
| R_6_ | DNA_40_ | 500.0 |
| R_6_ | dA_41_ | 256.6 |
| R_6_ | dA_51_ | 269.8 |
| R_7_ | DNA_7_ | 369.3 |
| R_8_ | DNA_8_ | 503 ± 33.7 |
| R_8_ | HNA_8_ | 368.6 |
| R_8_ | RNA_8_ | 402.6 |
| R_8_ | DNA_12_ | 601.8 ± 45.3 |
| R_8_ | DNA_16_ | 743.5 |
| R_8_ | DNA_20_ | 684.2 ± 55.4 |
| R_10_ | dA_10_ | 258.2 |
| R_10_ | dT_10_ | 279.0 |
| R_10_ | DNA_10_ | 583.8 ± 37.1 |
| R_10_ | DNA_12_ | 634.2 |

^a^ [nt] = 10 mM

**Table S4.** Critical salt concentrations (CSCs) measured for peptide/peptide and peptide/oligonucleotide coacervates at different component concentrations (phase diagram studies). Concentrations refer to amino acid (aa) or nucleotide (nt) concentrations.

| **Mixture** | **[aa] (mM)** | **[nt] (mM)** | **CSC (mM)** |
| --- | --- | --- | --- |
| **R_4_/DNA_8_** | 2 | 5 | 0 |
|  | 3 | 5 | 22.3 |
|  | 5 | 5 | 49.4 |
|  | 10 | 5 | 67.3 |
|  | 20 | 5 | 99.3 ± 4.7 |
|  | 40 | 5 | 110.0 |
|  | 20 | 1 | 0 |
|  | 20 | 2 | 51.8 |
|  | 20 | 5 | 99.3 ± 4.7 |
|  | 20 | 10 | 122.7 |
|  | 20 | 15 | 118.6 |
|  | 20 | 20 | 140.1 |
| **R_3_/DNA_12_** | 5 | 5 | 0 |
|  | 10 | 5 | 0 |
|  | 15 | 5 | 25.0 |
|  | 20 | 5 | 38.8 ± 4.7 |
|  | 30 | 5 | 36.7 |
|  | 40 | 5 | 20.7 |
|  | 60 | 5 | 47.0 |
|  | 20 | 2.5 | 30.7 |
|  | 20 | 5 | 38.8 ± 4.7 |
|  | 20 | 10 | 46.4 |
|  | 20 | 15 | 37.8 |
|  | 20 | 20 | 23.2 |

| **Mixture** | **[aa] (mM)** | **[nt] (mM)** | **CSC (mM)** |
| --- | --- | --- | --- |
| **R_4_/DNA_20_** | 3 | 5 | 93.0 |
|  | 5 | 5 | 123.5 |
|  | 10 | 5 | 175.9 |
|  | 15 | 5 | 213.8 |
|  | 20 | 5 | 201.6 ± 19.3 |
|  | 30 | 5 | 270.3 |
| **R_8_/DNA_20_** | 3 | 5 | 391.5 |
|  | 5 | 5 | 503.8 |
|  | 10 | 5 | 503.0 |
|  | 15 | 5 | 609.0 |
|  | 20 | 5 | 684.2 ± 55.4 |
|  | 30 | 5 | 674.8 |
| **R_10_/E_10_**^a^ | 5 | 10 | 0 |
|  | 8 | 10 | 90.6 ± 5.6 |
|  | 10 | 10 | 95.2 ± 0.4 |
|  | 12 | 10 | 103.8 ± 22.9 |
|  | 20 | 10 | 0 |
|  | 10 | 5 | 0 |
|  | 10 | 8 | 81.9 ± 12.9 |
|  | 10 | 12 | 107.5 ± 6.1 |
|  | 10 | 15 | 93.7 ± 5.7 |
|  | 10 | 20 | 84.8 |
|  | 10 | 40 | 0 |

^a^ Concentrations reported are [Arg] and [Glu]

**Table S5.** Amino acid concentrations required for the coacervation of dipeptides with oligonucleotides of different lengths. *N/A* stands for ‘non-applicable’.

| **Peptide dimer** | **Oligonucleotide** | **Phase** | **Amino acid concentration required** |
| --- | --- | --- | --- |
| **R_2_** | DNA_8_ | Soluble | *N/A* |
|  | DNA_12_ | Soluble | *N/A* |
|  | DNA_20_ | Soluble | *N/A* |
|  | RNA_8_ | Soluble | *N/A* |
|  | RNA_12_ | **Droplets** | 60 mM |
|  | RNA_20_ | **Droplets** | 40 mM |

**Table S6.** Parameters calculated from the linear fit (CSC = a(1/N) + b) for the CSCs of peptides and oligonucleotides when one (or both) lengths are varied.

| **Peptide** | **Oligonucleotide** | **a** | **b** | **R^2^** | **Calculated N_min_ (-a/b)** | **Empirical N_min_** |
| --- | --- | --- | --- | --- | --- | --- |
| R_6_ | DNA_N_ | -2107.8 | 564.4 | 0.81 | 3.7 | 5 |
| R_6_ | polyA_N_ | -2105.7 | 308.3 | 0.99 | 6.8 | 11 |
| R_N_ | DNA_12_ | -2561.6 | 866.9 | 0.99 | 2.9 | 3 |
| R_N_ | DNA_20_ | -2951.5 | 1007.6 | 0.90 | 2.9 | 3 |
| R_N_ | DNA_8_ | -2588.7 | 742.7 | 0.96 | 3.5 | 4 |
| R_N_ | HNA_8_ | -1610.3 | 564.4 | 0.89 | 3.0 | 3 |
| R_N_ | RNA_8_ | -1653.8 | 616.9 | 0.99 | 2.7 | 3 |
| R_N_ | RNA_12_ | -2835.8 | 1104.2 | 0.96 | 2.6 | 3 |
| R_N_ | HNA_12_ | -2275.5 | 809.7 | 0.99 | 2.8 | 3 |
| R_N_ | RNA_12_:DNA_12_ 1:1 | -2266.0 | 808.4 | 0.99 | 2.8 | 3 |

**Table S7.** Number of contacts per oligonucleotide strand with arginine residues, as computed with atomistic simulations. Results are categorised by mode of interaction for a given peptide/nucleic acid combination. Each value in a repeat is an average value for all oligonucleotide chains of that peptide/oligonucleotide mixture over time.

| **Number**  **of contacts** | **Mixture** | | | |
| --- | --- | --- | --- | --- |
|  | **R_3_/DNA_8_** | **R_3_/RNA_8_** | **R_4_/DNA_8_** | **R_4_/RNA_8_** |
|  | **H-bonding** | | | |
| **Repeat 1** | 8.32 | 10.30 | 9.35 | 11.06 |
| **Repeat 2** | 7.28 | 9.06 | 10.48 | 10.94 |
| **Repeat 3** | 8.82 | 11.17 | 8.71 | 11.81 |
| **Repeat 4** | 8.15 | 9.38 | 9.16 | 11.68 |
| **Repeat 5** | 7.56 | 10.58 | 9.69 | 11.42 |
| **Average** | **8.02** | **10.1** | **9.48** | **11.4** |
| **Standard deviation** | 0.55 | 0.8 | 0.59 | 0.3 |
|  | **Ionic** | | | |
| **Repeat 1** | 3.19 | 3.51 | 4.28 | 3.95 |
| **Repeat 2** | 3.38 | 4.37 | 4.30 | 4.24 |
| **Repeat 3** | 4.04 | 4.18 | 3.70 | 4.98 |
| **Repeat 4** | 3.99 | 4.28 | 4.09 | 4.71 |
| **Repeat 5** | 3.05 | 3.52 | 4.06 | 4.57 |
| **Average** | **3.53** | **3.97** | **4.09** | **4.49** |
| **Standard deviation** | 0.41 | 0.38 | 0.22 | 0.36 |
|  | **Stacking** | | | |
| **Repeat 1** | 0.68 | 1.59 | 0.71 | 1.70 |
| **Repeat 2** | 0.72 | 1.30 | 0.77 | 1.48 |
| **Repeat 3** | 0.95 | 1.40 | 0.92 | 0.88 |
| **Repeat 4** | 0.53 | 1.34 | 0.97 | 2.01 |
| **Repeat 5** | 0.74 | 1.59 | 0.83 | 2.03 |
| **Average** | **0.726** | **1.44** | **0.840** | **1.62** |
| **Standard deviation** | 0.135 | 0.13 | 0.097 | 0.42 |

**Table S8.** Number of contacts per oligonucleotide strand with arginine residues, as computed with atomistic simulations. Results are categorised by mode of interaction per nucleobase for a given peptide/nucleic acid combination. Each value in a repeat is an average value for all oligonucleotide chains of that peptide/oligonucleotide mixture over time.

| **Number**  **of contacts** | **R_3_/DNA_8_** | | | |
| --- | --- | --- | --- | --- |
|  | **A** | **C** | **G** | **T** |
|  | **H-bonding** | | | |
| **Repeat 1** | 1.29 | 2.68 | 2.27 | 2.08 |
| **Repeat 2** | 1.12 | 2.75 | 1.64 | 1.78 |
| **Repeat 3** | 0.99 | 2.54 | 3.05 | 2.24 |
| **Repeat 4** | 1.16 | 2.90 | 2.21 | 1.88 |
| **Repeat 5** | 1.08 | 2.62 | 1.90 | 1.96 |
| **Average** | **1.13** | **2.70** | **2.22** | **1.99** |
| **SEM** | 0.10 | 0.12 | 0.48 | 0.16 |
|  | **Ionic** | | | |
| **Repeat 1** | 0.33 | 0.94 | 0.86 | 1.06 |
| **Repeat 2** | 0.62 | 1.00 | 0.77 | 0.98 |
| **Repeat 3** | 0.73 | 0.93 | 1.17 | 1.20 |
| **Repeat 4** | 0.63 | 0.83 | 1.25 | 1.28 |
| **Repeat 5** | 0.37 | 0.79 | 0.89 | 1.00 |
| **Average** | **0.54** | **0.90** | **0.99** | **1.10** |
| **SEM** | 0.16 | 0.08 | 0.19 | 0.12 |
|  | **Stacking** | | | |
| **Repeat 1** | 0.33 | 0.12 | 0.08 | 0.15 |
| **Repeat 2** | 0.32 | 0.18 | 0.15 | 0.07 |
| **Repeat 3** | 0.24 | 0.14 | 0.38 | 0.19 |
| **Repeat 4** | 0.16 | 0.14 | 0.11 | 0.13 |
| **Repeat 5** | 0.27 | 0.14 | 0.19 | 0.14 |
| **Average** | **0.27** | **0.14** | **0.18** | **0.14** |
| **SEM** | 0.06 | 0.02 | 0.11 | 0.04 |

| **Number**  **of contacts** | **R_3_/RNA_8_** | | | |
| --- | --- | --- | --- | --- |
|  | **A** | **C** | **G** | **U** |
|  | **H-bonding** | | | |
| **Repeat 1** | 1.00 | 3.27 | 3.26 | 2.76 |
| **Repeat 2** | 1.30 | 2.61 | 2.79 | 2.36 |
| **Repeat 3** | 1.22 | 3.00 | 3.58 | 3.37 |
| **Repeat 4** | 1.43 | 2.59 | 2.52 | 2.84 |
| **Repeat 5** | 1.26 | 2.81 | 3.70 | 2.81 |
| **Average** | **1.24** | **2.86** | **3.17** | **2.83** |
| **SEM** | 0.14 | 0.25 | 0.45 | 0.32 |
|  | **Ionic** | | | |
| **Repeat 1** | 0.86 | 0.82 | 0.75 | 1.09 |
| **Repeat 2** | 0.74 | 1.24 | 1.12 | 1.27 |
| **Repeat 3** | 0.77 | 0.96 | 1.36 | 1.09 |
| **Repeat 4** | 0.50 | 1.25 | 1.12 | 1.41 |
| **Repeat 5** | 0.58 | 0.87 | 0.92 | 1.15 |
| **Average** | **0.69** | **1.03** | **1.05** | **1.20** |
| **SEM** | 0.13 | 0.18 | 0.21 | 0.12 |
|  | **Stacking** | | | |
| **Repeat 1** | 0.34 | 0.42 | 0.49 | 0.34 |
| **Repeat 2** | 0.34 | 0.38 | 0.37 | 0.20 |
| **Repeat 3** | 0.40 | 0.24 | 0.51 | 0.25 |
| **Repeat 4** | 0.24 | 0.34 | 0.45 | 0.32 |
| **Repeat 5** | 0.44 | 0.29 | 0.55 | 0.31 |
| **Average** | **0.35** | **0.33** | **0.47** | **0.29** |
| **SEM** | 0.07 | 0.06 | 0.06 | 0.05 |

| **Number**  **of contacts** | **R_4_/DNA_8_** | | | |
| --- | --- | --- | --- | --- |
|  | **A** | **C** | **G** | **T** |
|  | **H-bonding** | | | |
| **Repeat 1** | 1.18 | 3.59 | 2.24 | 2.34 |
| **Repeat 2** | 1.77 | 3.41 | 2.92 | 2.39 |
| **Repeat 3** | 1.19 | 2.73 | 2.46 | 2.33 |
| **Repeat 4** | 1.35 | 2.66 | 2.61 | 2.54 |
| **Repeat 5** | 1.08 | 3.26 | 2.75 | 2.60 |
| **Average** | **1.31** | **3.13** | **2.59** | **2.44** |
| **SEM** | 0.24 | 0.37 | 0.23 | 0.11 |
|  | **Ionic** | | | |
| **Repeat 1** | 0.58 | 1.16 | 1.11 | 1.43 |
| **Repeat 2** | 0.58 | 1.24 | 1.21 | 1.28 |
| **Repeat 3** | 0.46 | 0.78 | 1.33 | 1.13 |
| **Repeat 4** | 0.68 | 0.68 | 1.54 | 1.20 |
| **Repeat 5** | 0.52 | 1.02 | 1.25 | 1.26 |
| **Average** | **0.56** | **0.98** | **1.29** | **1.26** |
| **SEM** | 0.07 | 0.22 | 0.14 | 0.10 |
|  | **Stacking** | | | |
| **Repeat 1** | 0.26 | 0.15 | 0.14 | 0.16 |
| **Repeat 2** | 0.26 | 0.16 | 0.25 | 0.11 |
| **Repeat 3** | 0.29 | 0.22 | 0.26 | 0.15 |
| **Repeat 4** | 0.36 | 0.20 | 0.24 | 0.17 |
| **Repeat 5** | 0.30 | 0.20 | 0.24 | 0.09 |
| **Average** | **0.29** | **0.18** | **0.23** | **0.14** |
| **SEM** | 0.04 | 0.03 | 0.04 | 0.03 |

| **Number**  **of contacts** | **R_4_/RNA_8_** | | | |
| --- | --- | --- | --- | --- |
|  | **A** | **C** | **G** | **U** |
|  | **H-bonding** | | | |
| **Repeat 1** | 1.20 | 3.05 | 3.50 | 3.31 |
| **Repeat 2** | 1.61 | 2.41 | 3.78 | 3.14 |
| **Repeat 3** | 1.58 | 3.01 | 4.01 | 3.20 |
| **Repeat 4** | 1.62 | 4.05 | 2.89 | 3.12 |
| **Repeat 5** | 1.53 | 3.50 | 3.58 | 2.81 |
| **Average** | **1.51** | **3.20** | **3.55** | **3.12** |
| **SEM** | 0.16 | 0.55 | 0.38 | 0.17 |
|  | **Ionic** | | | |
| **Repeat 1** | 0.47 | 1.11 | 1.13 | 1.24 |
| **Repeat 2** | 0.62 | 1.18 | 0.97 | 1.47 |
| **Repeat 3** | 0.73 | 1.33 | 1.32 | 1.60 |
| **Repeat 4** | 0.66 | 1.40 | 1.31 | 1.34 |
| **Repeat 5** | 0.63 | 1.12 | 1.37 | 1.45 |
| **Average** | **0.62** | **1.23** | **1.22** | **1.42** |
| **SEM** | 0.09 | 0.12 | 0.15 | 0.12 |
|  | **Stacking** | | | |
| **Repeat 1** | 0.40 | 0.24 | 0.61 | 0.45 |
| **Repeat 2** | 0.26 | 0.32 | 0.57 | 0.33 |
| **Repeat 3** | 0.16 | 0.21 | 0.30 | 0.22 |
| **Repeat 4** | 0.45 | 0.44 | 0.52 | 0.61 |
| **Repeat 5** | 0.33 | 0.41 | 0.67 | 0.62 |
| **Average** | **0.32** | **0.33** | **0.53** | **0.45** |
| **SEM** | 0.10 | 0.09 | 0.13 | 0.16 |

**Table S9.** Number of total contacts per oligonucleotide strand with arginine residues, as computed with atomistic simulations. The number of contacts established with unique arginine residues or unique peptide chains is also specified. Each value in a repeat is an average value for all oligonucleotide chains of that peptide/oligonucleotide mixture over time.

| **Number**  **of contacts** | **Mixture** | | | |
| --- | --- | --- | --- | --- |
|  | **R_3_/DNA_8_** | **R_3_/RNA_8_** | **R_4_/DNA_8_** | **R_4_/RNA_8_** |
|  | **Total contacts** | | | |
| **Repeat 1** | 12.19 | 15.40 | 14.33 | 16.71 |
| **Repeat 2** | 11.37 | 14.72 | 15.55 | 16.66 |
| **Repeat 3** | 13.81 | 16.75 | 13.34 | 17.68 |
| **Repeat 4** | 12.66 | 15.00 | 14.23 | 18.40 |
| **Repeat 5** | 11.35 | 15.70 | 14.58 | 18.01 |
| **Average** | **12.3** | **15.5** | **14.4** | **17.5** |
| **Standard deviation** | 0.9 | 0.7 | 0.7 | 0.7 |
|  | **With unique arginine residues** | | | |
| **Repeat 1** | 6.30 | 7.43 | 7.46 | 8.25 |
| **Repeat 2** | 6.09 | 6.93 | 8.35 | 7.51 |
| **Repeat 3** | 7.36 | 7.61 | 7.38 | 8.61 |
| **Repeat 4** | 6.90 | 7.03 | 7.46 | 8.55 |
| **Repeat 5** | 5.83 | 6.90 | 7.81 | 8.45 |
| **Average** | **6.50** | **7.18** | **7.69** | **8.28** |
| **Standard deviation** | 0.56 | 0.29 | 0.36 | 0.40 |
|  | **With unique peptide chains** | | | |
| **Repeat 1** | 3.88 | 4.42 | 3.99 | 4.01 |
| **Repeat 2** | 3.97 | 4.14 | 3.96 | 3.90 |
| **Repeat 3** | 4.45 | 4.55 | 3.73 | 4.61 |
| **Repeat 4** | 4.35 | 4.28 | 3.54 | 4.40 |
| **Repeat 5** | 3.52 | 4.10 | 3.72 | 4.22 |
| **Average** | **4.03** | **4.30** | **3.79** | **4.23** |
| **Standard deviation** | 0.34 | 0.17 | 0.17 | 0.25 |

**Table S10.** Number of free peptide chains for a given mixture, as computed with atomistic simulations. Each value in a repeat is an average value for all oligonucleotide chains of that peptide/oligonucleotide mixture over time.

| **Number**  **of free peptides** | **Mixture** | | | |
| --- | --- | --- | --- | --- |
|  | **R_3_/DNA_8_** | **R_3_/RNA_8_** | **R_4_/DNA_8_** | **R_4_/RNA_8_** |
| **Repeat 1** | 12.5 | 11.4 | 13.7 | 10.6 |
| **Repeat 2** | 14.2 | 8.9 | 11.2 | 11.1 |
| **Repeat 3** | 11.1 | 11.2 | 12.8 | 12.3 |
| **Repeat 4** | 10.6 | 11.0 | 12.1 | 11.8 |
| **Repeat 5** | 11.2 | 10.7 | 11.7 | 8.8 |
| **Average** | **11.9** | **10.6** | **12.3** | **10.9** |
| **Standard deviation** | 1.3 | 0.9 | 0.9 | 1.2 |

**Table S11.** Partition coefficients calculated from confocal fluorescence microscopy images. K_p_ values were calculated from fluorescence intensities measured using Fiji.

| **Peptide** | **Oligonucleotide** | **Probe** | **I_in_** | **I_out_** | **K_p_** | **N** |
| --- | --- | --- | --- | --- | --- | --- |
| R_4_ | DNA_8_ | FAM-DNA_8_ | 12672.9 | 898.9 | 20.3 ± 4.85 | 201 |
| R_4_ | RNA_8_ | FAM-DNA_8_ | 10117.3 | 1805.5 | 15.3 ± 3.6 | 308 |
| R_4_ | DNA_8_ | FAM-RNA_8_ | 15148.5 | 1767.7 | 21.0 ± 5.5 | 214 |
| R_4_ | RNA_8_ | FAM-RNA_8_ | 11540.0 | 1449.1 | 20.5 ± 3.6 | 363 |
| R_8_ | DNA_8_ | FITC-R_8_ | 40.4 | 2.06 | 19.9 ± 9.4 | 519 |
| R_8_ | RNA_8_ | FITC-R_8_ | 37.0 | 1.5 | 24.9 ± 10.4 | 365 |
| R_4_ | DNA_8_ | Broccoli aptamer | 1917.9 | 125.6 | 15.3 ± 8.3 | 59 |
| R_4_ | RNA_8_ | Broccoli aptamer | 2704.9 | 249.1 | 11.6 ± 3.0 | 366 |
| R_4_ | DNA_16_ | Broccoli aptamer | 2797.3 | 224.4 | 11.1 ±5.8 | 307 |
| R_10_ | E_10_ | Broccoli aptamer | 4825.2 | 175.6 | 30.8 ± 11.3 | 321 |
| R_3_ | DNA_12_ | Cy_3_-A_11_ | 5062.7 | 262.1 | 19.1 ± 5.9 | 120 |
|  |  | Cy_3_-A_31_ | 12193.2 | 517.5 | 23.5 ± 7.8 | 180 |
|  |  | Cy_3_-A_51_ | 12837.7 | 642.3 | 20.1 ± 7.2 | 197 |
| R_4_ | DNA_8_ | Cy_3_-A_11_ | 14126.5 | 547.5 | 25.8 ± 10.3 | 331 |
|  |  | Cy_3_-A_31_ | 18301.1 | 672.2 | 26.8 ± 9.0 | 330 |
|  |  | Cy_3_-A_51_ | 14905.3 | 480.5 | 32.5 ± 11.5 | 324 |
| R_4_ | DNA_8_ | Mg Green, 0 mM Mg^2+^ | 3848.0 | 6651.0 | 0.58 | - |
| R_4_ | RNA_8_ | Mg Green, 0 mM Mg^2+^ | 2756.4 | 4383.3 | 0.63 | - |
| R_10_ | E_10_ | Mg Green, 0 mM Mg^2+^ | 786.1 | 3409.5 | 0.23 | - |
| R_4_ | DNA_8_ | Mg Green, 5 mM Mg^2+^ | 4122.8 | 8821.8 | 0.47 | - |
| R_4_ | RNA_8_ | Mg Green, 5 mM Mg^2+^ | 2617.9 | 6242.7 | 0.42 | - |
| R_10_ | E_10_ | Mg Green, 5 mM Mg^2+^ | 443.3 | 3919.1 | 0.11 | - |
| R_3_ | DNA_12_ | Cy3-8nt | 29.6 | 0.1 | 133.3 ± 54.9 | 133 |
|  |  | Cy3-16nt | 49.3 | 0.4 | 108.3 ± 67.2 | 171 |
|  |  | Cy3-32nt | 47.6 | 1.1 | 52.9 ± 16.9 | 209 |
| R_4_ | DNA_8_ | Cy3-8nt | 72.3 | 1.8 | 38.9 ± 13.3 | 106 |
|  |  | Cy3-16nt | 64.8 | 1.6 | 46.2 ± 17.8 | 131 |
|  |  | Cy3-32nt | 38.8 | 1.7 | 33.9 ±15.1 | 175 |
| R_4_ | DNA_16_ | Cy3-8nt | 32.2 | 1.8 | 19.1 ± 4.6 | 124 |
|  |  | Cy3-16nt | 26.9 | 0.7 | 45.1 ± 19.5 | 89 |
|  |  | Cy3-32nt | 25.4 | 1.8 | 13.4 ± 4.6 | 124 |
| R_8_ | DNA_16_ | Cy3-8nt | 26.4 | 0.4 | 61.4 ± 21.3 | 122 |
|  |  | Cy3-16nt | 23.2 | 2.2 | 11.9 ± 4.7 | 209 |
|  |  | Cy3-32nt | 18.3 | 0.4 | 42.6 ± 27.1 | 135 |
| R_4_ | RNA_8_ | Cy3-8nt | 40.5 | 1.3 | 30.4 ± 10.5 | 98 |
| R_4_ | dsDNA_8_^a^ | Cy3-8nt | 33.4 | 0.7 | 48.5 ± 16.6 | 40 |
| R_10_ | dA_10_ | Cy3-dA_11_ | 58.4 | 0.7 | 83.1 ± 31.1 | 115 |
| R_10_ | dT_10_ | Cy3-dA_11_ | 53.2 | 2.1 | 25.4 ± 8.2 | 172 |
| R_10_ | dC_10_ | Cy3-dA_11_ | 60.1 | 1.6 | 36.9 ± 12.5 | 181 |
| R_10_ | DNA_10_ | Cy3-dA_11_ | 138.1 | 2.4 | 15.9 ± 4.5 | 139 |

^a^ ds denotes double-stranded DNA, prepared with DNA_8_ and DNA_8_* oligonucleotides.

**Table S12.** Parameters obtained from fitting FRAP profiles to the exponential decay: y = y_0_ + A_1_e^(-t/τ)^, where y is the normalised fluorescence and t is the time since photobleaching.

| **Peptide** | **Oligonucleotide** | **Probe** | **y_0_** | **A_1_** | **τ (s)** | **R^2^** |
| --- | --- | --- | --- | --- | --- | --- |
| R_3_ | DNA_12_^a^ | Cy3-8nt | 0.96 | -1.13 | 5.86 | 0.996 |
|  |  | Cy3-16nt | 0.78 | -0.79 | 6.73 | 0.994 |
|  |  | Cy3-32nt | 0.55 | -0.84 | 5.17 | 0.999 |
| R_4_ | DNA_8_^a^ | Cy3-8nt | 0.78 | -1.16 | 10.06 ± 5.02^a^ | 0.999 |
|  |  | Cy3-16nt | 0.68 | -1.08 | 6.64 | 0.999 |
|  |  | Cy3-32nt | 0.60 | -0.85 | 7.83 | 0.999 |
| R_4_ | DNA_8_:RNA_8_ 1:1^a^ | Cy3-8nt | 0.72 | -0.66 | 26.24 | 0.995 |
| R_4_ | RNA_8_^a^ | Cy3-8nt | 0.56 | -0.46 | 61.5 ± 4.7 | 0.998 |
| R_4_ | dsDNA_8_^a^ | Cy3-8nt | 0.96 | -0.92 | 8.9 | 0.999 |
|  |  | Cy3-16nt | 0.55 | -0.35 | 10.6 | 0.980 |
| R_4_ | DNA_12_^b^ | Cy3-8nt | 0.83 | -0.79 | 13.14 | 0.996 |
| R_4_ | DNA_16_^a^ | Cy3-8nt | 0.79 | -0.76 | 18.25 | 0.999 |
|  |  | Cy3-16nt | 0.69 | -0.60 | 26.06 | 0.999 |
|  |  | Cy3-32nt | 0.66 | -0.54 | 25.97 | 0.996 |
| R_6_ | dA_12_^b^ | Cy3-8nt | 0.76 | -0.90 | 4.93 ± 0.89 | 0.864 |
| R_6_ | mA_12_ (dA_12_:rA_12_ 1:1)^b^ | Cy3-8nt | 0.75 | -0.71 | 12.69 | 0.973 |
| R_6_ | dA_3_:rA_12_ 1:1^b^ | Cy3-8nt | 0.70 | -0.77 | 12.46 | 0.861 |
| R_6_ | rA_12_^a^ | Cy3-8nt | 0.63 | -0.53 | 27.77 | 0.945 |
| R_6_ | rA_12_^b^ | Cy3-8nt | 0.44 | -0.30 | 35.79 ± 2.82 | 0.951 |
| R_6_ | rA_12_^b,c^ | Cy3-8nt | 0.61 | -0.49 | 23.49 | 0.937 |
| R_6_ | DNA_12_^b^ | Cy3-8nt | 0.42 | -0.24 | 78.77 | 0.840 |
| R_6_ | Arich_12_^b^ | Cy3-8nt | 0.76 | -0.74 | 14.56 | 0.993 |
| R_6_ | dA_16_ ^b^ | Cy3-8nt | 0.84 | -1.30 | 4.27 | 0.850 |
| R_6_ | rA_16_ ^b^ | Cy3-8nt | 0.46 | -0.37 | 45.81 | 0.988 |
| R_8_ | DNA_16_^a^ | Cy3-8nt | 0.60 | -0.41 | 61.95 | 0.999 |
|  |  | Cy3-16nt | 0.53 | -0.37 | 80.62 | 0.999 |
|  |  | Cy3-32nt | 0.47 | -0.30 | 94.68 | 0.999 |
| R_10_ | dA_10_^a^ | Cy3-dA11 | 0.69 | -1.18 | 3.36 | 0.994 |
| R_10_ | dT_10_^a^ | Cy3-dA11 | 0.64 | -0.53 | 9.70 | 0.995 |
| R_10_ | dC_10_^a^ | Cy3-dA11 | 0.63 | -0.63 | 6.15 | 0.999 |
| R_10_ | DNA_10_^a^ | Cy3-dA11 | 0.72 | -0.60 | 28.5 | 0.998 |

^a^ [Arg] = 20 mM, [nt] = 5 mM

^b^ [Arg] = 20 mM, [nt] = 10 mM

^c^ 50mM [NaCl]

**Table S13.** Representative denaturing polyacrylamide gel image of PE in primitive coacervates. A = w/ peptide, no NaCl (coacervates); B = no peptide, no NaCl; C = w/ peptide, w/ NaCl; D = no peptide, w/ NaCl.

| **Mixture** | **Conditions** | **0h, 1h, 3h, 6h, 24h** |
| --- | --- | --- |
| R_4_/dA_12_  40:5 [Arg]:[nt] | A | 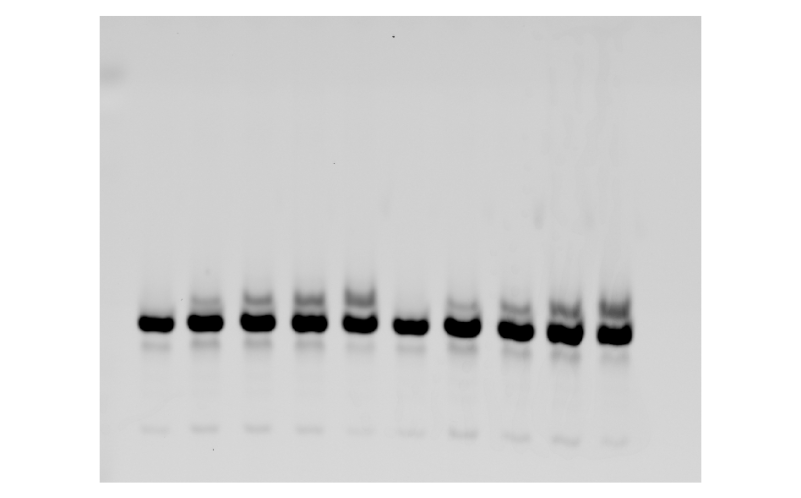 |
|  | B | 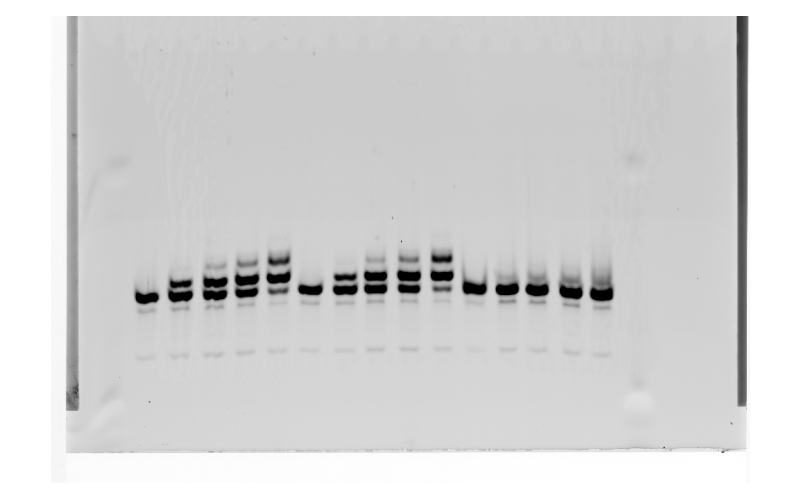 |
|  | C | 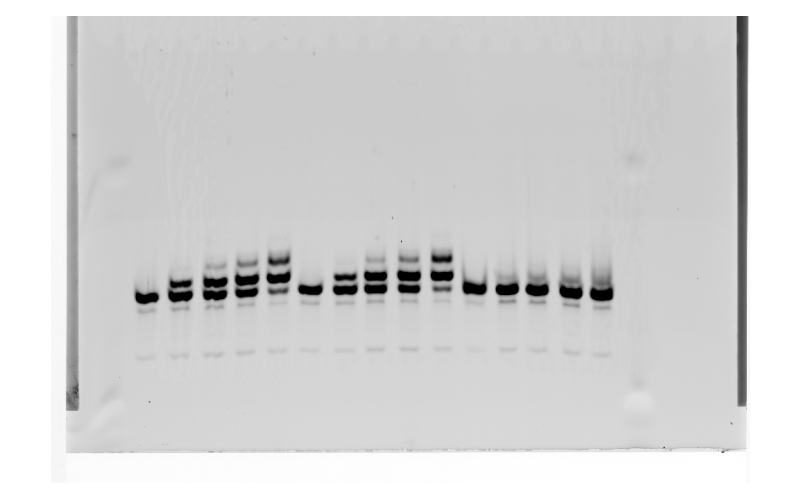 |
|  | D | 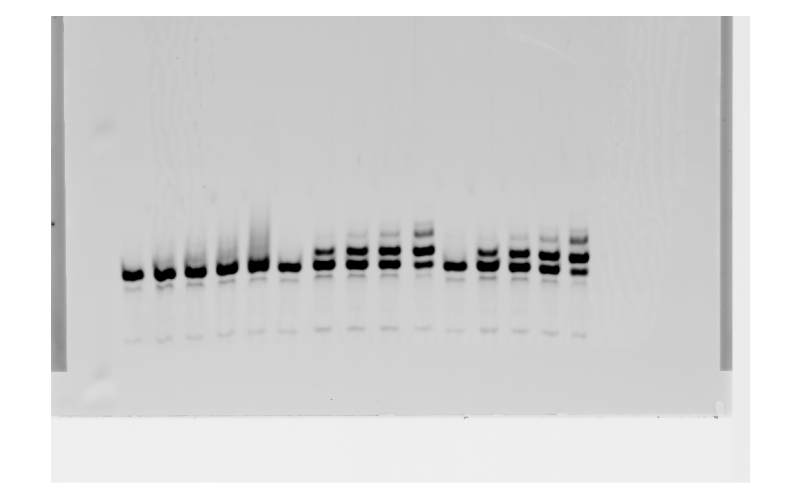 |
| R_6_/dA_12_  20:10 [Arg]:[nt]^a^ | A | 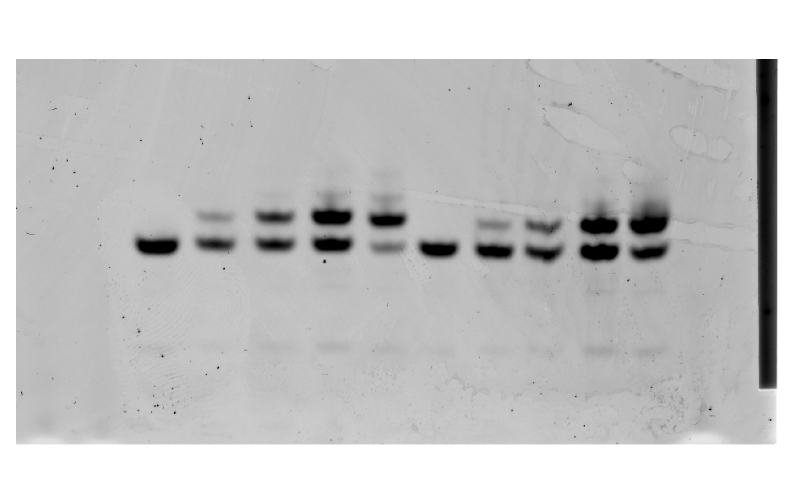 |
|  | B | 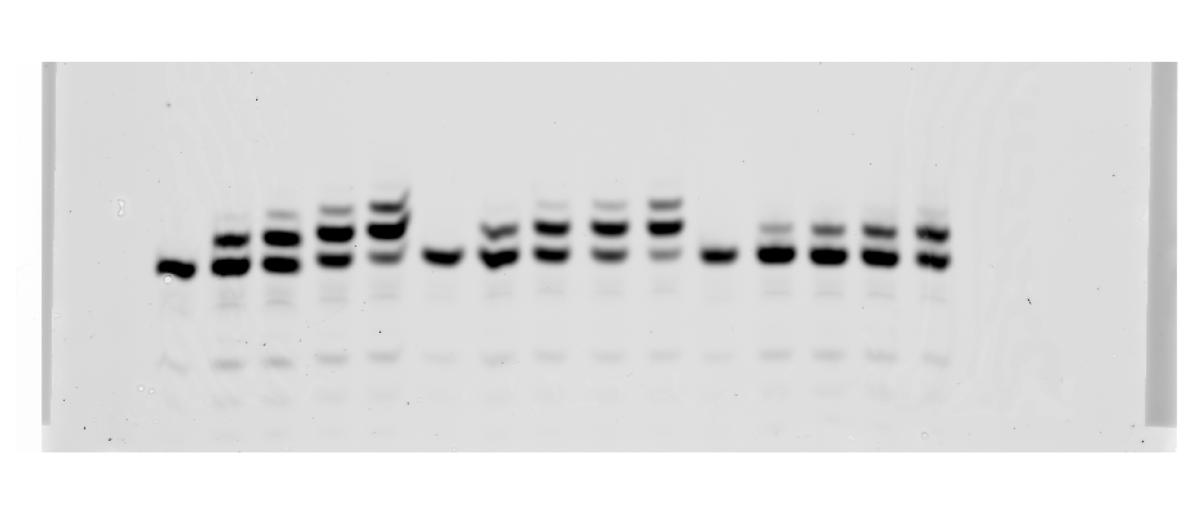 |
|  | C | 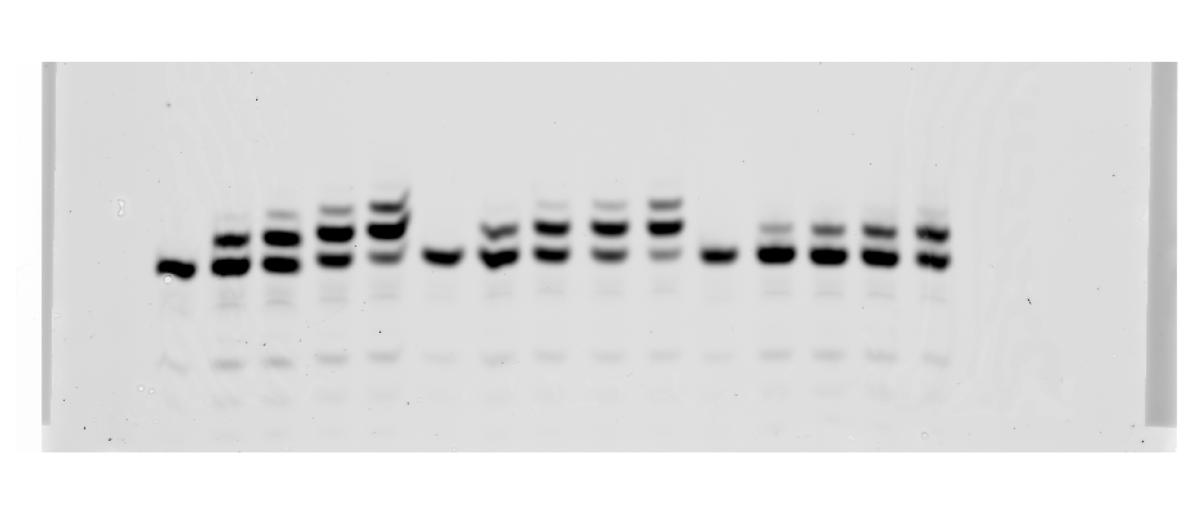 |
|  | D | 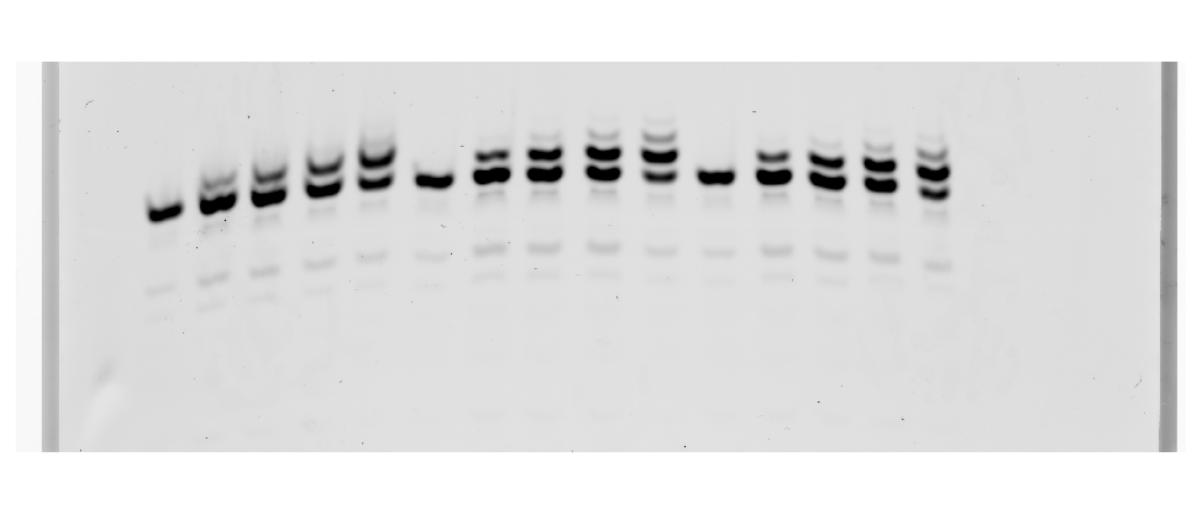 |
| R_6_/dA_12_  20:10 [Arg]:[nt] | A | 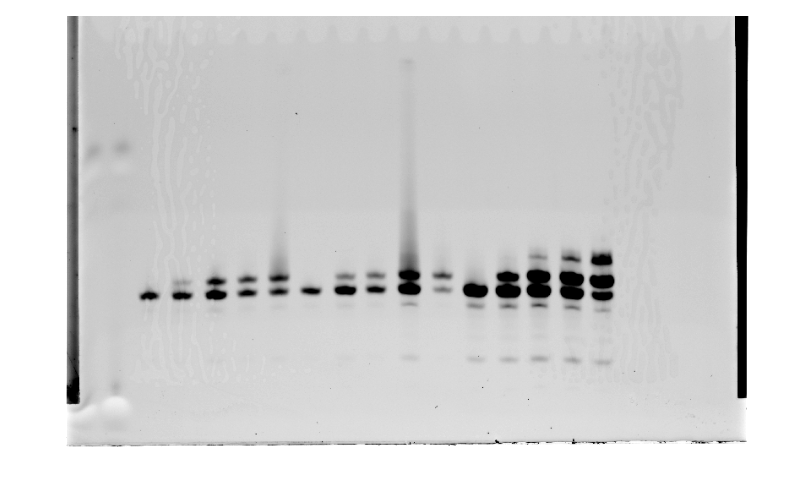 |
|  | B | 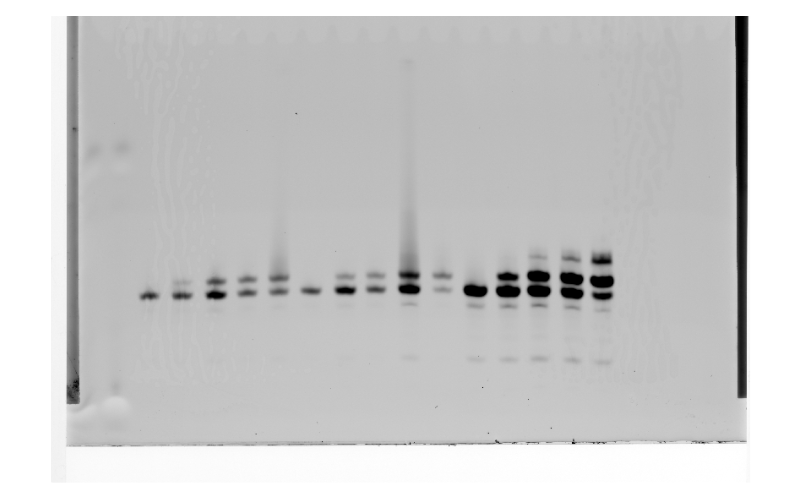 |
|  | C | 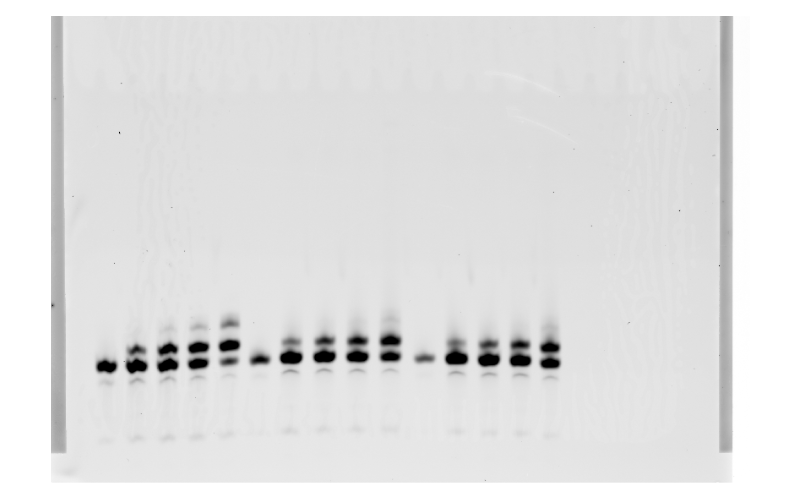 |
|  | D | 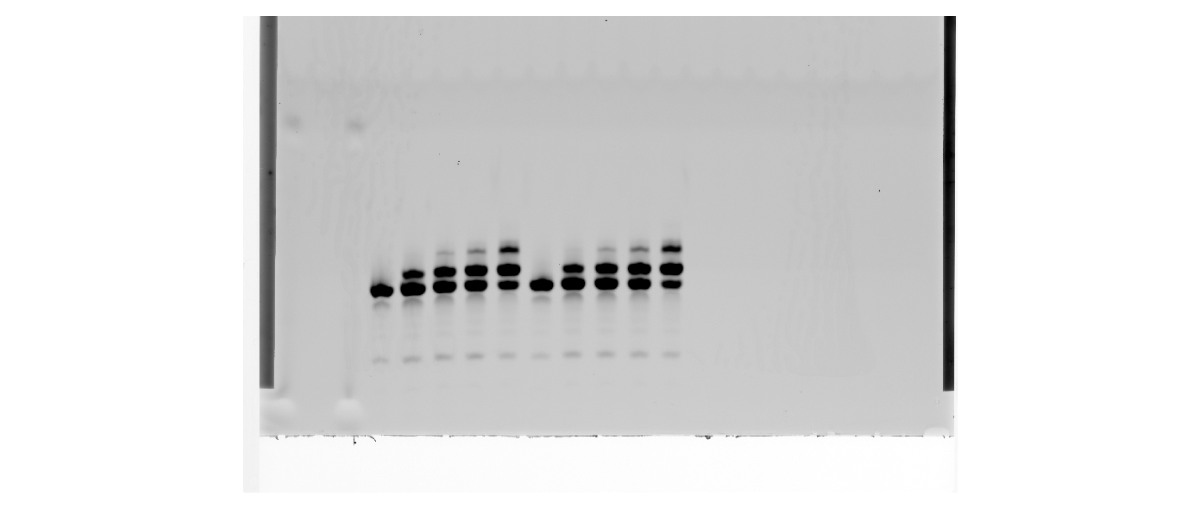 |
| R_6_/rA_12_  20:10 [Arg]:[nt]^a^ | A | 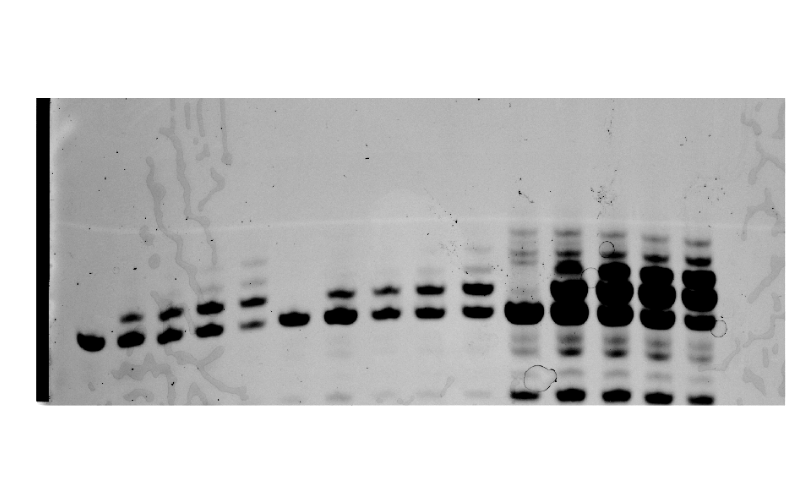 |
|  | A (+ 50 mM NaCl) | 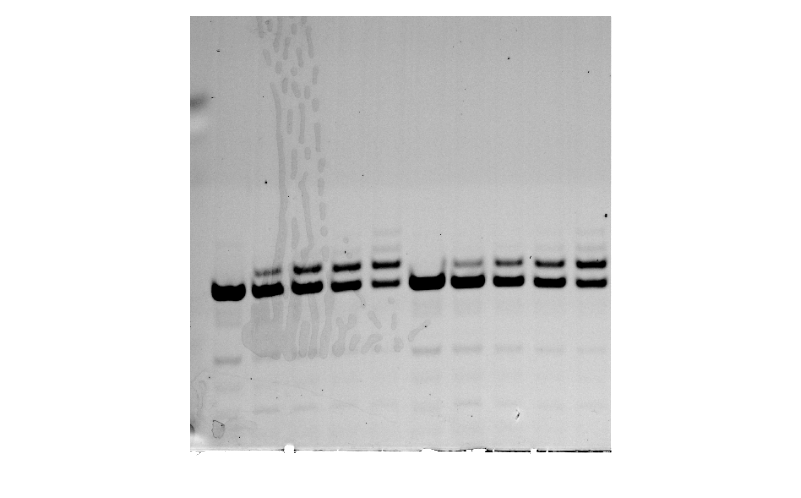 |
|  | B | 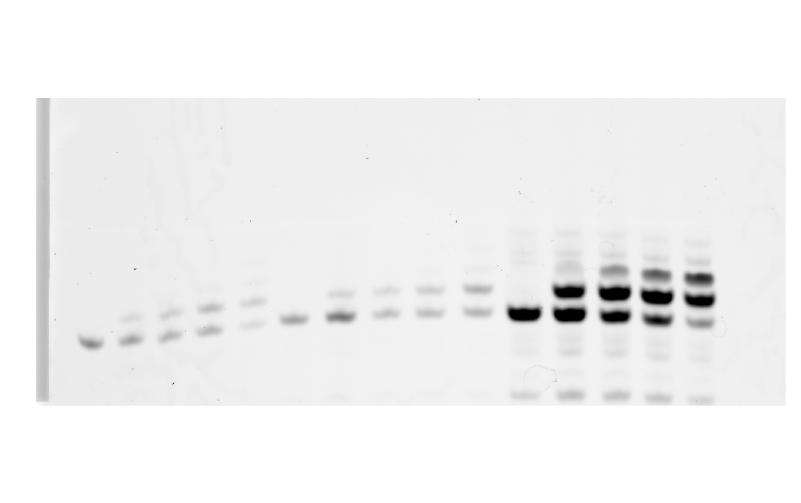 |
|  | C | 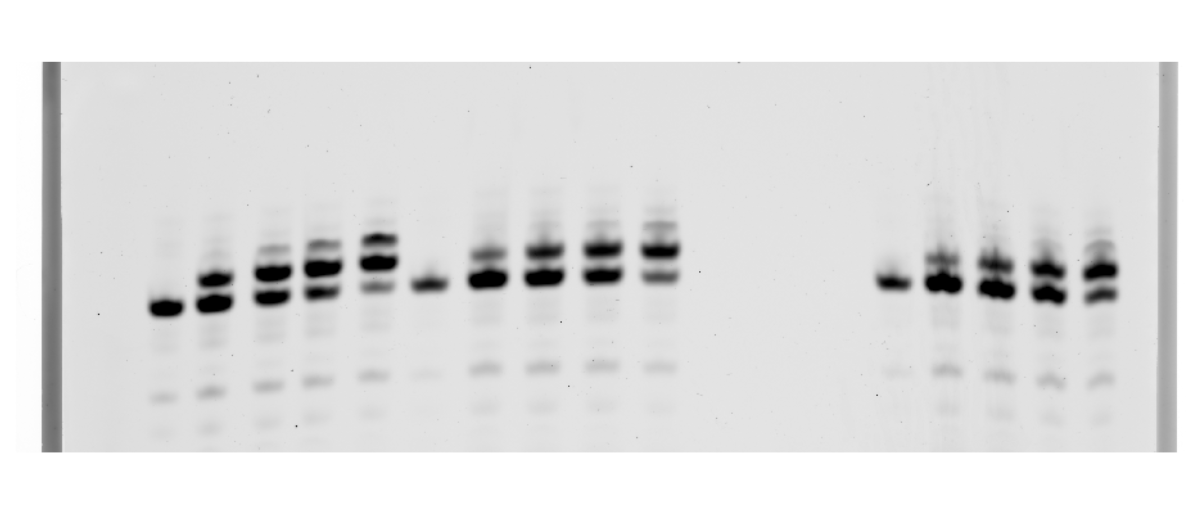 |
|  | D | 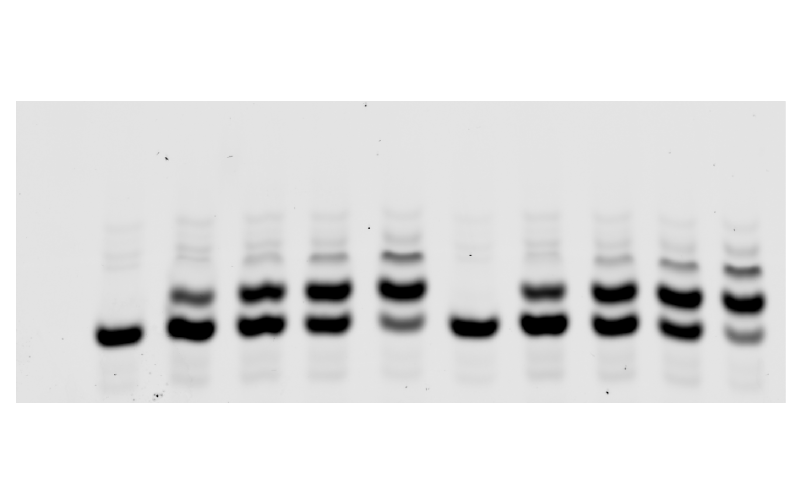 |
| R_6_/mA_12_  20:10 [Arg]:[nt]^a^ | A | 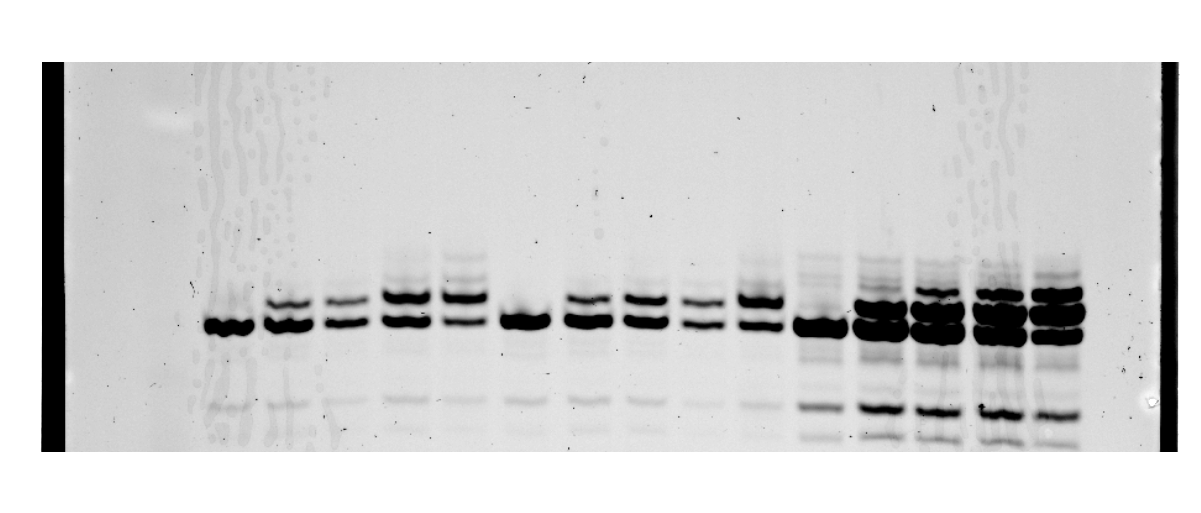 |
|  | B | 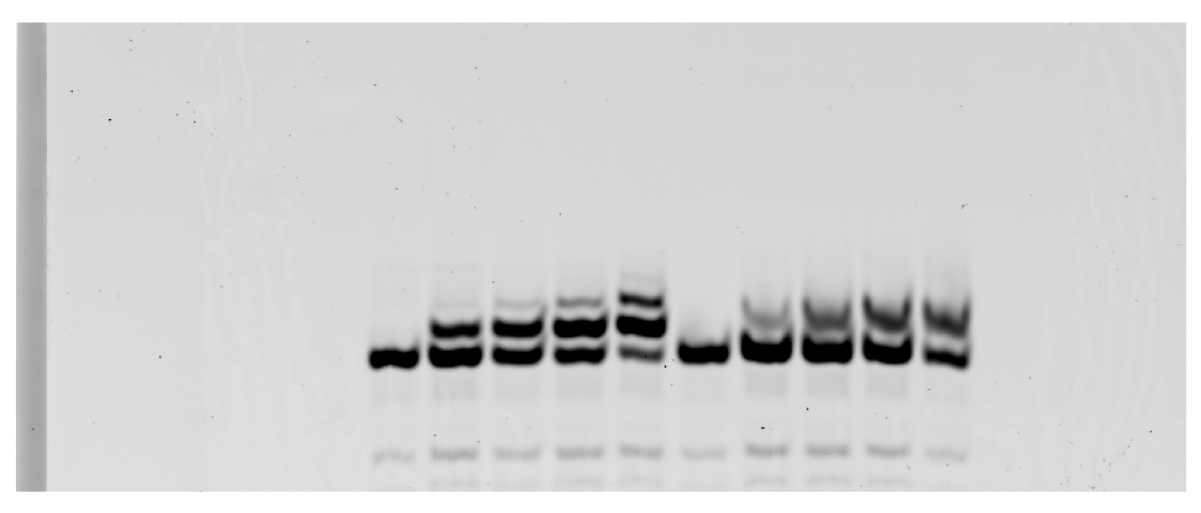 |
|  | C | 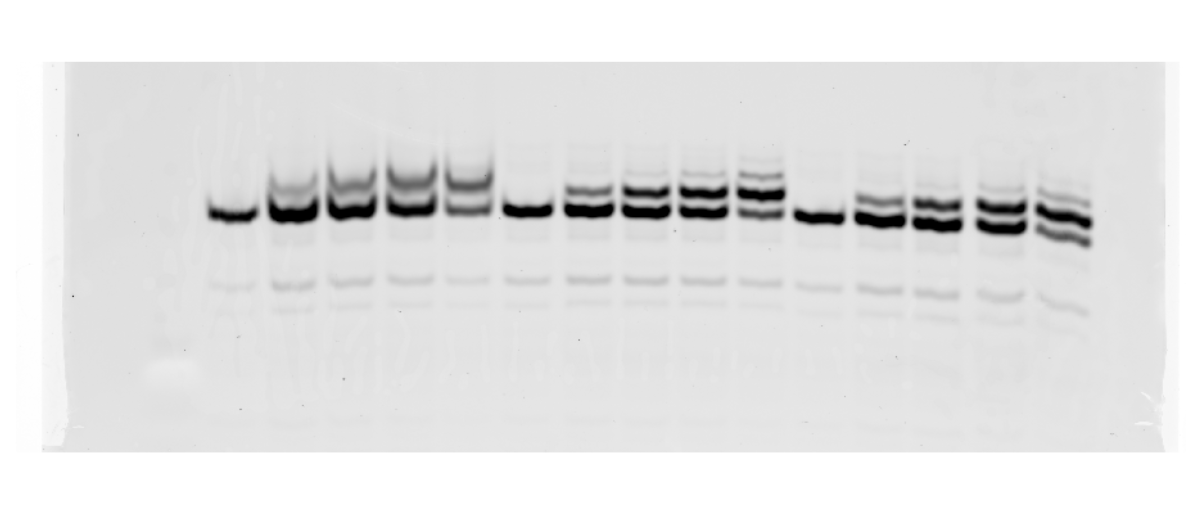 |
|  | D | 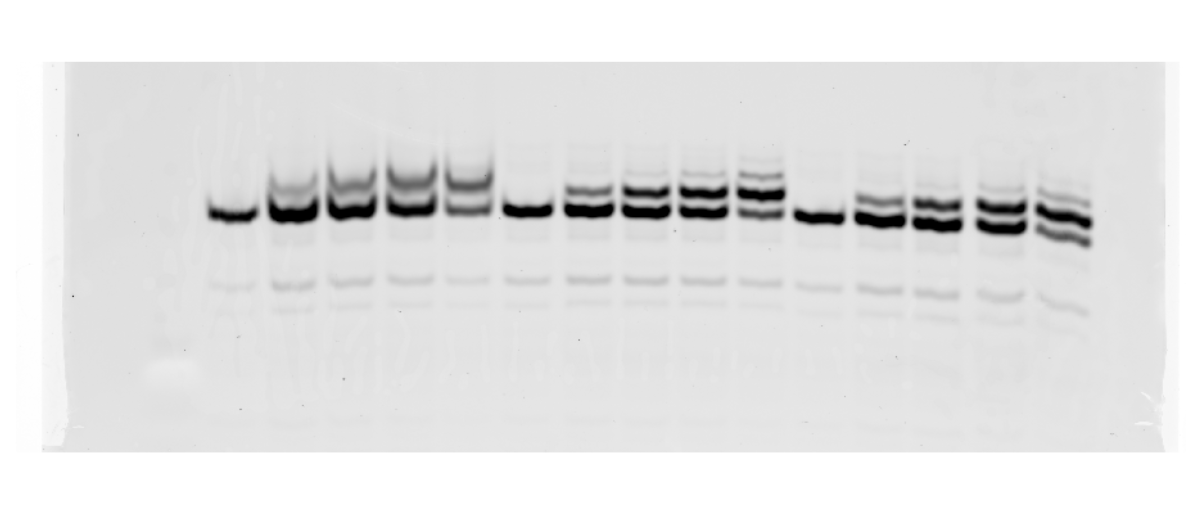 |
| R_6_/dA_12_  40:5 [Arg]:[nt]^a^ | A | 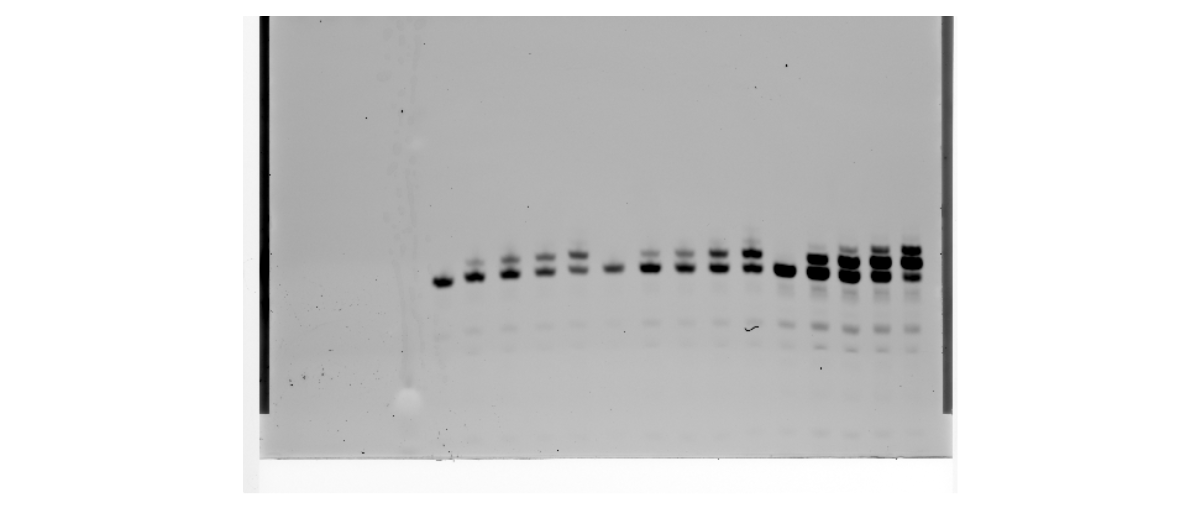 |
|  | B | 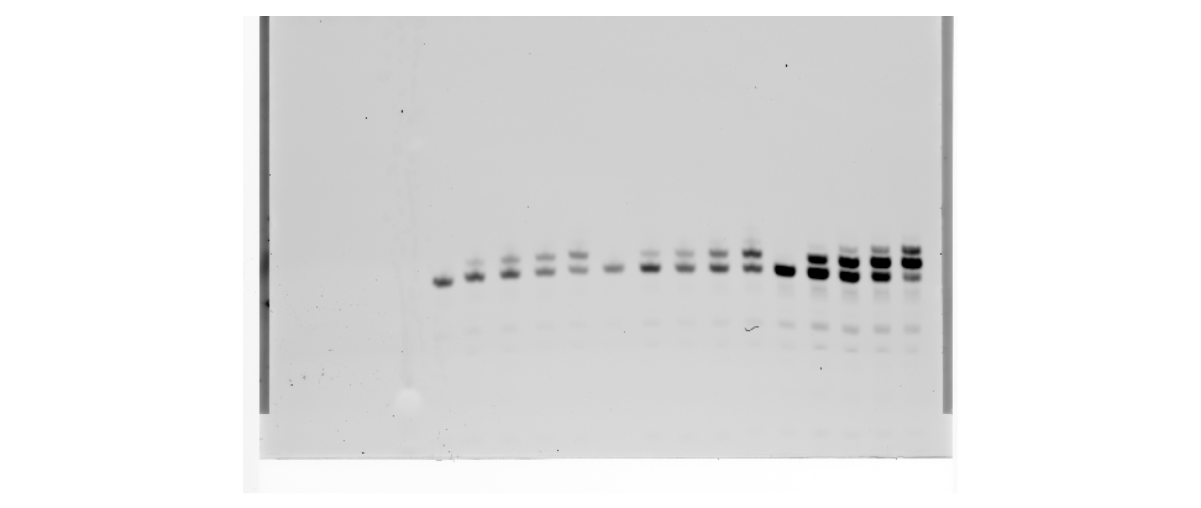 |
|  | C | 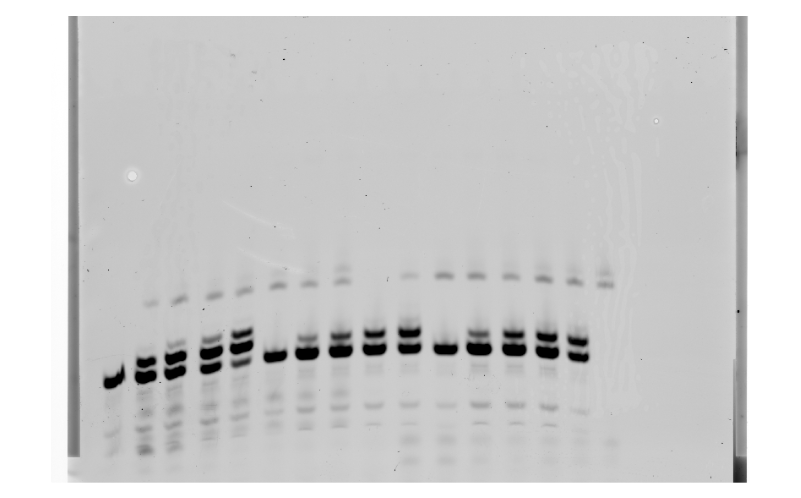 |
|  | D | 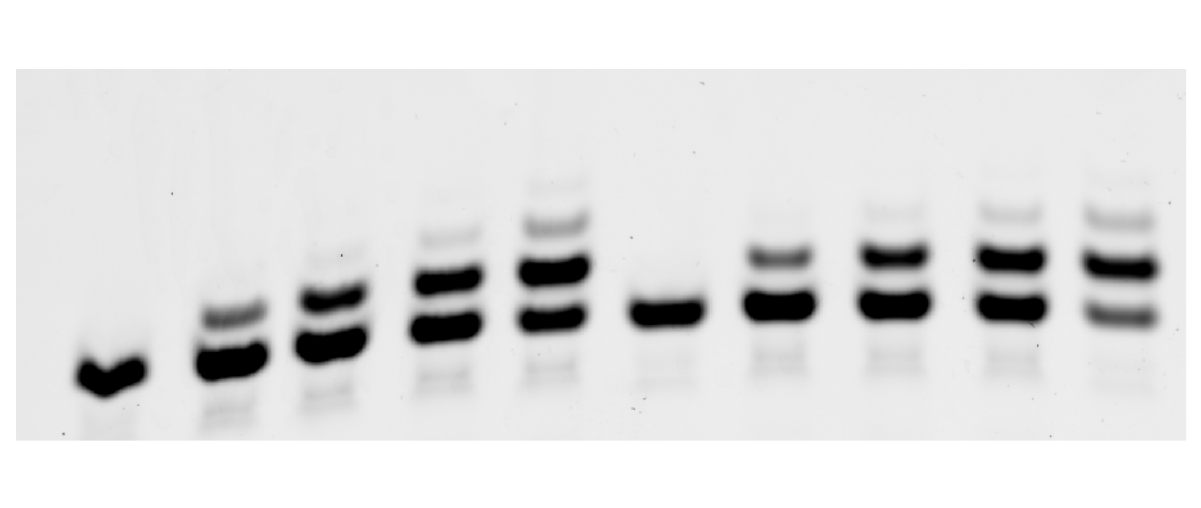 |
| R_6_/rA_12_  40:5 [Arg]:[nt]^a^ | A | 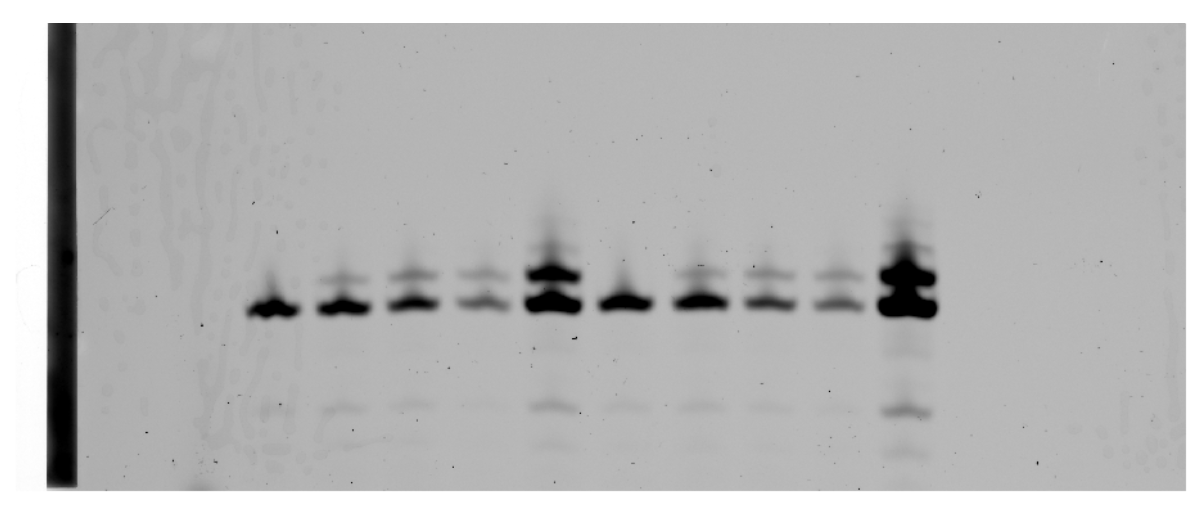 |
|  | B | 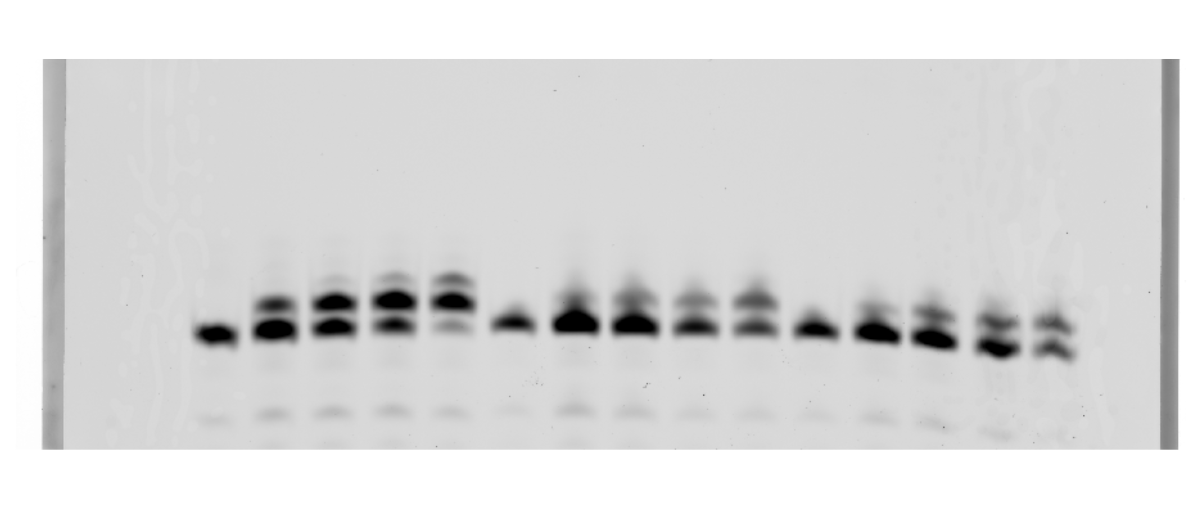 |
|  | C | 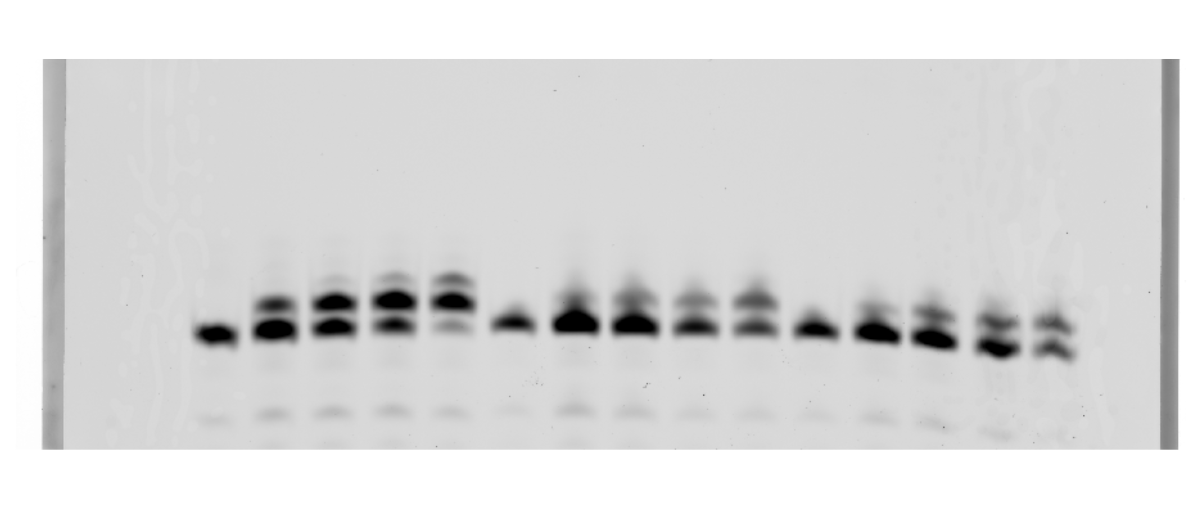 |
|  | D | 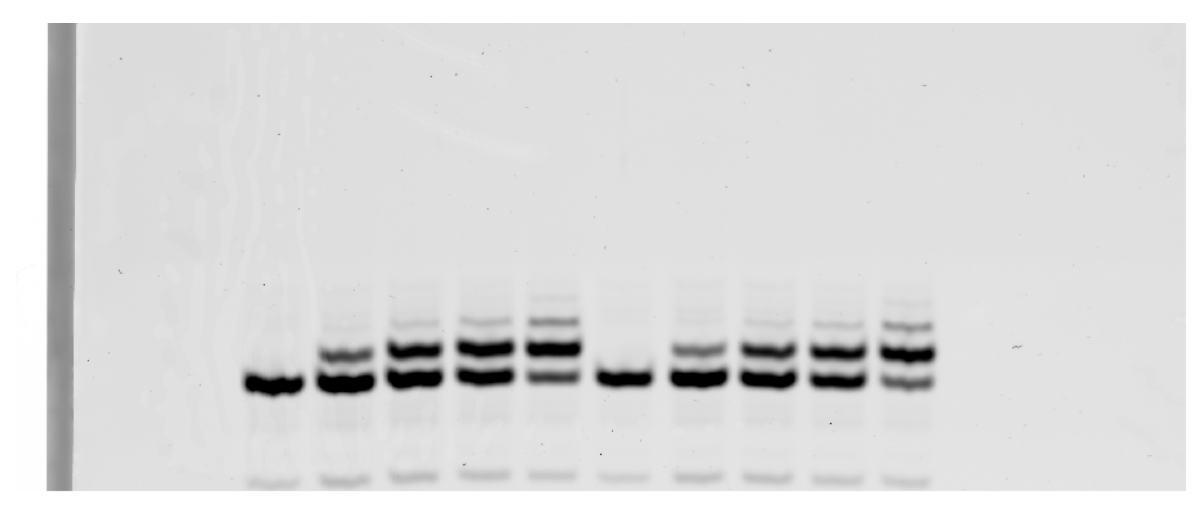 |
| R_6_/mA_12_  40:5 [Arg]:[nt]^a^ | A | 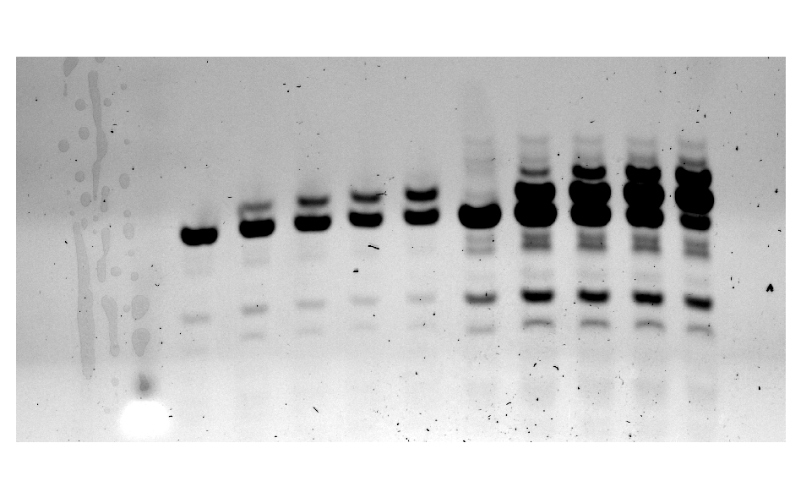 |
|  | B | 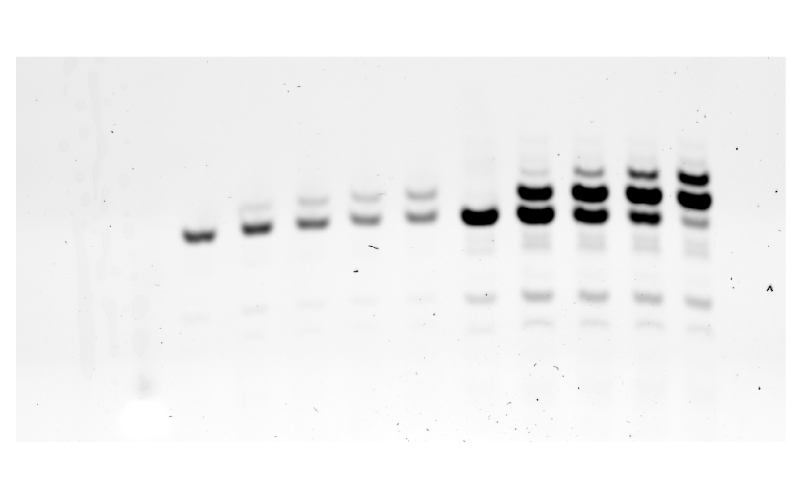 |
|  | C | 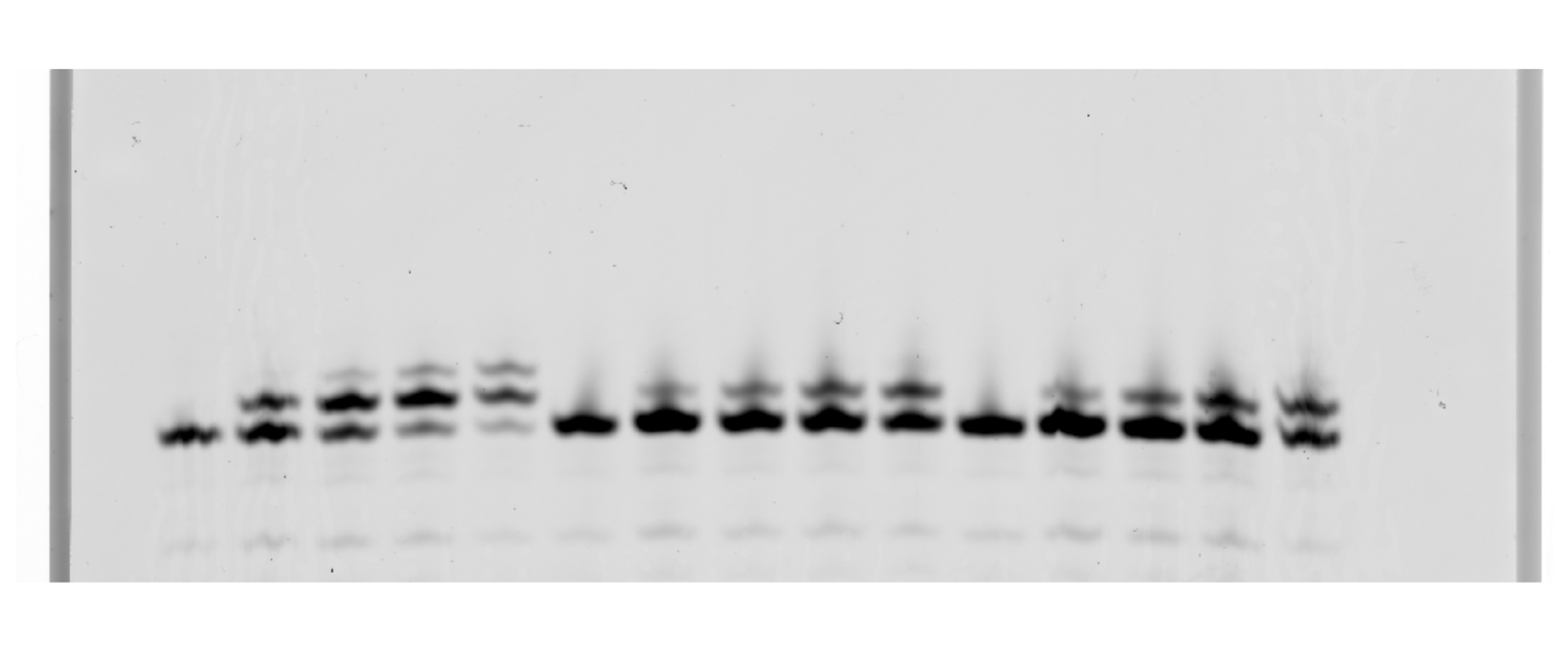 |
|  | D | 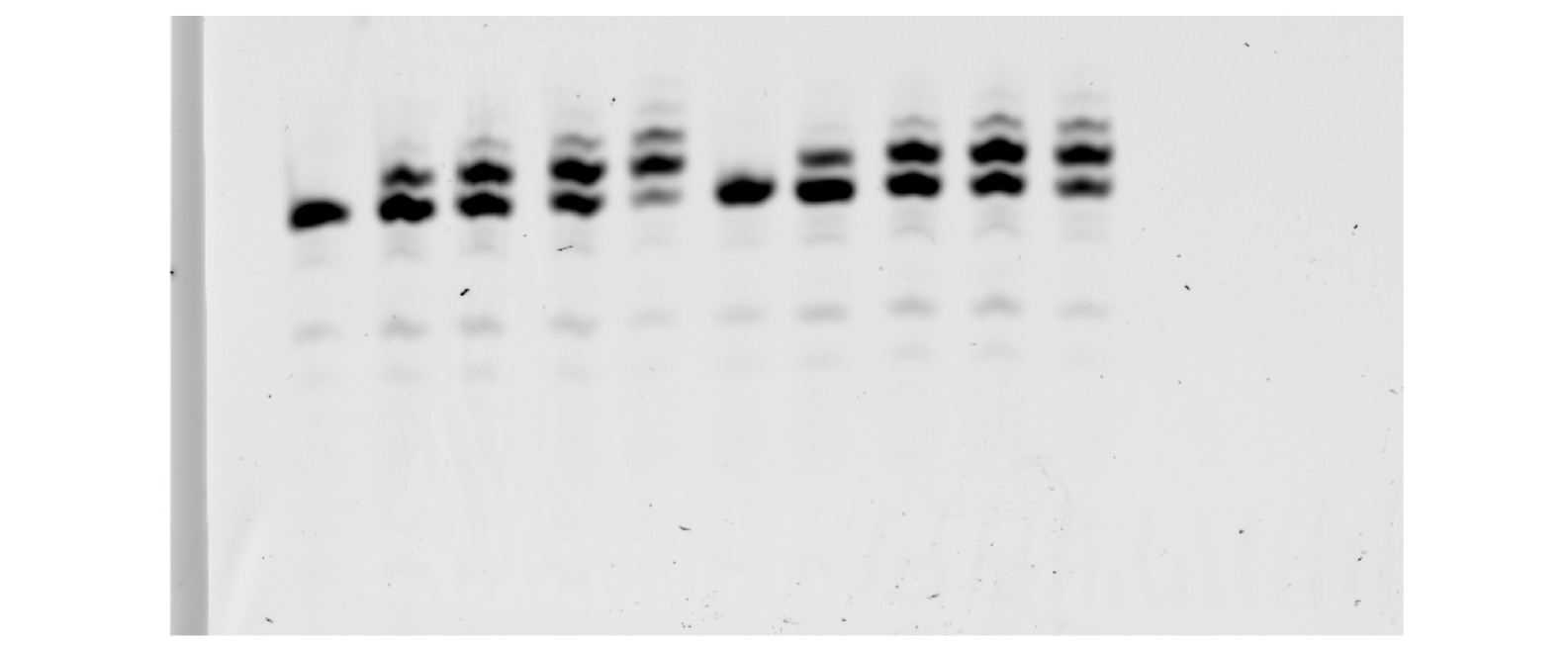 |
| R_6_/dA_16_  20:10 [Arg]:[nt]^a^ | A | 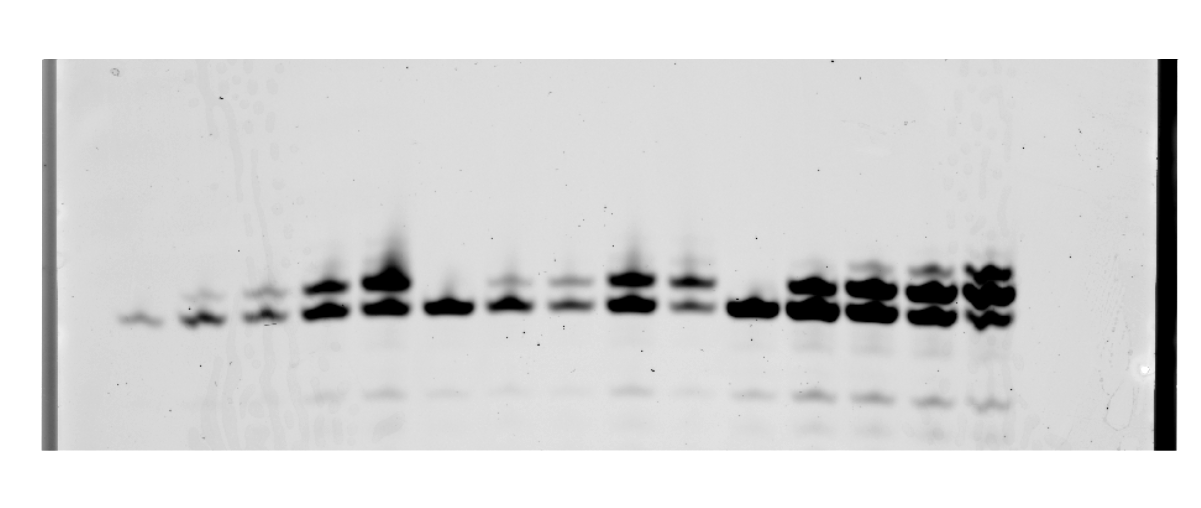 |
|  | B | 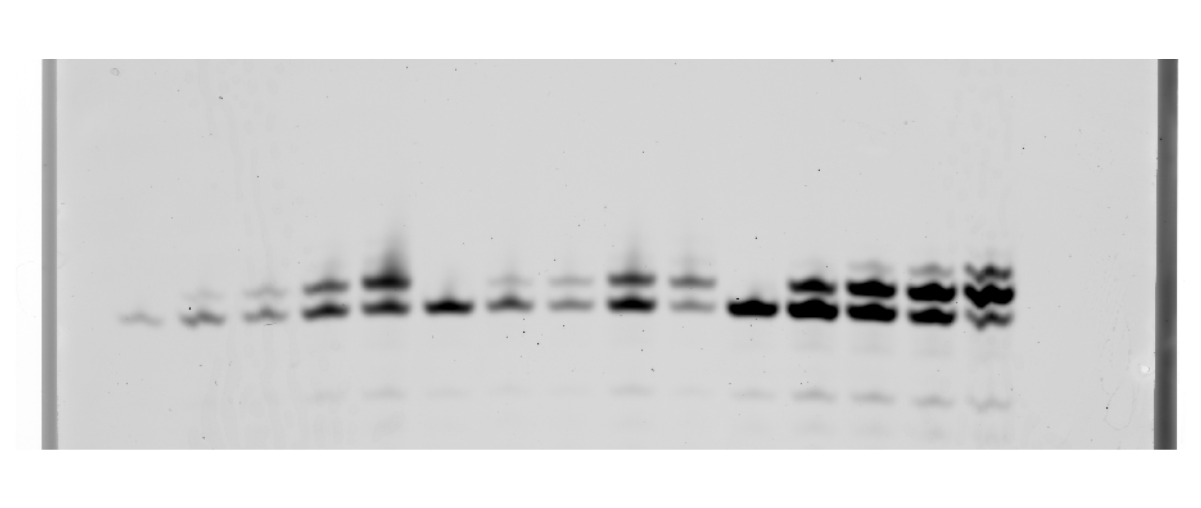 |
|  | C | 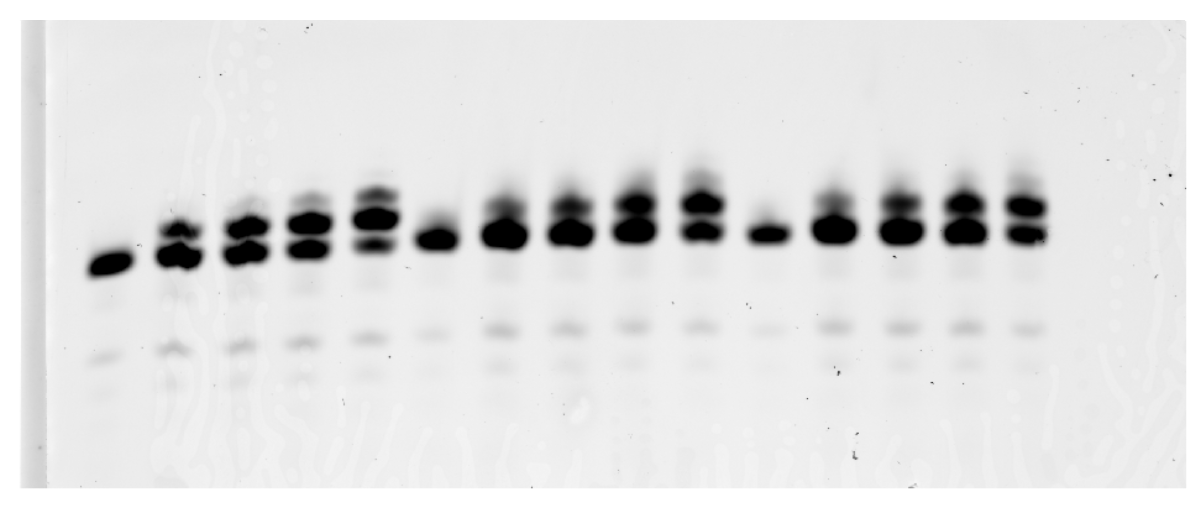 |
|  | D | 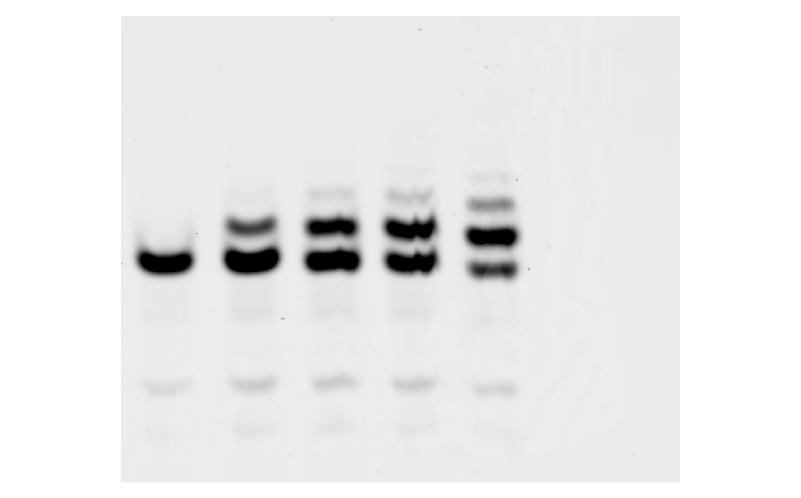 |
| R_6_/rA_16_  20:10 [Arg]:[nt]^a^ | A | 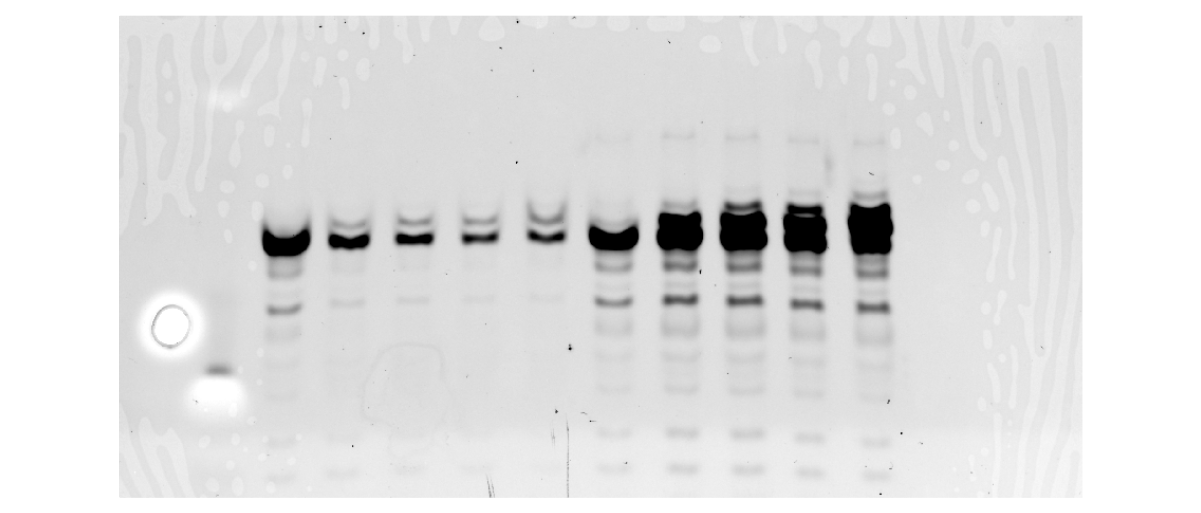 |
|  | B | 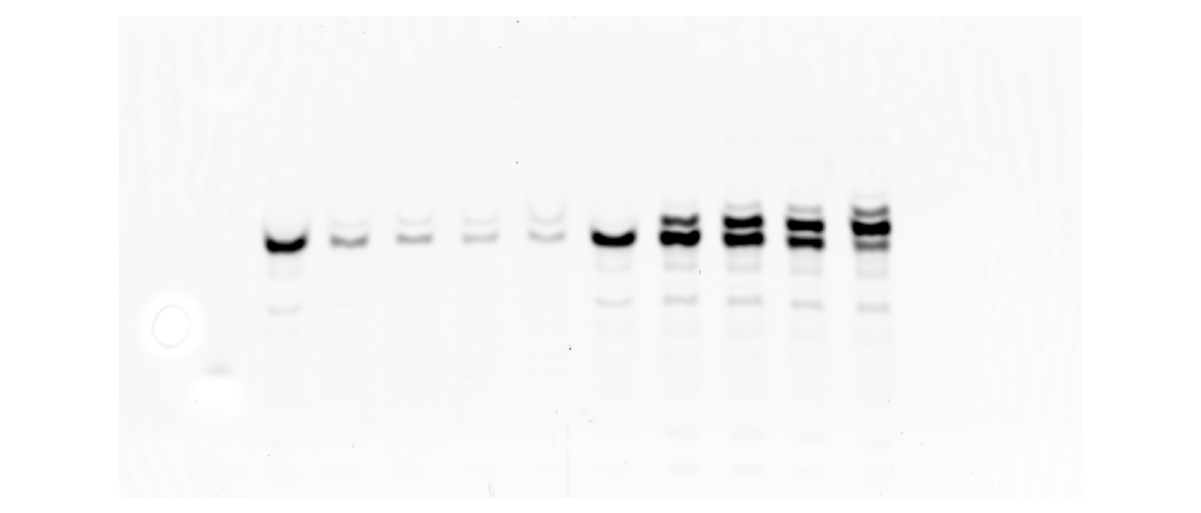 |
|  | C | 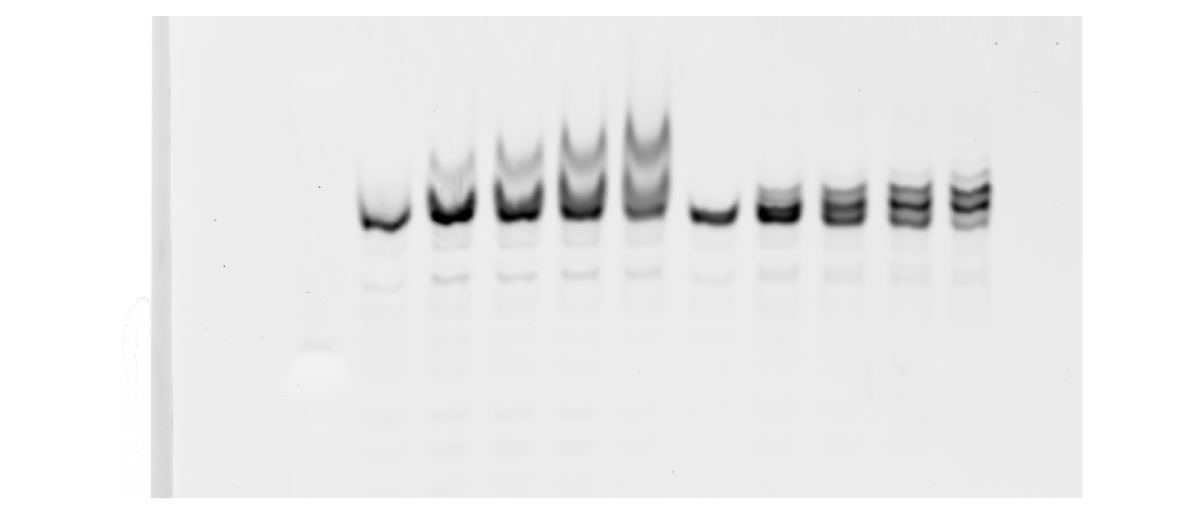 |
|  | D | 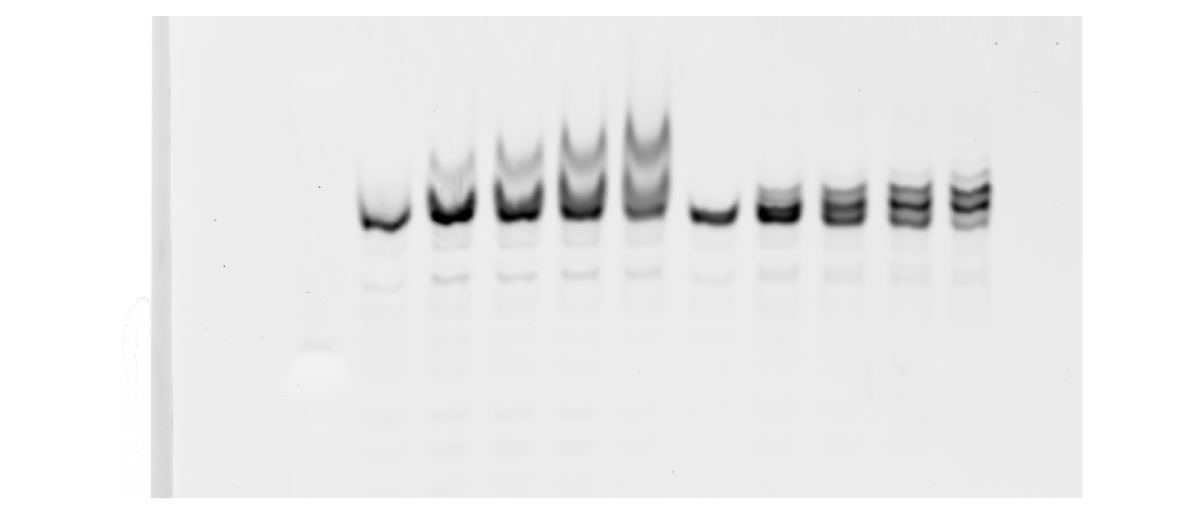 |
| R_4_/dT_16_  20:10 [Arg]:[nt] | A | 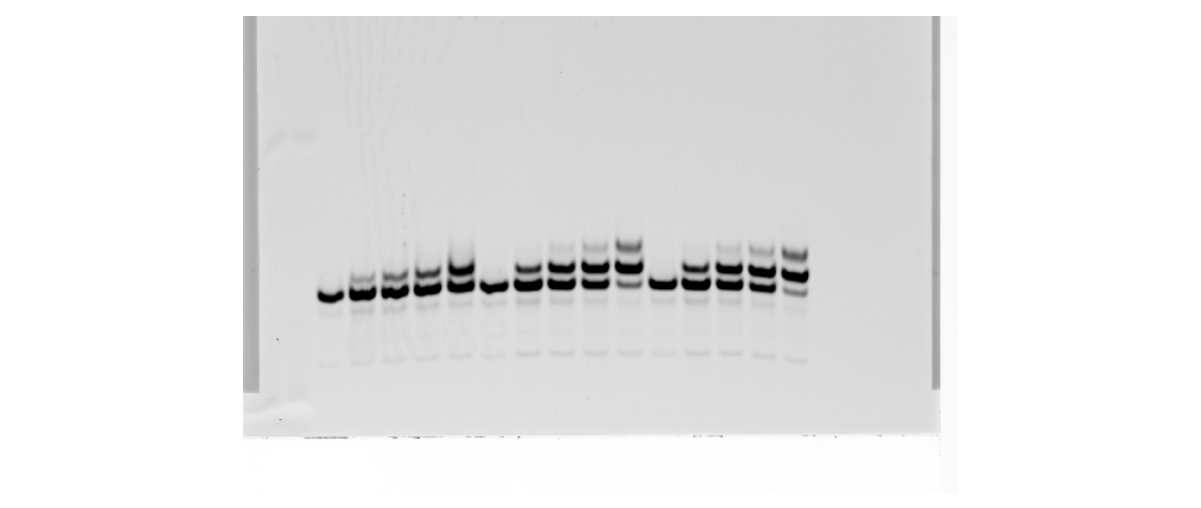 |
|  | B | 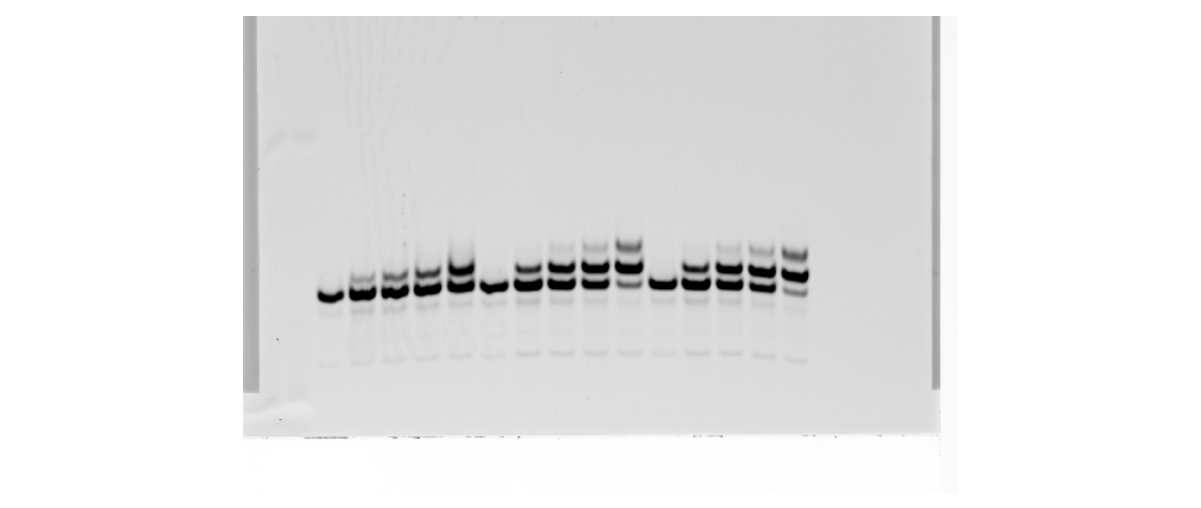 |
|  | C | 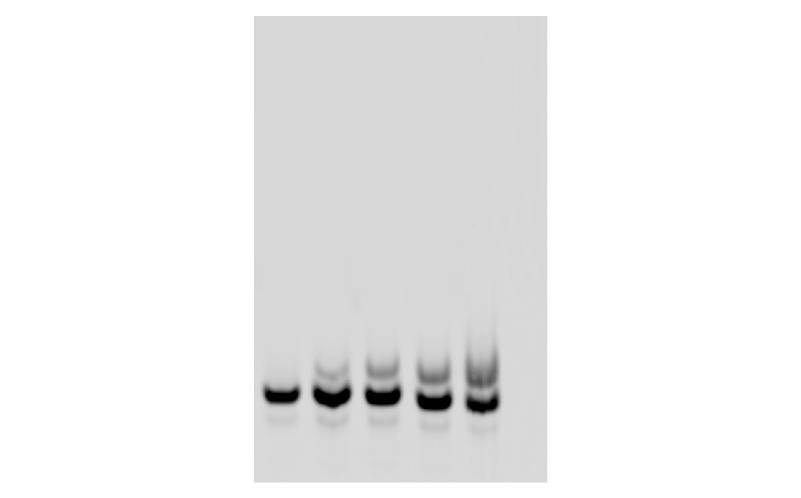 |
|  | D | 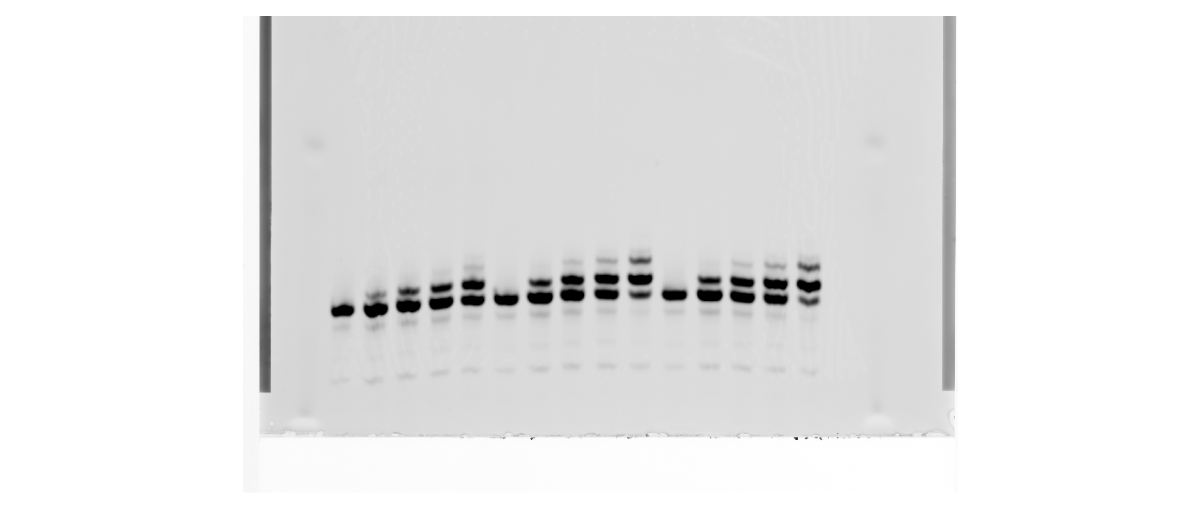 |
| R_6_/dT_16_  20:10 [Arg]:[nt] | A | 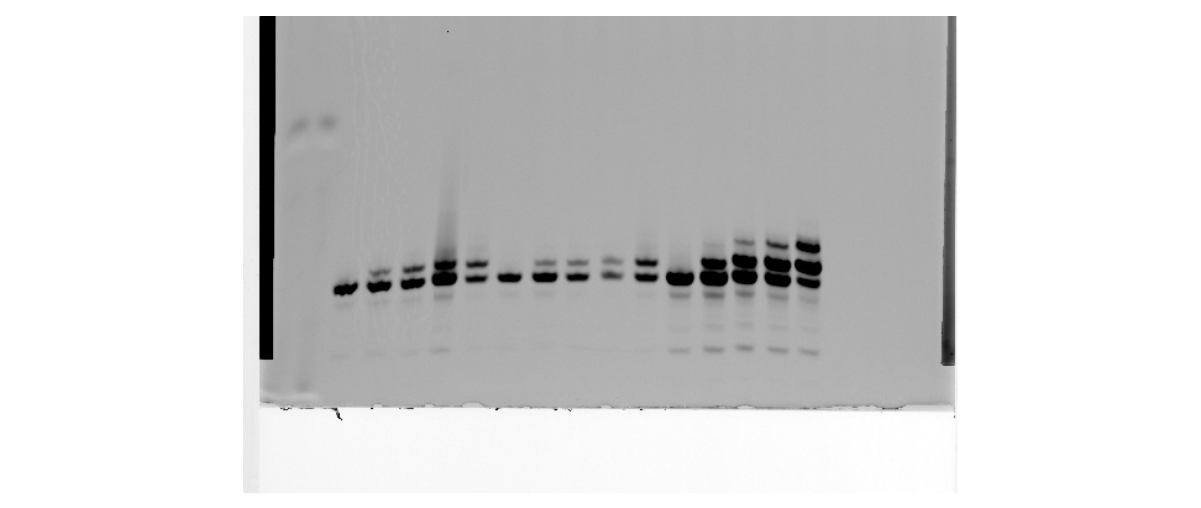 |
|  | B | 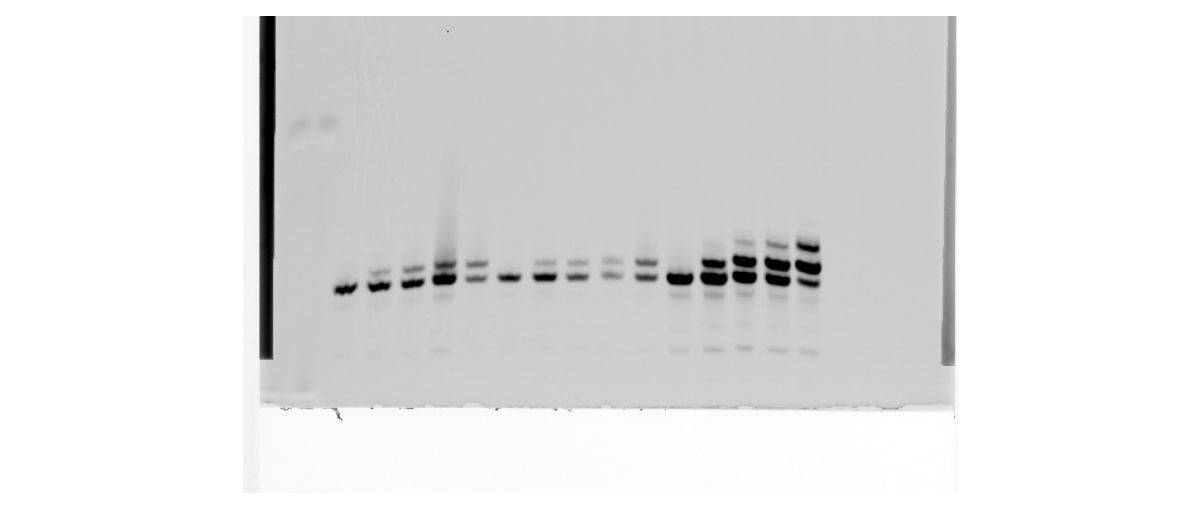 |
|  | C | 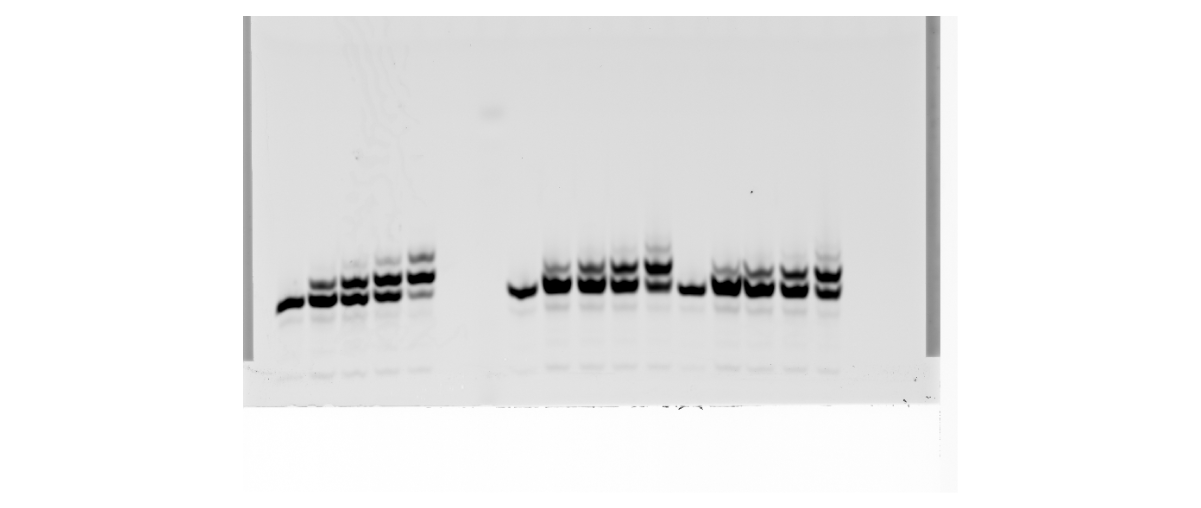 |
|  | D | 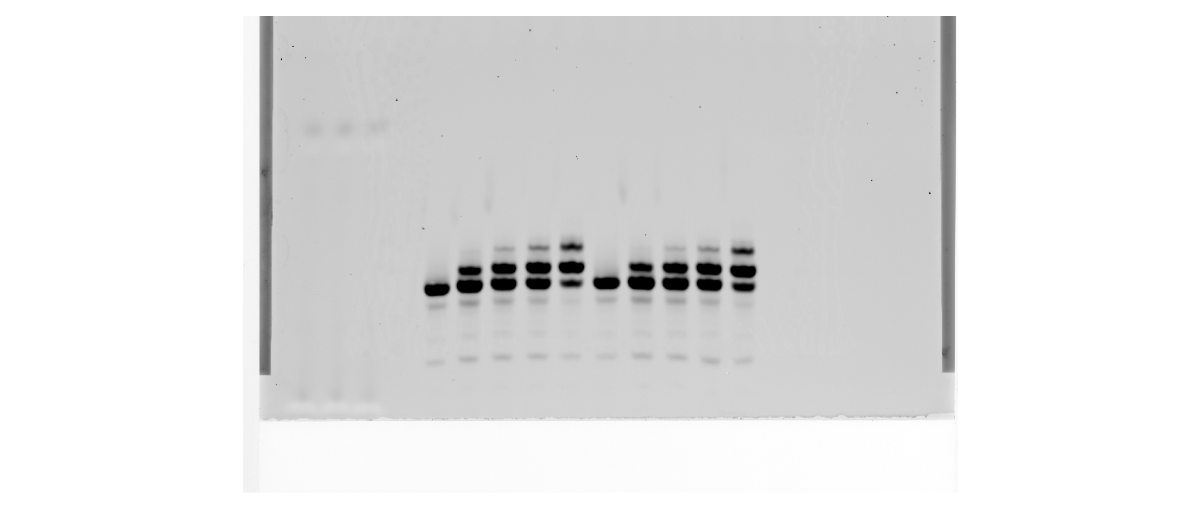 |
| R_4_/DNA_12_  20:10 [Arg]:[nt] | A | 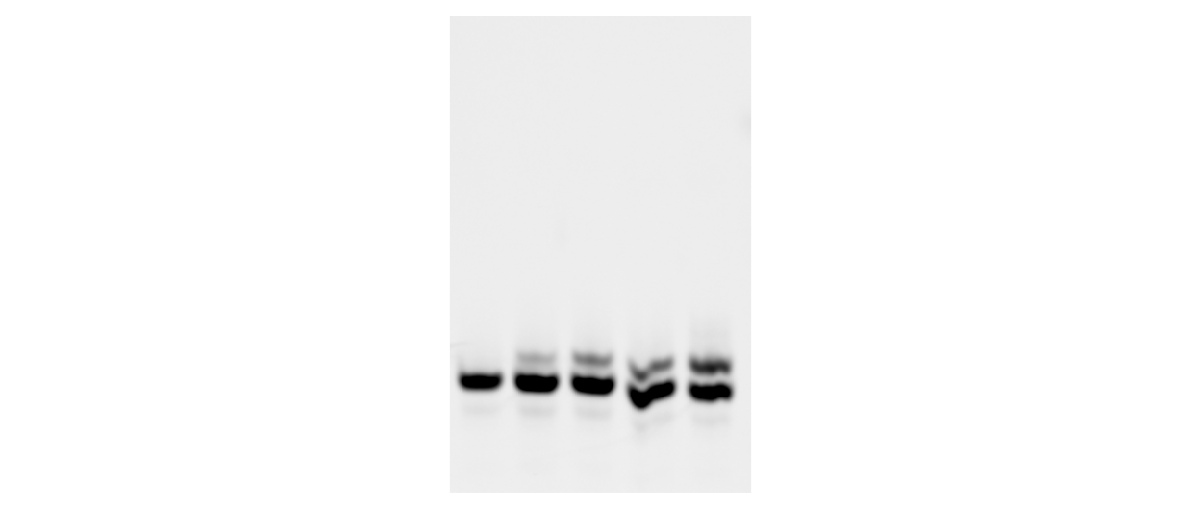 |
|  | B | 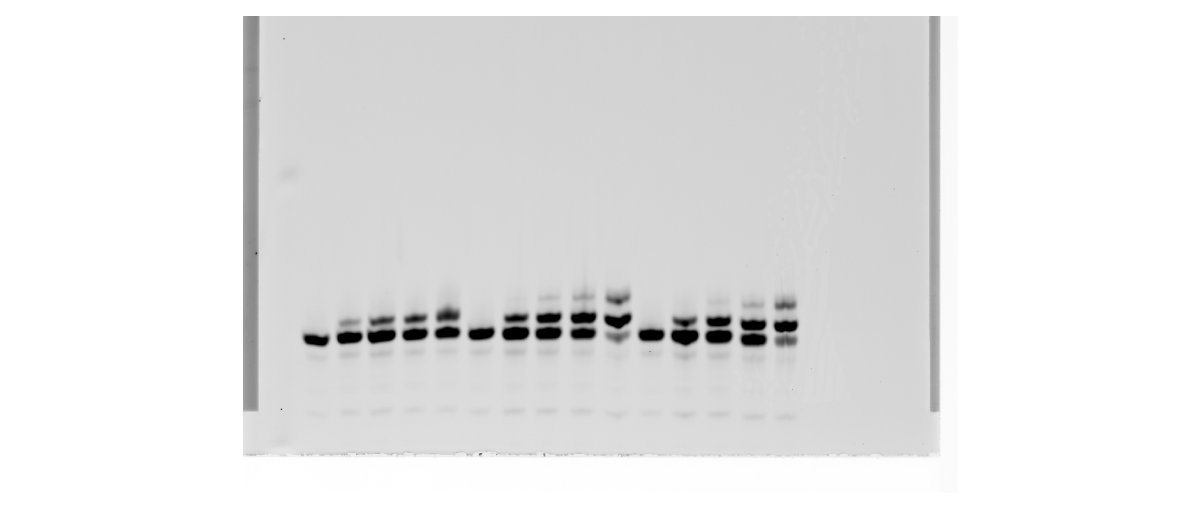 |
|  | C | 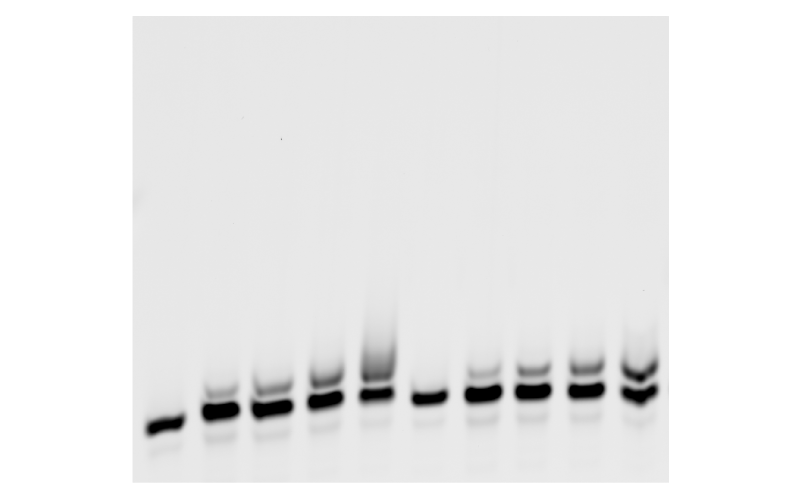 |
|  | D | 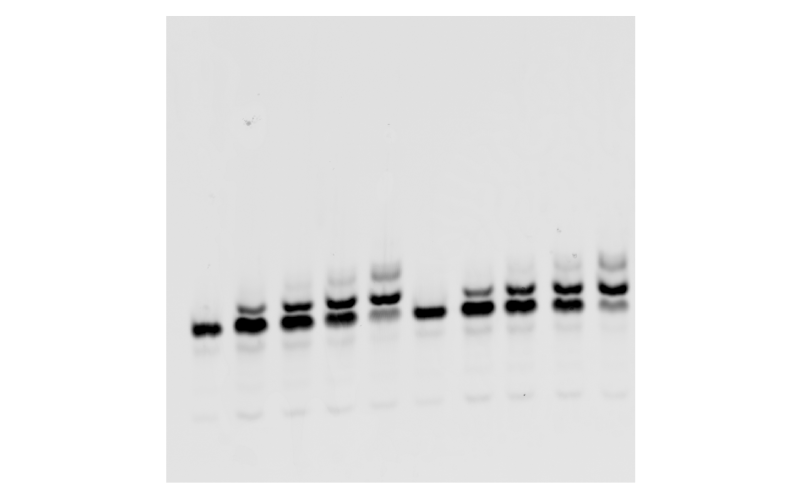 |
| R_4_/DNA_12_  40:5 [Arg]:[nt]^a^ | A | 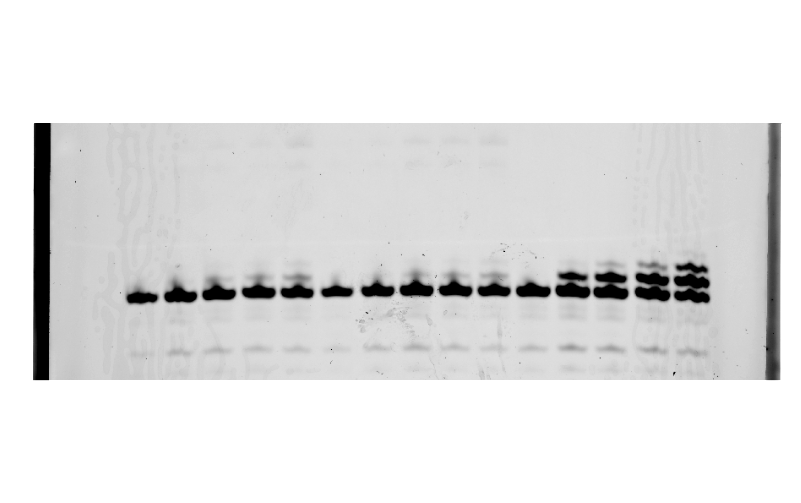 |
|  | B | 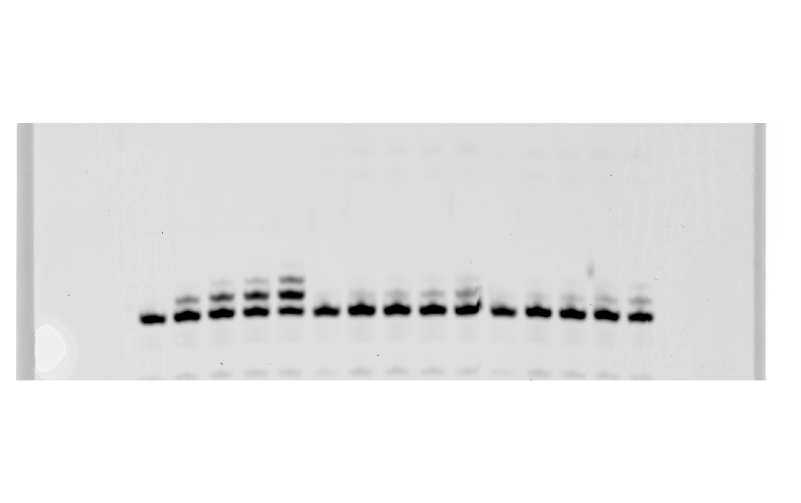 |
|  | C | 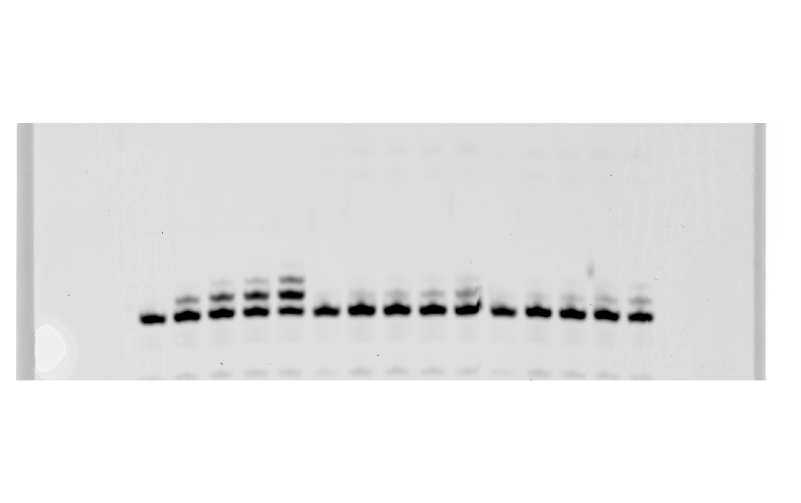 |
|  | D | 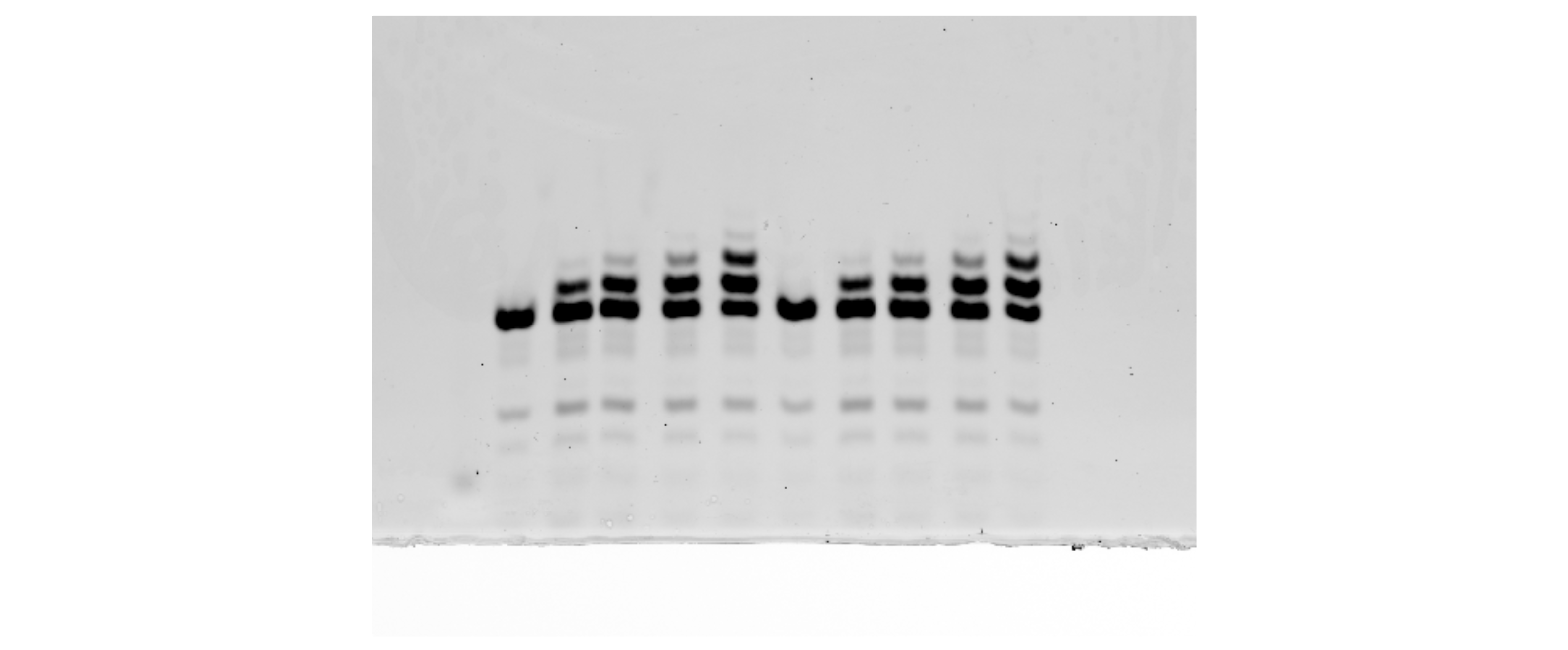 |
| R_4_/DNA_12_  40:5 [Arg]:[nt] | A | 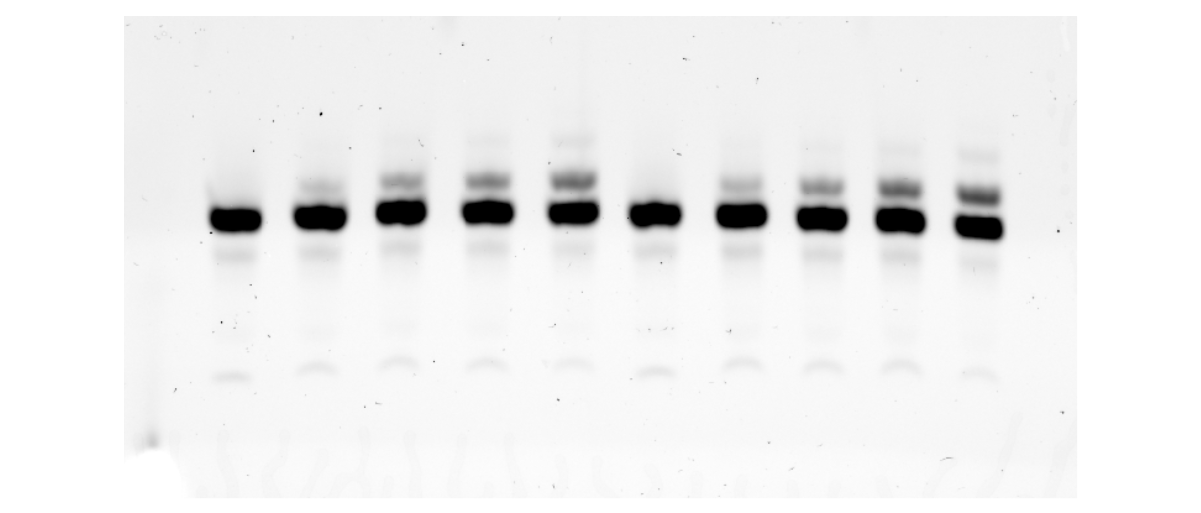 |
|  | B | 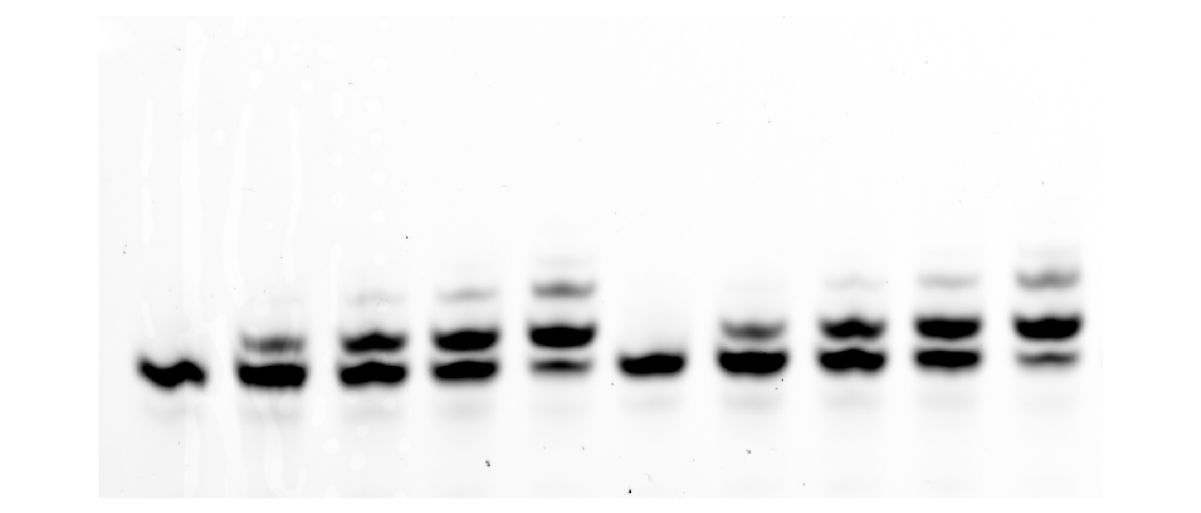 |
|  | C | 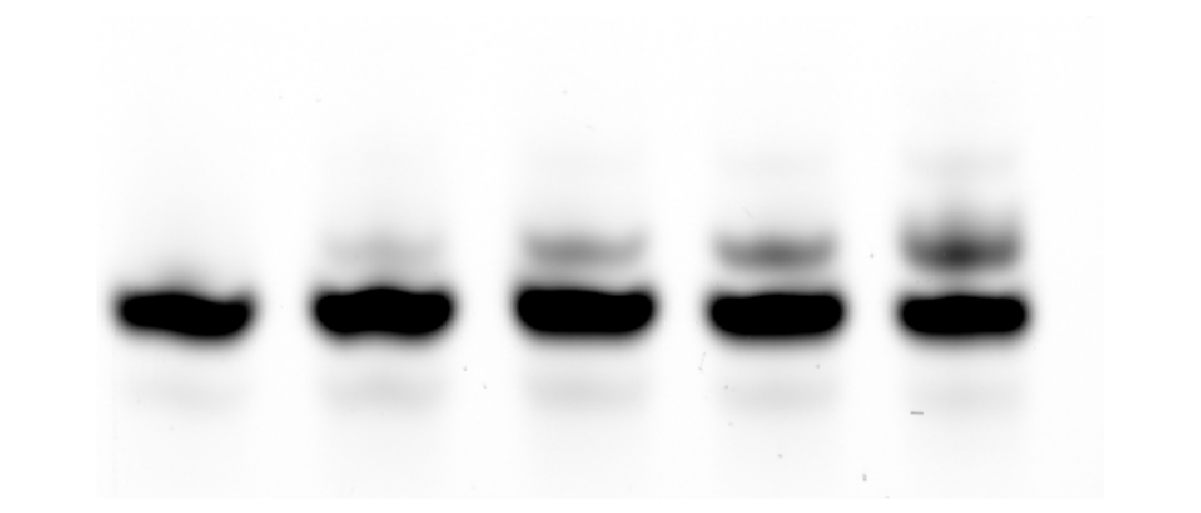 |
|  | D | 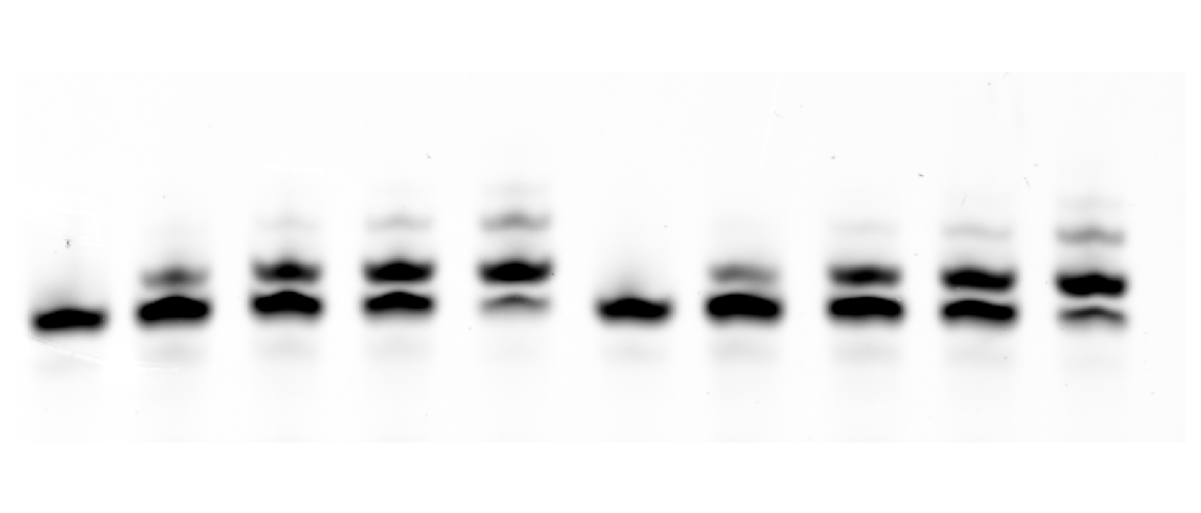 |
| R_6_/DNA_12_  20:10 [Arg]:[nt]^a^ | A | 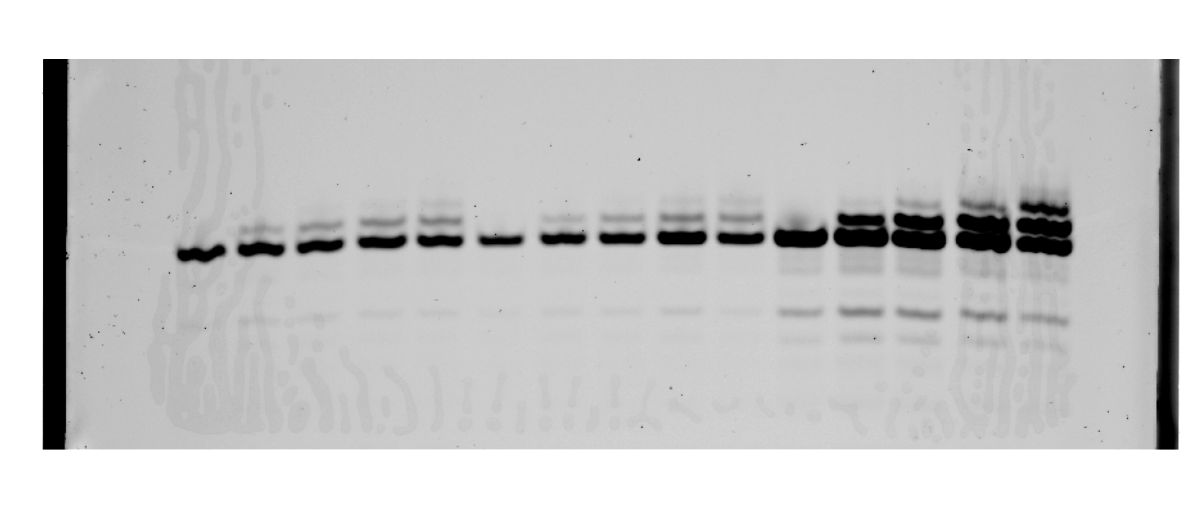 |
|  | B | 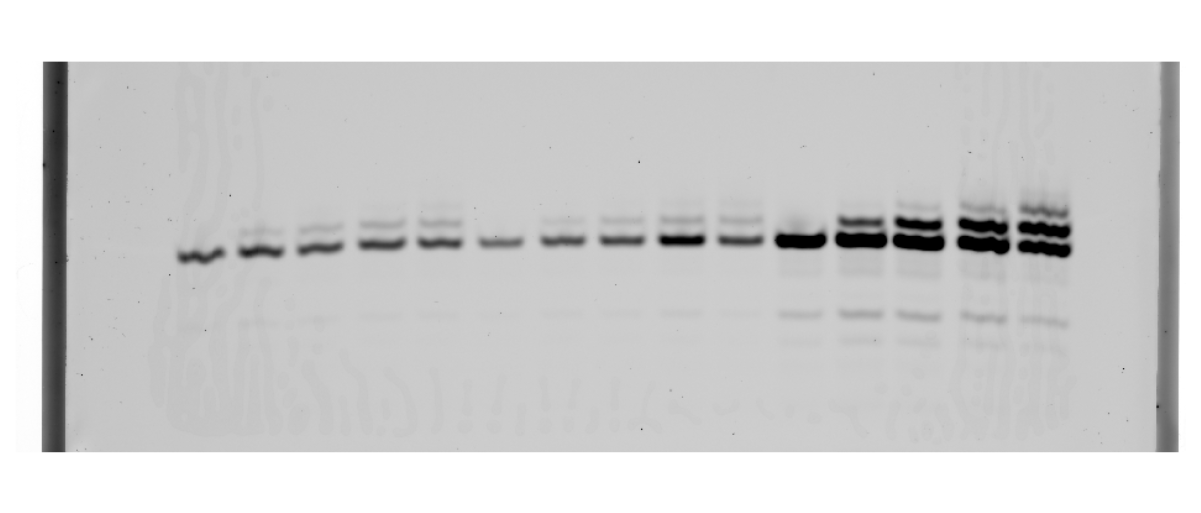 |
|  | C | 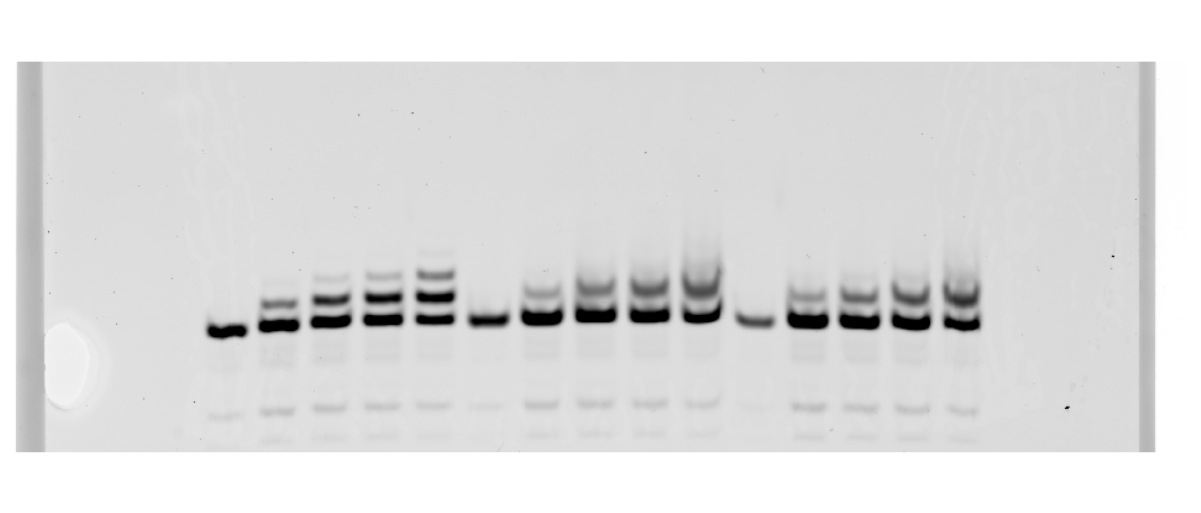 |
|  | D | 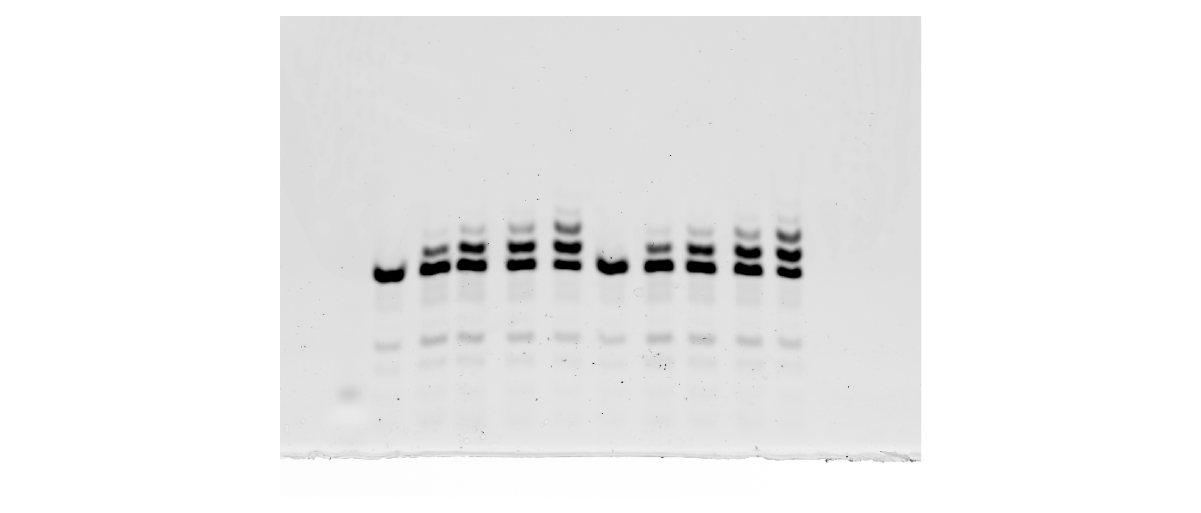 |
| R_6_/DNA_12_  20:10 [Arg]:[nt] | A | 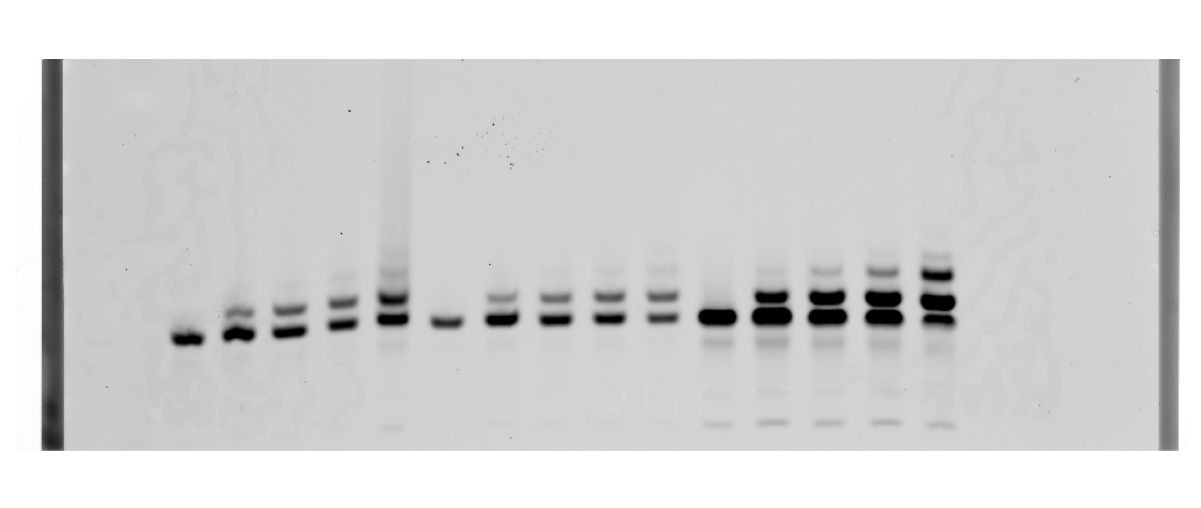 |
|  | B | 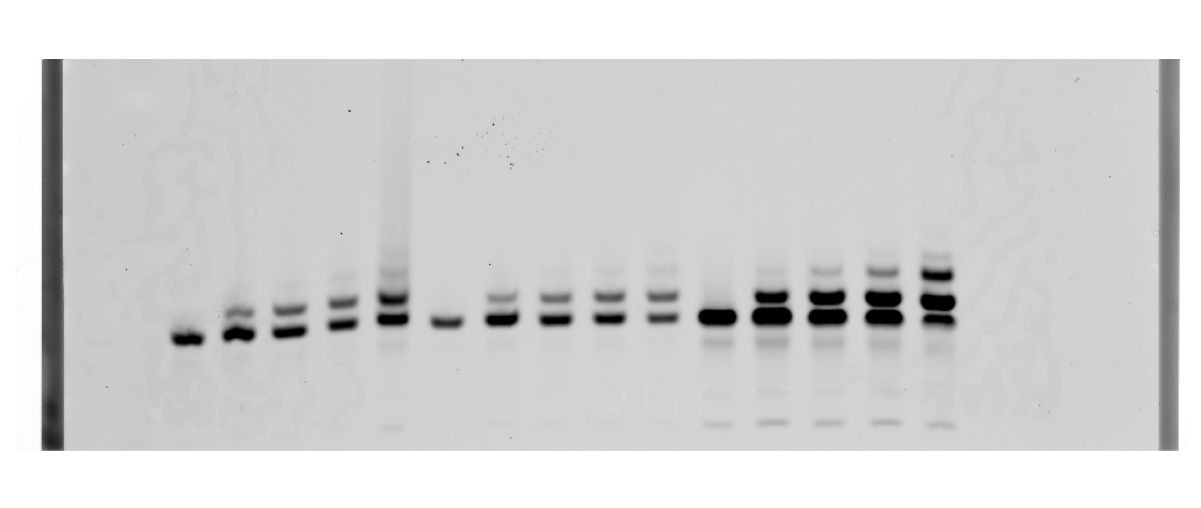 |
|  | C | 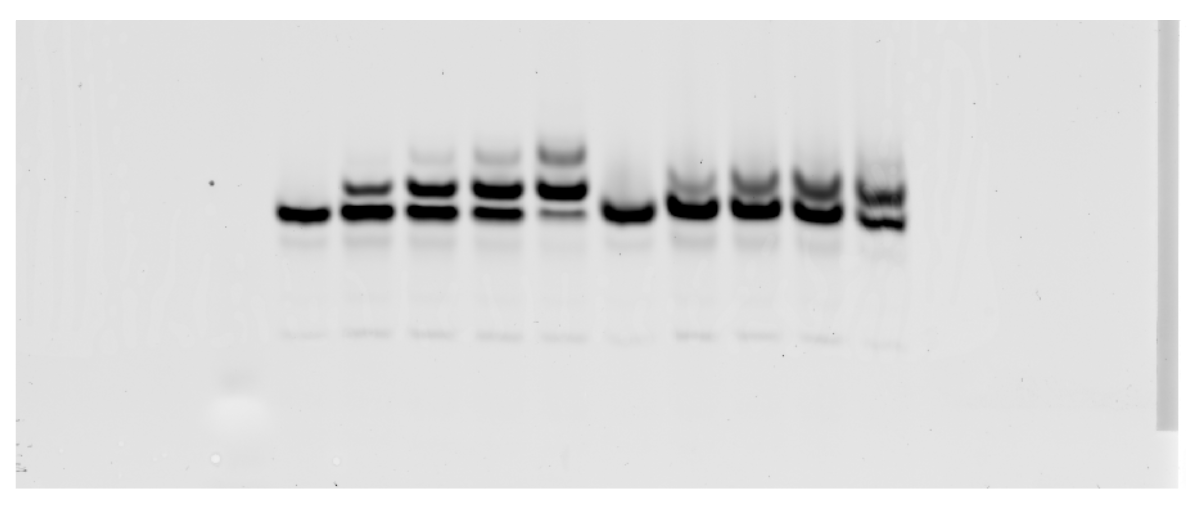 |
|  | D | 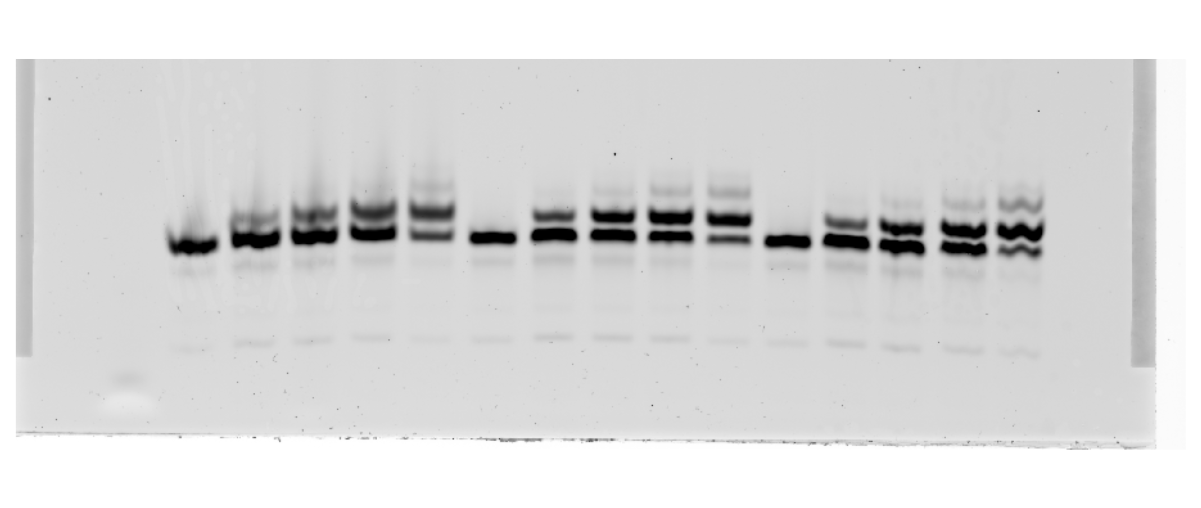 |
| R_6_/DNA_12_  40:5 [Arg]:[nt] | A | 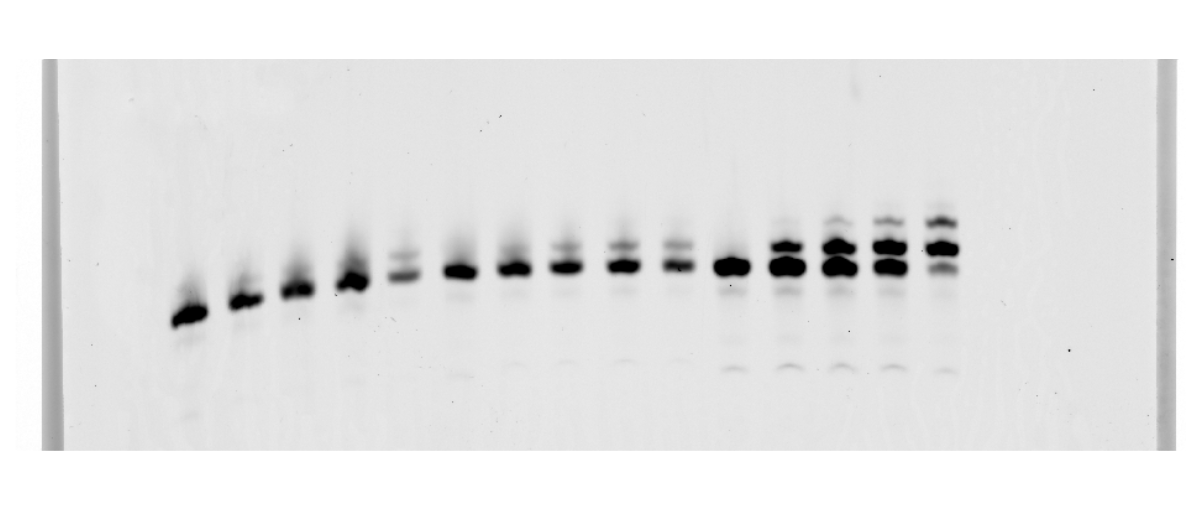 |
|  | B | 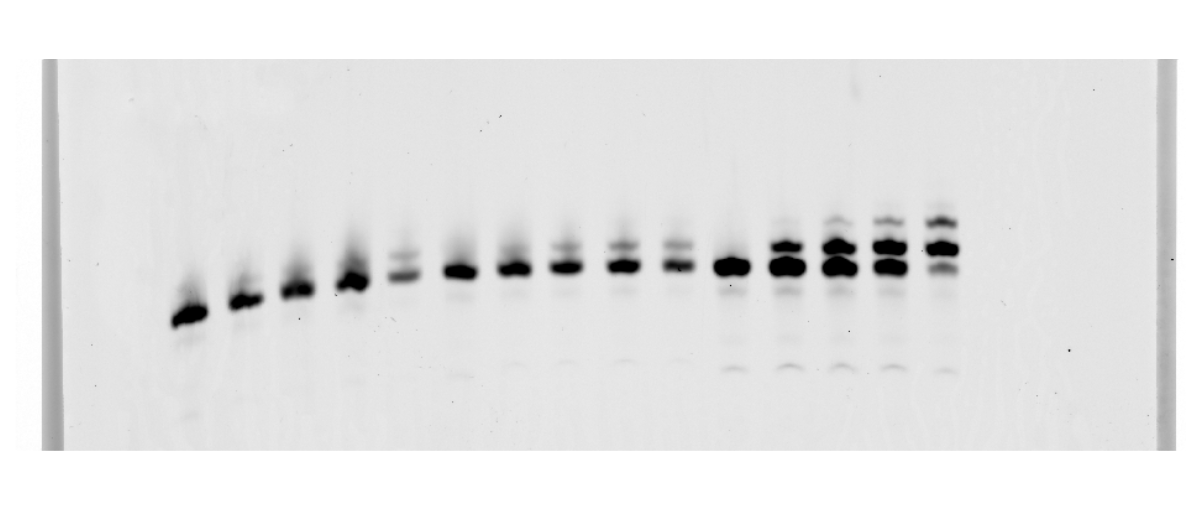 |
|  | C | 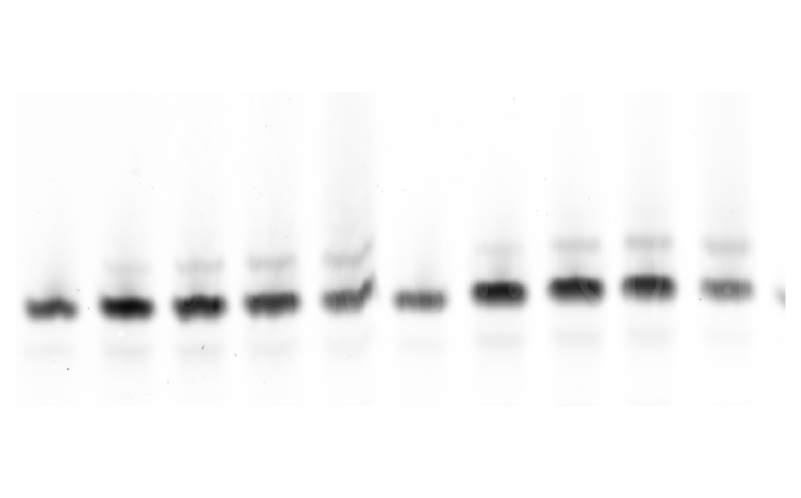 |
|  | D | 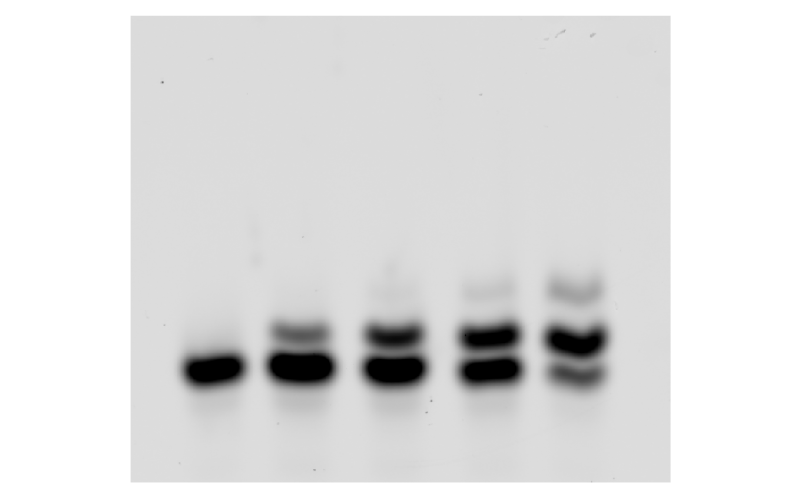 |
| R_4_/RNA_12_  40:5 [Arg]:[nt]^a^ | A | 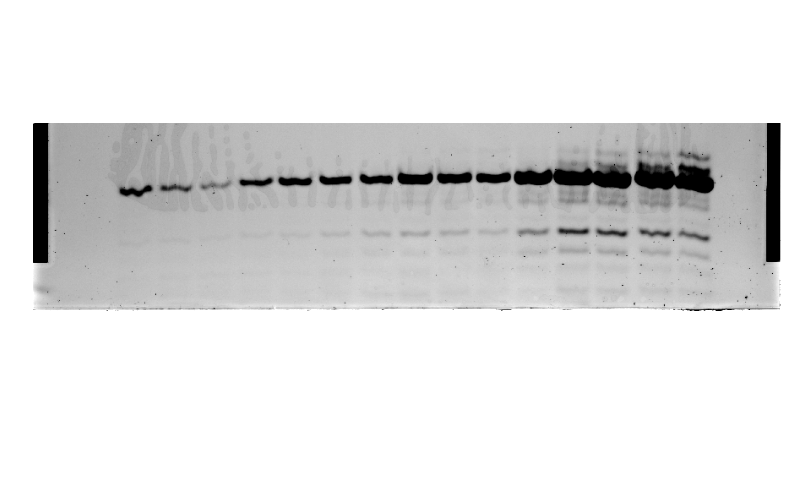 |
|  | B | 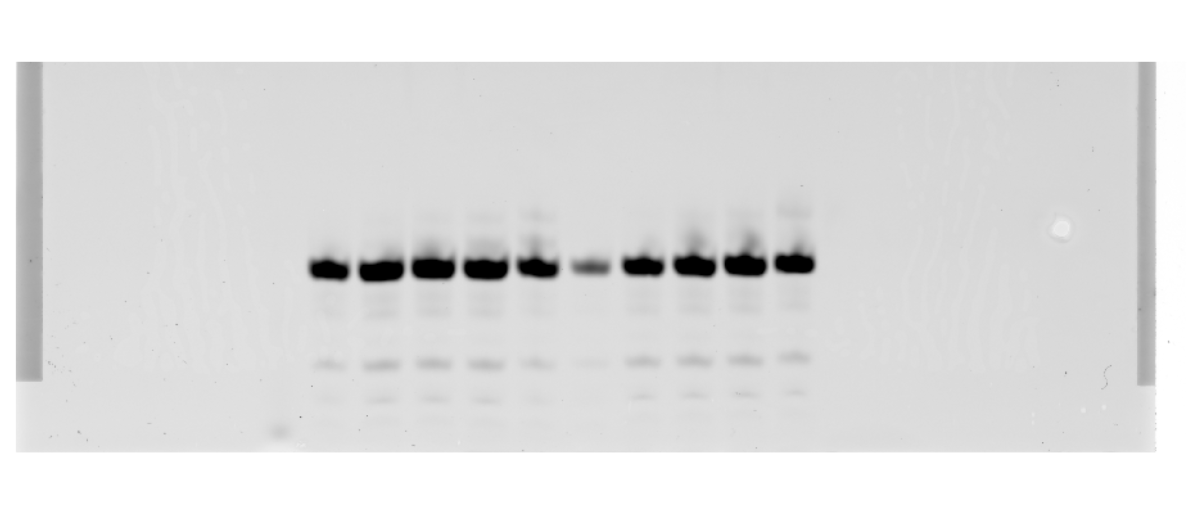 |
|  | C | 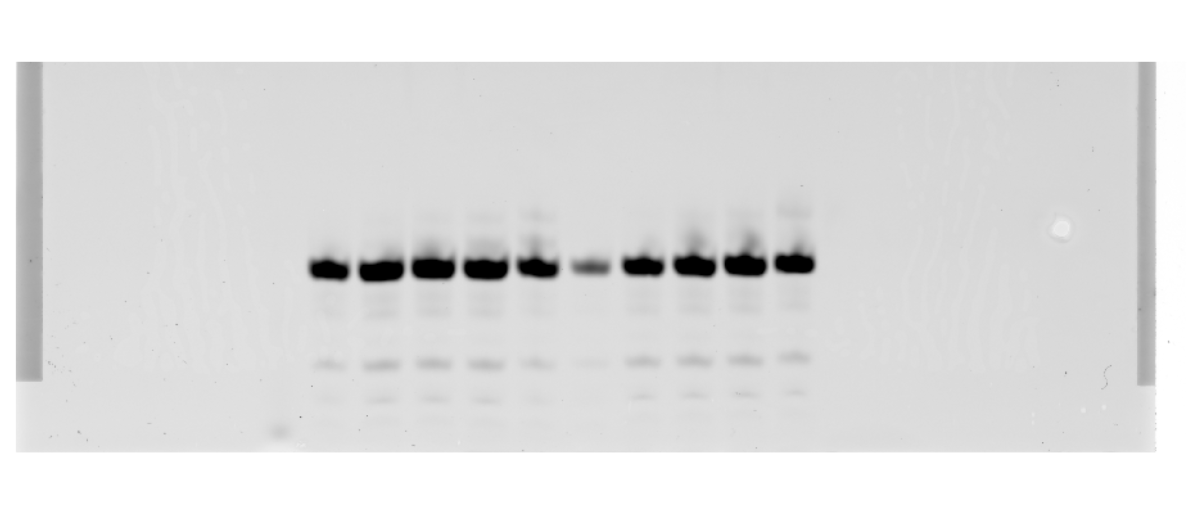 |
|  | D | 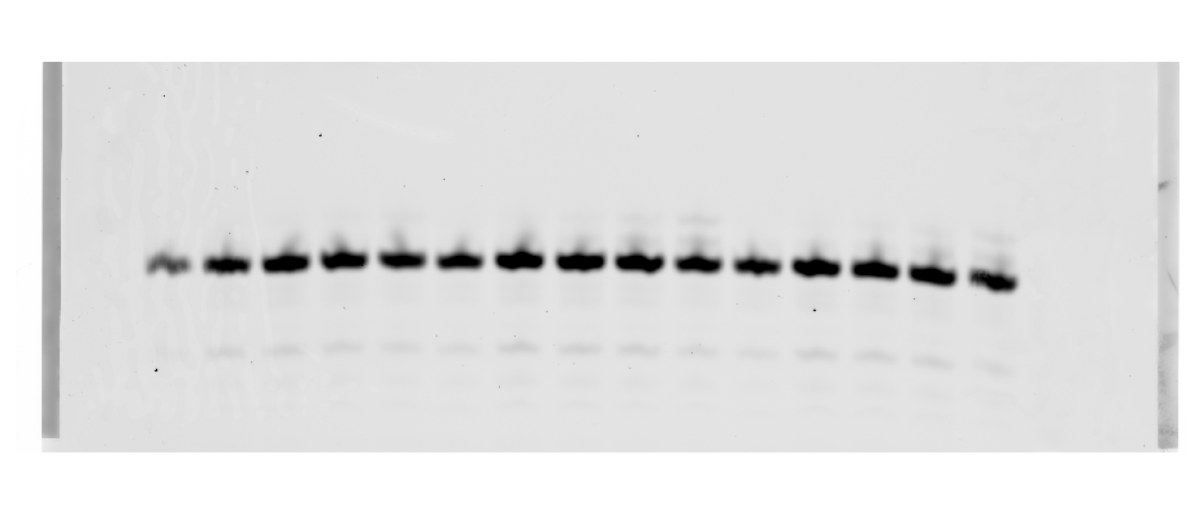 |
| R_4_/RNA_12_  40:5 [Arg]:[nt] | A | 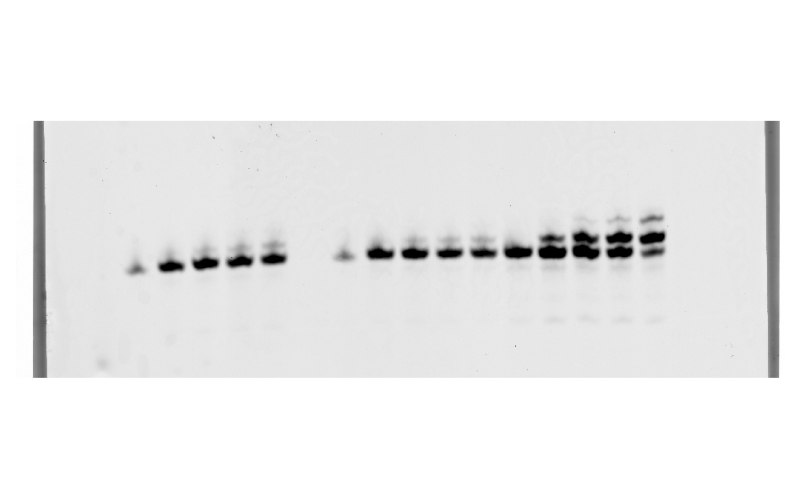 |
|  | B | 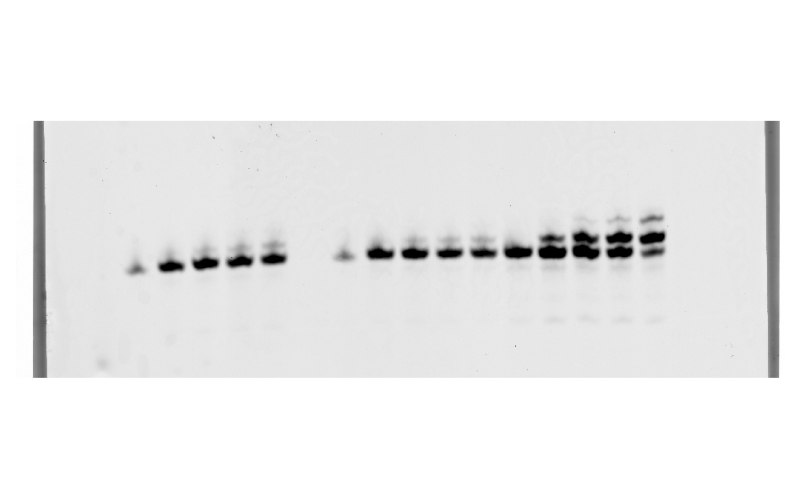 |
|  | C | 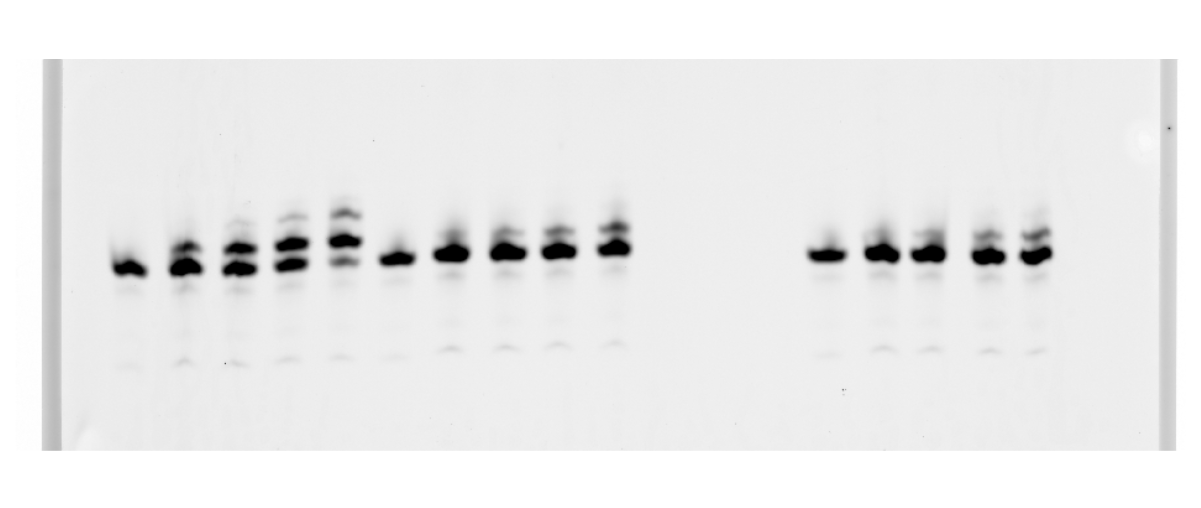 |
|  | D | 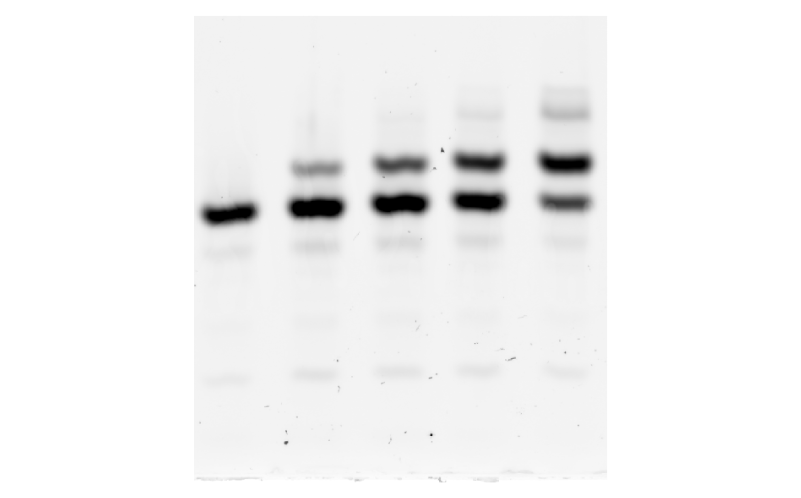 |
| R_6_/RNA_12_  20:10 [Arg]:[nt] | A | 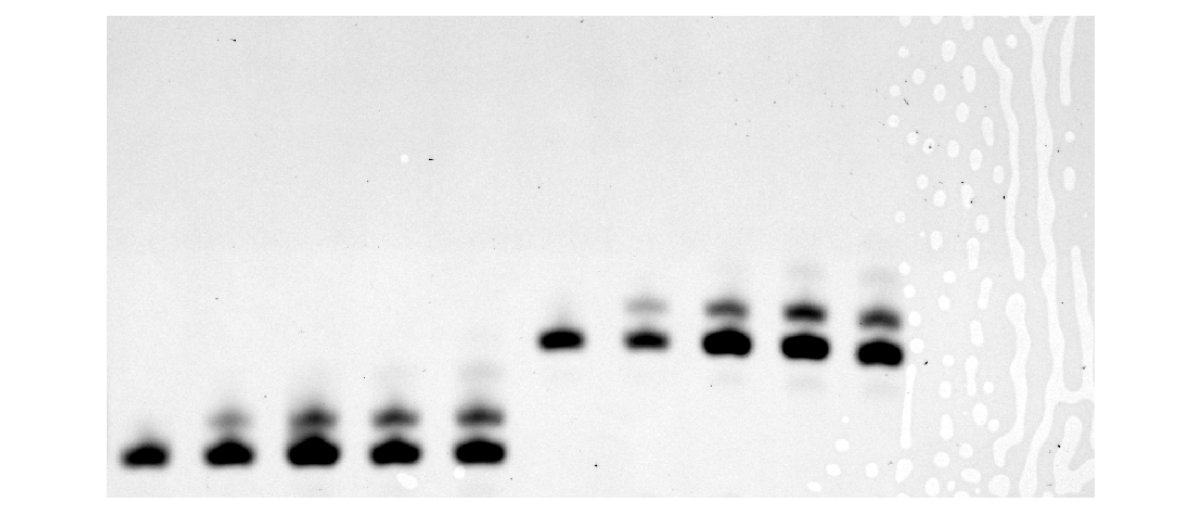 |
|  | B | 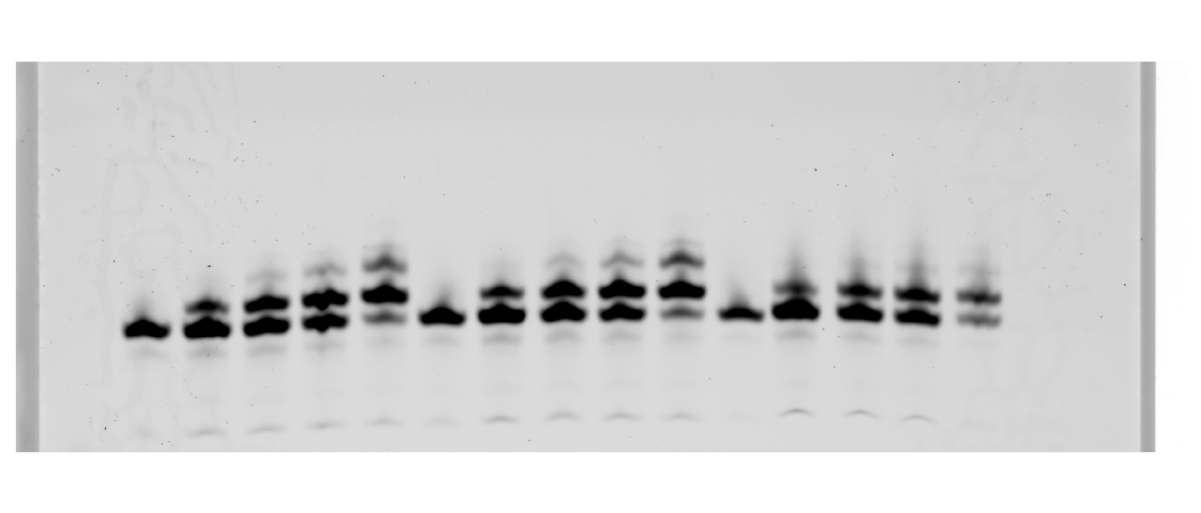 |
|  | C | 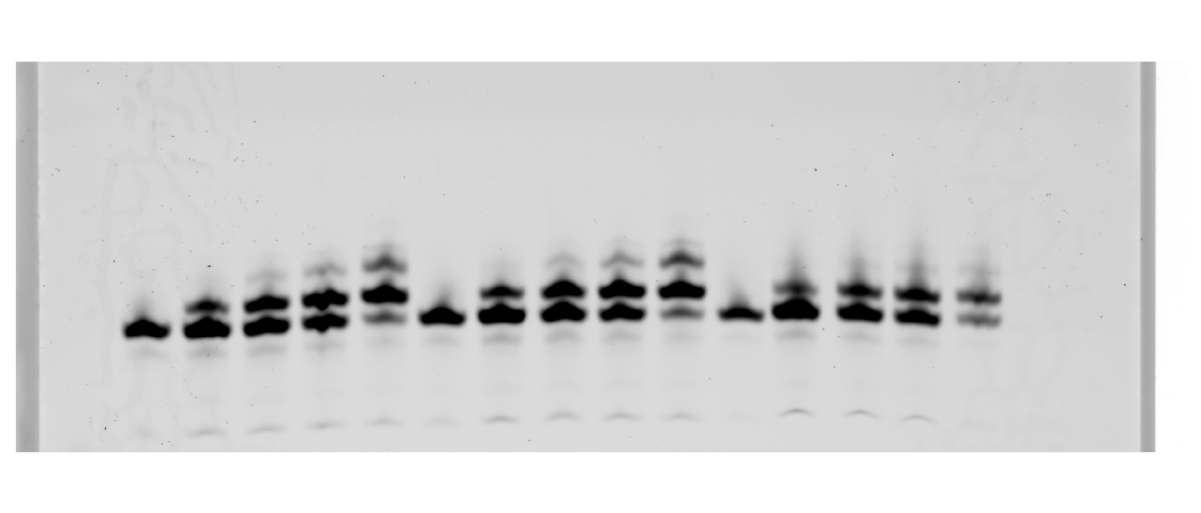 |
|  | D | 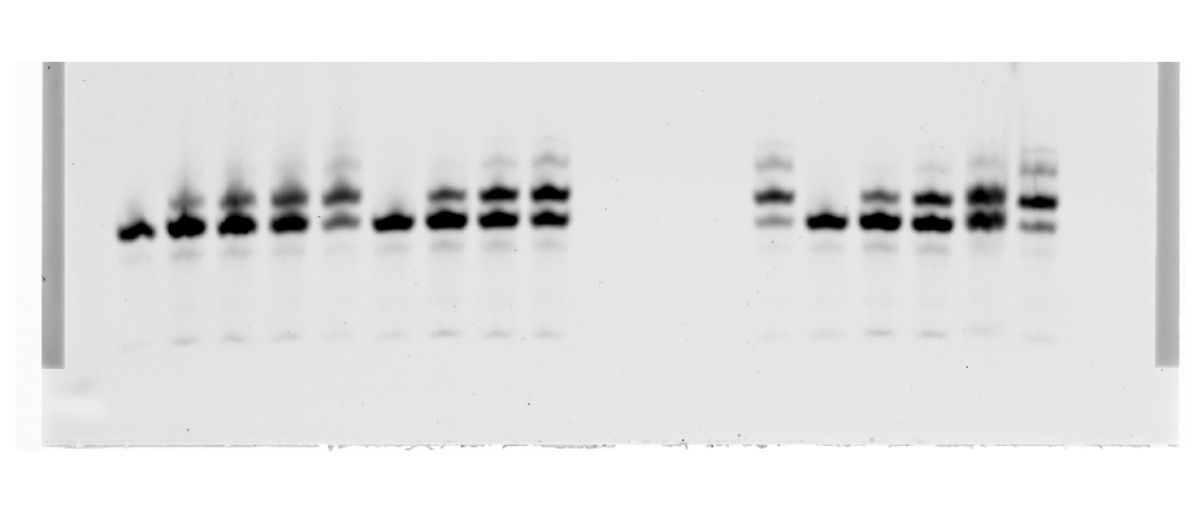 |
| R_6_/RNA_12_  40:5 [Arg]:[nt] | A | 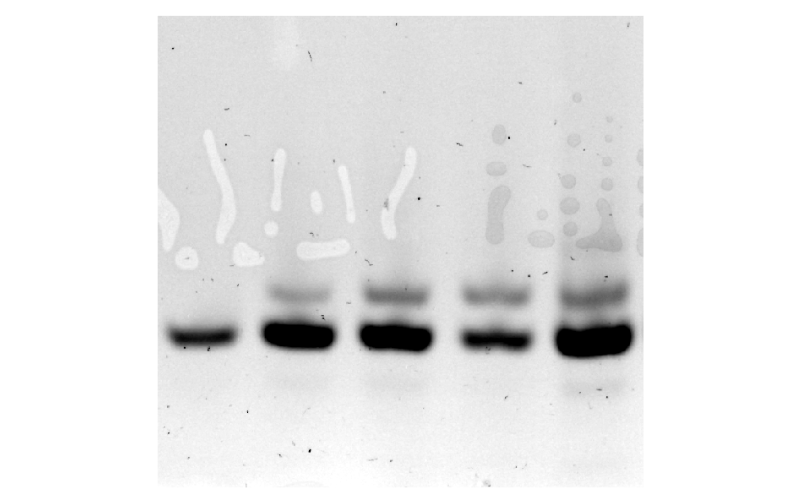 |
|  | B | 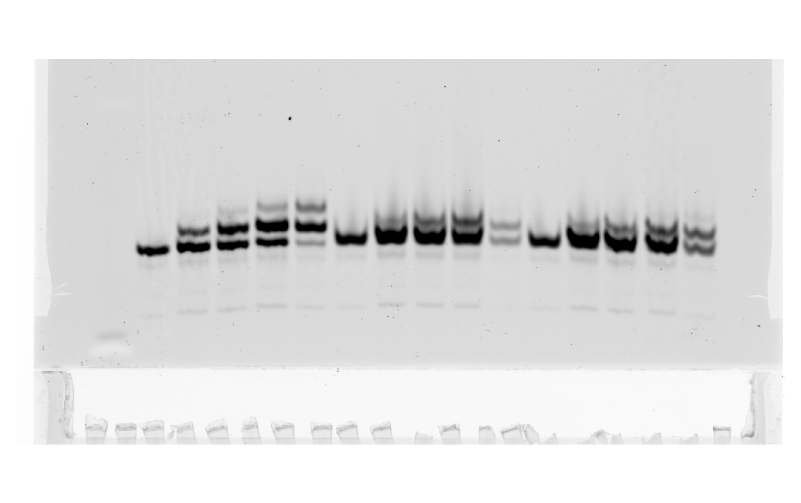 |
|  | C | 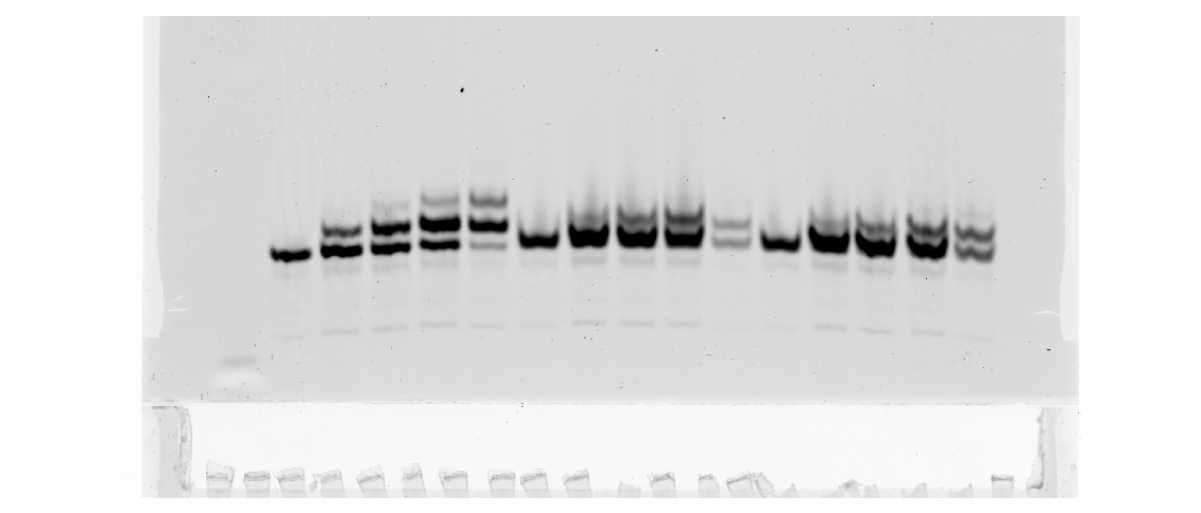 |
|  | D | 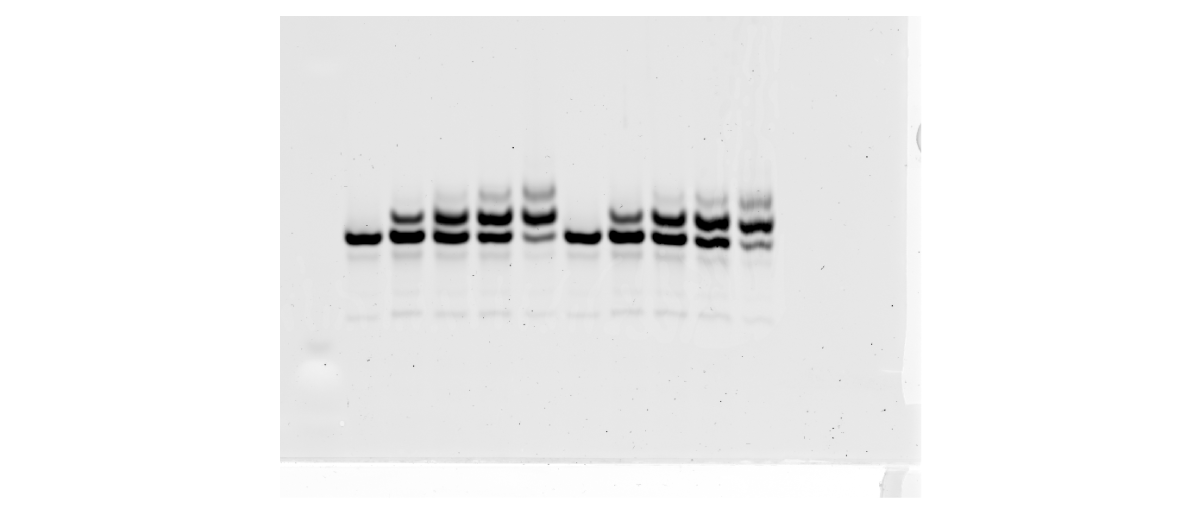 |
| R_6_/Arich_12_  20:10 [Arg]:[nt]^a^ | A | 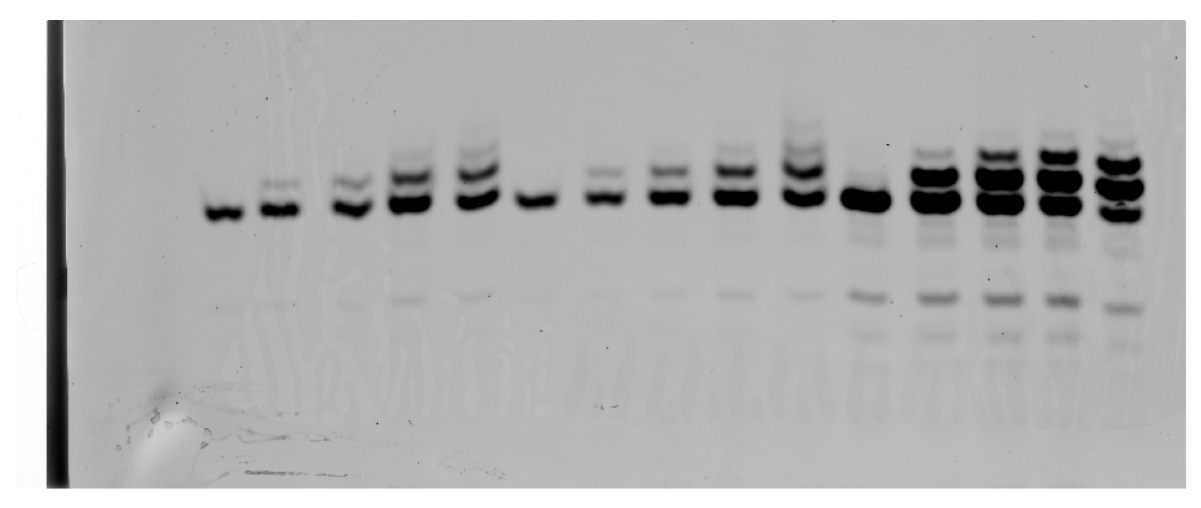 |
|  | B | 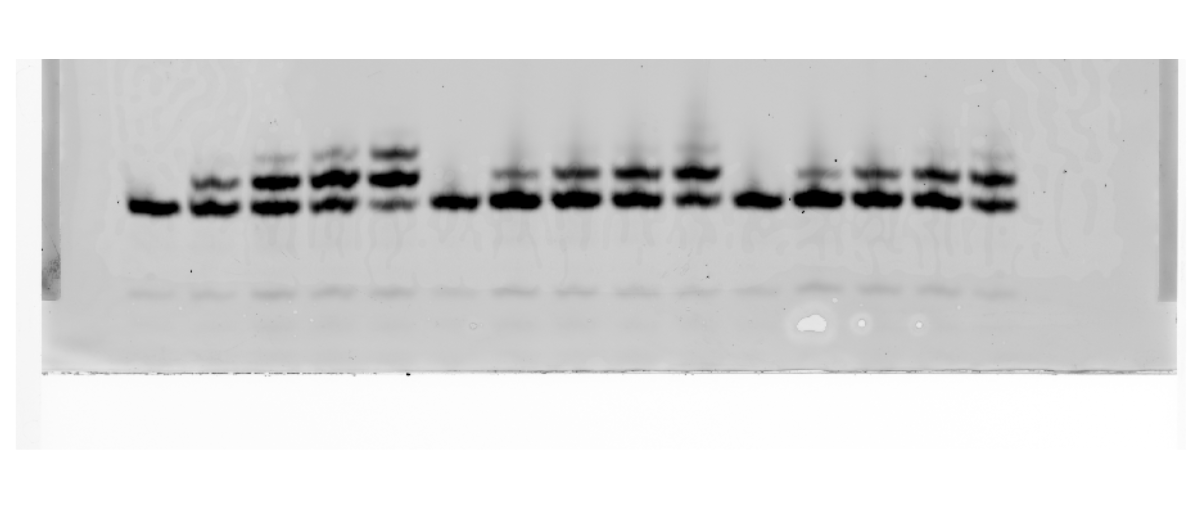 |
|  | C | 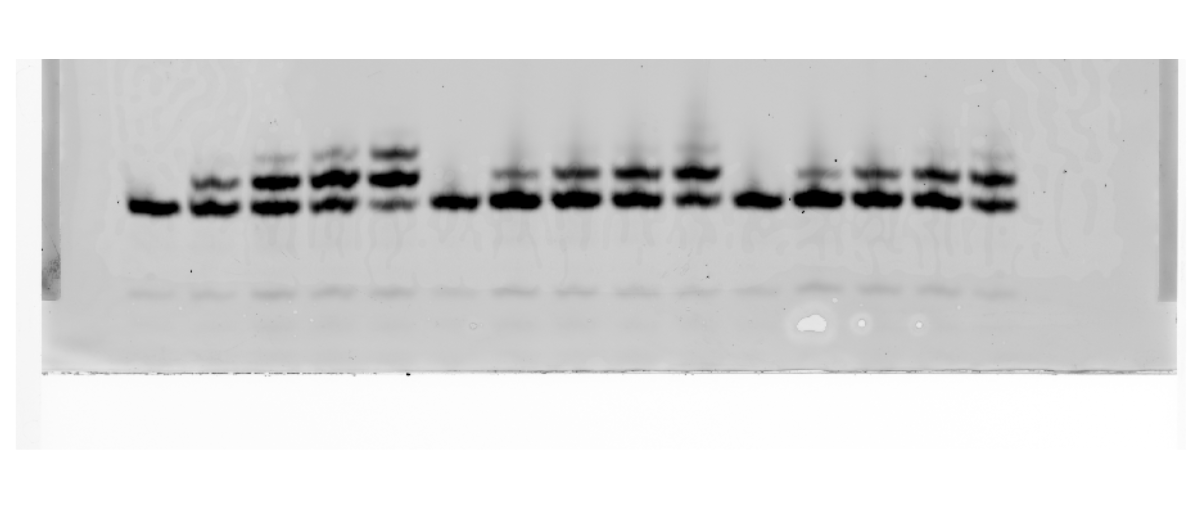 |
|  | D | 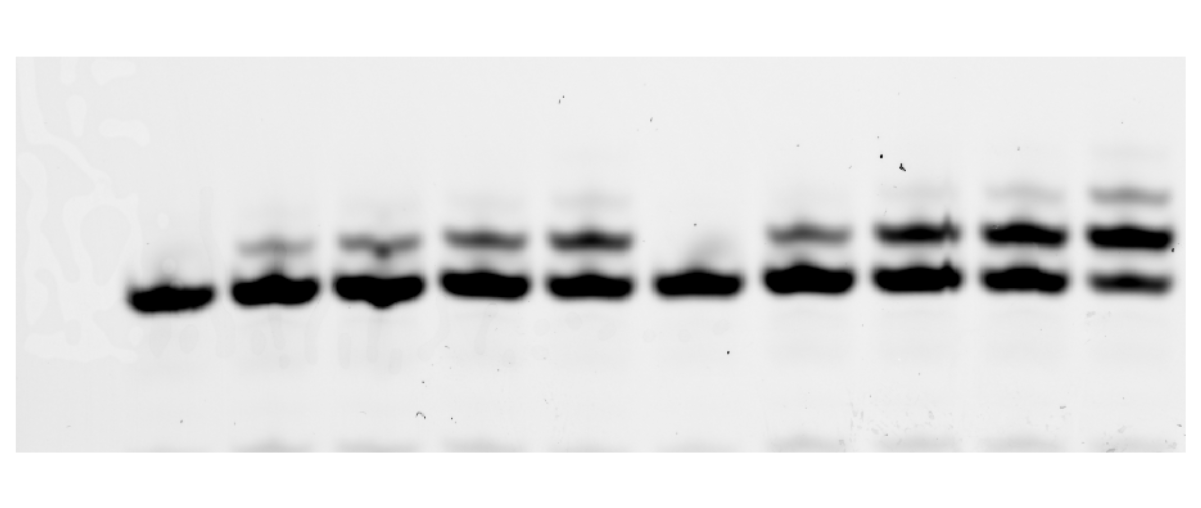 |
| R_6_/Arich_12_  20:10 [Arg]:[nt] | A | 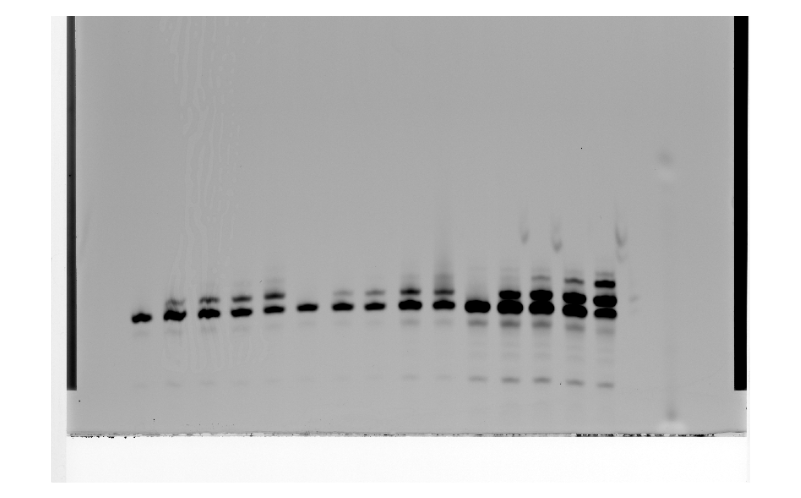 |
|  | B | 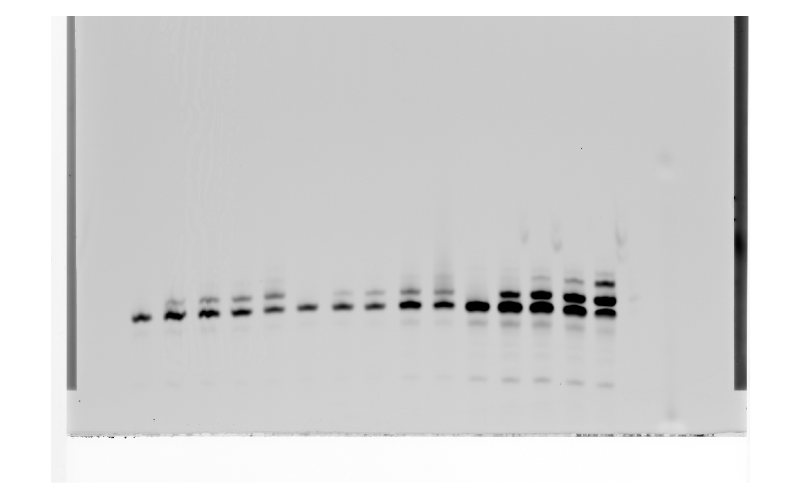 |
|  | C | 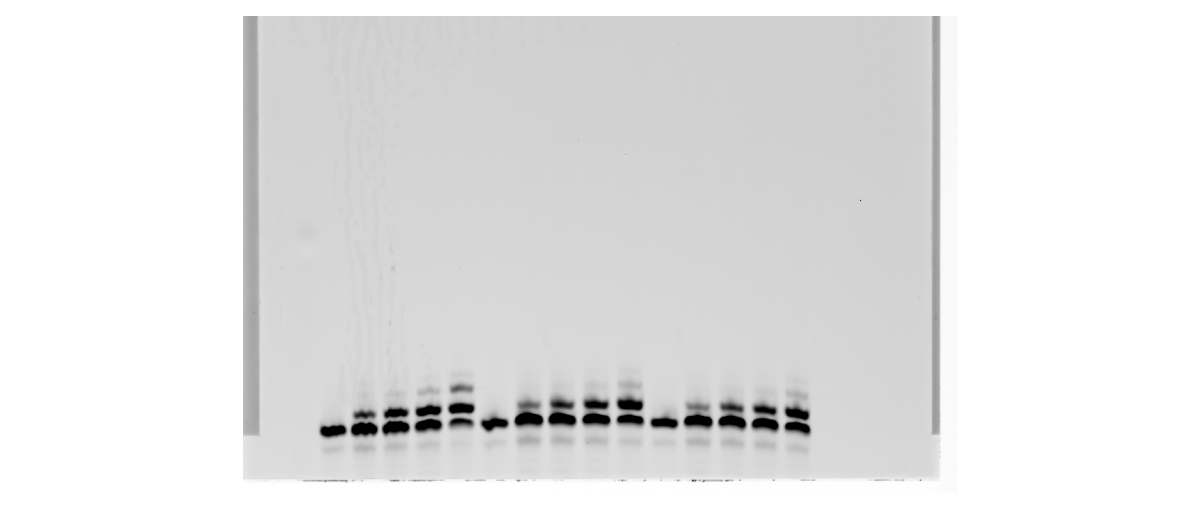 |
|  | D | 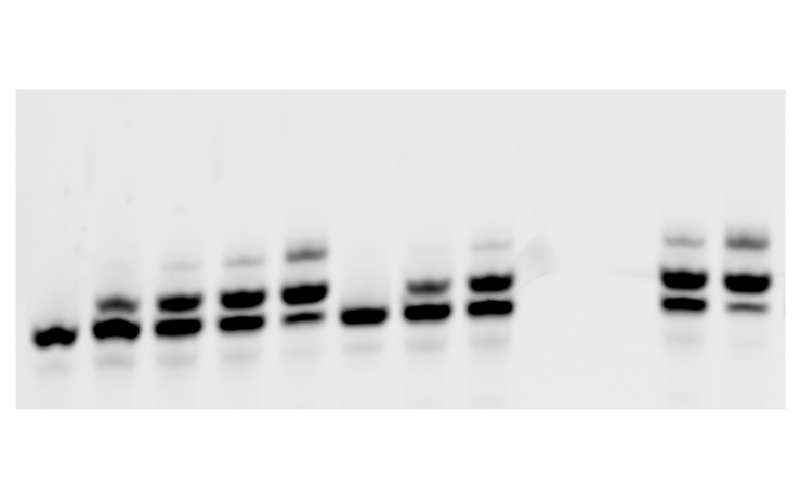 |
| R_6_/Arich_12_  40:5 [Arg]:[nt]^a^ | A | 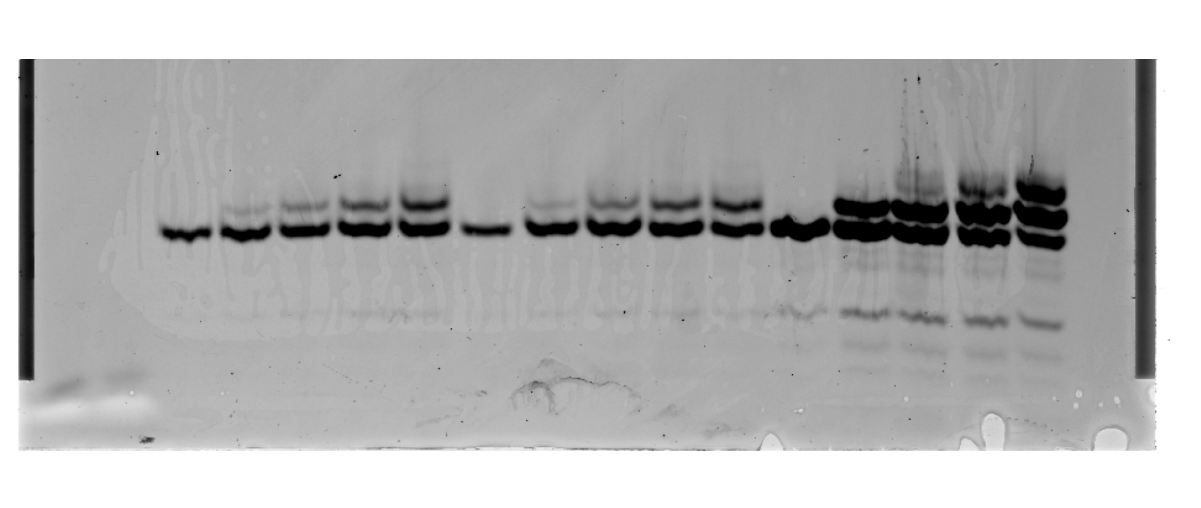 |
|  | B | 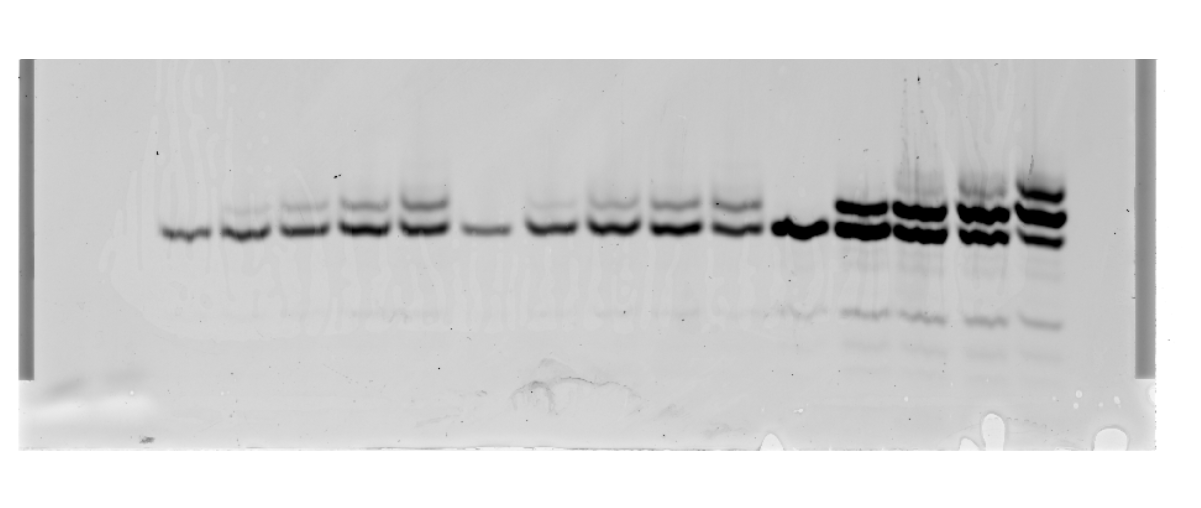 |
|  | C | 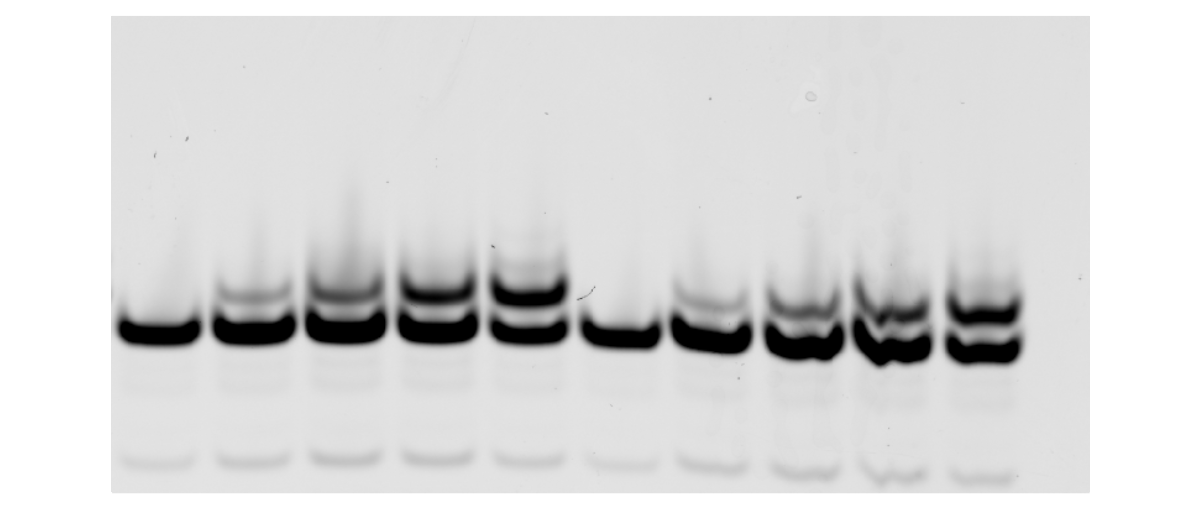 |
|  | D |  |
| Control  (no host)^a^ | B |  |
|  | D |  |
| Control  (no host, w/ R_4_) | A |  |
|  | B |  |
|  | C |  |
|  | D |  |
| Control  (no host, w/ R_6_) | A |  |
|  | C |  |

^a^ reactions performed with a primer/template system that is complementary to the host NA_12_ sequence ((ACTG)_3_) or (ACUG)_3_).

**Table S14.** Average values for primer extension yields as analysed by PAGE (n ≥ 2 replicates). Columns represent different reaction conditions: A = w/ peptide, no NaCl; B = no peptide, no NaCl; C = w/ peptide, w/ NaCl; D = no peptide, w/ NaCl. Relative PE efficiency as plotted in Figures 4d and 4e was calculated using: A/C (“with coacervates”) and B/D (“without coacervates”).

|  | | **Extended primer (%)** | | | |
| --- | --- | --- | --- | --- | --- |
| **Mixture** | **Time** | **A** | **B** | **C** | **D** |
| R_4_/dA_12_  40:5 [Arg]:[nt] | 0 h | 0.0 | 0.0 | 0.0 | 0.0 |
|  | 1 h | 3.7 | 20.9 | 3.1 | 12.6 |
|  | 3 h | 5.6 | 46.5 | 5.0 | 29.7 |
|  | 6 h | 8.1 | 65.5 | 6.7 | 46.2 |
|  | 24 h | 14.5 | 91.3 | 11.2 | 80.8 |
| R_6_/dA_12_  20:10 [Arg]:[nt]^a^ | 0 h | 0.0 | 0.0 | 0.0 | 0.0 |
|  | 1 h | 21.1 | 24.6 | 8.4 | 12.5 |
|  | 3 h | 43.7 | 50.1 | 16.9 | 28.8 |
|  | 6 h | 55.6 | 68.2 | 26.9 | 44.8 |
|  | 24 h | 78.5 | 87.3 | 49.4 | 74.6 |
| R_6_/dA_12_  20:10 [Arg]:[nt] | 0 h | 0.0 | 0.0 | 0.0 | 0.0 |
|  | 1 h | 17.4 | 16.0 | 8.9 | 13.0 |
|  | 3 h | 31.1 | 36.0 | 16.3 | 31.4 |
|  | 6 h | 35.1 | 54.2 | 25.7 | 48.5 |
|  | 24 h | 65.0 | 86.0 | 50.4 | 82.1 |
| R_6_/rA_12_  20:10 [Arg]:[nt]^a^ | 0 h | 0.0 | 0.0 | 0.0 | 0.0 |
|  | 1 h | 15.3 | 35.5 | 16.4 | 18.6 |
|  | 3 h | 32.1 | 64.3 | 31.5 | 40.6 |
|  | 6 h | 42.8 | 77.8 | 46.9 | 58.6 |
|  | 24 h | 58.2 | 90.2 | 73.9 | 83.1 |
| R_6_/rA_12_  20:10 [Arg]:[nt]^a^  + 50 mM NaCl | 0 h | 0.0 |  | | |
|  | 1 h | 13.9 |  |  |  |
|  | 3 h | 24.8 |  |  |  |
|  | 6 h | 32.0 |  |  |  |
|  | 24 h | 47.9 |  |  |  |
| R_6_/mA_12_  20:10 [Arg]:[nt]^a^ | 0 h | 0.0 | 0.0 | 0.0 | 0.0 |
|  | 1 h | 15.5 | 21.6 | 10.4 | 12.2 |
|  | 3 h | 31.8 | 47.0 | 22.7 | 29.0 |
|  | 6 h | 44.2 | 64.6 | 30.8 | 44.8 |
|  | 24 h | 63.6 | 86.6 | 56.9 | 75.3 |
| R_6_/dA_12_  40:5 [Arg]:[nt]^a^ | 0 h | 0.0 | 0.0 | 0.0 | 0.0 |
|  | 1 h | 13.6 | 24.4 | 7.8 | 9.5 |
|  | 3 h | 27.7 | 52.9 | 16.8 | 23.8 |
|  | 6 h | 36.1 | 67.2 | 24.3 | 37.6 |
|  | 24 h | 55.3 | 88.6 | 43.1 | 68.1 |
| R_6_/rA_12_  40:5 [Arg]:[nt]^a^ | 0 h | 0.0 | 0.0 | 0.0 | 0.0 |
|  | 1 h | 12.0 | 22.8 | 12.6 | 13.7 |
|  | 3 h | 22.3 | 49.8 | 20.7 | 33.2 |
|  | 6 h | 26.3 | 67.2 | 31.7 | 48.2 |
|  | 24 h | 36.9 | 88.6 | 49.7 | 53.3 |
| R_6_/mA_12_  40:5 [Arg]:[nt]^a^ | 0 h | 0.0 | 0.0 | 0.0 | 0.0 |
|  | 1 h | 8.3 | 33.7 | 10.3 | 19.8 |
|  | 3 h | 17.5 | 65.0 | 18.9 | 43.2 |
|  | 6 h | 24.2 | 81.8 | 27.4 | 59.8 |
|  | 24 h | 36.0 | 90.4 | 42.1 | 76.5 |
| R_6_/dA_16_  20:10 [Arg]:[nt]^a^ | 0 h | 0.0 | 0.0 | 0.0 | 0.0 |
|  | 1 h | 14.2 | 21.2 | 11.7 | 14.1 |
|  | 3 h | 28.5 | 45.7 | 22.3 | 33.3 |
|  | 6 h | 40.0 | 62.6 | 35.4 | 49.2 |
|  | 24 h | 63.1 | 83.5 | 57.2 | 76.6 |
| R_6_/rA_16_  20:10 [Arg]:[nt]^a^ | 0 h | 0.0 | 0.0 | 0.0 | 0.0 |
|  | 1 h | 10.5 | 19.3 | 7.8 | 15.2 |
|  | 3 h | 15.9 | 42.9 | 14.7 | 33.8 |
|  | 6 h | 22.0 | 60.6 | 22.5 | 49.6 |
|  | 24 h | 31.1 | 87.6 | 38.7 | 78.4 |
| R_4_/dT_16_  20:10 [Arg]:[nt] | 0 h | 0.0 | 0.0 | 0.0 | 0.0 |
|  | 1 h | 6.6 | 18.6 | 6.1 | 14.9 |
|  | 3 h | 13.9 | 41.1 | 12.3 | 34.3 |
|  | 6 h | 22.2 | 60.7 | 19.5 | 52.5 |
|  | 24 h | 42.9 | 90.3 | 40.5 | 85.6 |
| R_6_/dT_16_  20:10 [Arg]:[nt] | 0 h | 0.0 | 0.0 | 0.0 | 0.0 |
|  | 1 h | 11.7 | 17.2 | 8.8 | 14.6 |
|  | 3 h | 19.0 | 38.9 | 20.3 | 33.7 |
|  | 6 h | 27.0 | 58.9 | 31.2 | 51.5 |
|  | 24 h | 42.8 | 90.0 | 62.6 | 85.2 |
| R_4_/DNA_12_  20:10 [Arg]:[nt] | 0 h | 0.0 | 0.0 | 0.0 | 0.0 |
|  | 1 h | 6.3 | 16.5 | 5.8 | 13.7 |
|  | 3 h | 12.4 | 37.2 | 12.0 | 31.6 |
|  | 6 h | 18.6 | 56.2 | 19.2 | 50.8 |
|  | 24 h | 32.1 | 88.1 | 40.9 | 83.5 |
| R_4_/DNA_12_  40:5 [Arg]:[nt]^a^ | 0 h | 0.0 | 0.0 | 0.0 | 0.0 |
|  | 1 h | 5.2 | 9.0 | 3.1 | 8.6 |
|  | 3 h | 5.2 | 20.1 | 4.8 | 19.3 |
|  | 6 h | 5.6 | 32.0 | 5.9 | 30.9 |
|  | 24 h | 7.7 | 55.1 | 11.5 | 55.3 |
| R_4_/DNA_12_  40:5 [Arg]:[nt] | 0 h | 0.0 | 0.0 | 0.0 | 0.0 |
|  | 1 h | 3.3 | 14.6 | 4.7 | 13.2 |
|  | 3 h | 6.0 | 32.9 | 7.0 | 29.9 |
|  | 6 h | 8.5 | 52.7 | 10.4 | 45.9 |
|  | 24 h | 13.8 | 81.7 | 19.3 | 79.4 |
| R_6_/DNA_12_  20:10 [Arg]:[nt]^a^ | 0 h | 0.0 | 0.0 | 0.0 | 0.0 |
|  | 1 h | 6.3 | 7.0 | 7.5 | 7.9 |
|  | 3 h | 9.8 | 16.5 | 14.2 | 17.4 |
|  | 6 h | 10.9 | 26.8 | 21.0 | 28.8 |
|  | 24 h | 14.2 | 49.6 | 37.6 | 51.2 |
| R_6_/DNA_12_  20:10 [Arg]:[nt] | 0 h | 0.0 | 0.0 | 0.0 | 0.0 |
|  | 1 h | 16.3 | 16.7 | 12.4 | 14.3 |
|  | 3 h | 28.4 | 38.1 | 21.6 | 33.6 |
|  | 6 h | 35.5 | 56.3 | 34.6 | 51.0 |
|  | 24 h | 41.7 | 87.8 | 61.6 | 78.2 |
| R_6_/DNA_12_  40:5 [Arg]:[nt] | 0 h | 0.0 | 0.0 | 0.0 | 0.0 |
|  | 1 h | 10.1 | 15.4 | 5.6 | 16.1 |
|  | 3 h | 14.2 | 35.7 | 7.8 | 35.3 |
|  | 6 h | 15.7 | 53.1 | 10.0 | 51.5 |
|  | 24 h | 21.2 | 84.9 | 16.7 | 78.4 |
| R_4_/RNA_12_  40:5 [Arg]:[nt]^a^ | 0 h | 0.0 | 0.0 | 0.0 | 0.0 |
|  | 1 h | 0.0 | 0.0 | 0.0 | 0.0 |
|  | 3 h | 0.0 | 0.0 | 0.0 | 0.0 |
|  | 6 h | 0.0 | 0.0 | 0.0 | 0.0 |
|  | 24 h | 0.0 | 0.0 | 0.0 | 0.0 |
| R_4_/RNA_12_  40:5 [Arg]:[nt] | 0 h | 0.0 | 0.0 | 0.0 | 0.0 |
|  | 1 h | 7.0 | 17.0 | 5.7 | 11.2 |
|  | 3 h | 8.4 | 36.3 | 6.7 | 25.7 |
|  | 6 h | 11.9 | 52.1 | 10.0 | 39.8 |
|  | 24 h | 16.2 | 82.9 | 17.6 | 74.3 |
| R_6_/RNA_12_  20:10 [Arg]:[nt] | 0 h | 0.0 | 0.0 | 0.0 | 0.0 |
|  | 1 h | 17.5 | 18.8 | 15.4 | 15.3 |
|  | 3 h | 19.6 | 40.3 | 31.1 | 33.9 |
|  | 6 h | 23.5 | 59.0 | 43.8 | 52.7 |
|  | 24 h | 27.1 | 88.0 | 68.3 | 83.1 |
| R_6_/RNA_12_  40:5 [Arg]:[nt] | 0 h | 0.0 | 0.0 | 0.0 | 0.0 |
|  | 1 h | 10.2 | 21.7 | 11.3 | 15.6 |
|  | 3 h | 12.4 | 47.5 | 21.2 | 34.9 |
|  | 6 h | 14.4 | 67.5 | 32.4 | 54.9 |
|  | 24 h | 23.0 | 82.9 | 52.0 | 84.6 |
| R_6_/Arich_12_  20:10 [Arg]:[nt]^a^ | 0 h | 0.0 | 0.0 | 0.0 | 0.0 |
|  | 1 h | 8.6 | 26.9 | 10.3 | 25.5 |
|  | 3 h | 16.3 | 52.9 | 21.3 | 55.4 |
|  | 6 h | 23.3 | 69.4 | 31.5 | 72.2 |
|  | 24 h | 36.3 | 85.5 | 56.0 | 93.4 |
| R_6_/Arich_12_  20:10 [Arg]:[nt] | 0 h | 0.0 | 0.0 | 0.0 | 0.0 |
|  | 1 h | 8.8 | 11.7 | 6.8 | 15.2 |
|  | 3 h | 15.5 | 27.7 | 13.9 | 34.8 |
|  | 6 h | 18.4 | 44.6 | 22.2 | 52.3 |
|  | 24 h | 32.1 | 78.8 | 46.5 | 85.1 |
| R_6_/Arich_12_  40:5 [Arg]:[nt]^a^ | 0 h | 0.0 | 0.0 | 0.0 | 0.0 |
|  | 1 h | 8.3 | 25.1 | 7.0 | 12.1 |
|  | 3 h | 18.2 | 50.8 | 15.5 | 29.1 |
|  | 6 h | 23.1 | 67.4 | 24.6 | 44.3 |
|  | 24 h | 35.6 | 85.7 | 42.5 | 72.1 |
| Control  (no host)^a^ | 0 h |  | 0.0 |  | 0.0 |
|  | 1 h |  | 20.9 |  | 11.1 |
|  | 3 h |  | 45.6 |  | 27.9 |
|  | 6 h |  | 61.0 |  | 42.5 |
|  | 24 h |  | 80.9 |  | 68.1 |
| Control  (no host, w/ R_4_) | 0 h | 0.0 | 0.0 | 0.0 | 0.0 |
|  | 1 h | 7.4 | 18.8 | 3.9 | 11.0 |
|  | 3 h | 10.6 | 41.1 | 9.2 | 25.1 |
|  | 6 h | 15.6 | 58.5 | 13.0 | 39.4 |
|  | 24 h | 30.3 | 89.7 | 23.5 | 75.3 |
| Control  (no host, w/ R_6_) | 0 h | 0.0 | 0.0 | 0.0 | 0.0 |
|  | 1 h | 38.8 | 18.8 | 12.2 | 11.0 |
|  | 3 h | 63.4 | 41.1 | 22.6 | 25.1 |
|  | 6 h | 86.2 | 58.5 | 34.5 | 39.4 |
|  | 24 h | 96.3 | 89.7 | 64.1 | 75.3 |

^a^ reactions performed with a primer/template system that is complementary to the host RNA_12_ sequence ((ACUG)_3_).

# Supplementary Figures

**Fig. S1.** ¹H-NMR (500 MHz, D₂O:H_2_O 9:1) spectrum of **R_4_** (top) and **R_2_** (bottom) synthesised in-house.

**Fig. S2.** **(a)** Salt titration curves used to determine the CSC values **(b)** for R_4_/DNA_20_ and R_8_/DNA_20_ at 20 mM arginine and 5 mM nucleotide concentrations.

**Fig. S3. (a)** Salt titration curves of R_4_/DNA_8_ mixtures at 5 mM nucleotide. These curves are used to determine **(b)** the phase diagram of the mixture. CSC values were measured at different amino acid concentrations.

**Fig. S4.** **(a)** Salt titration curves of R_4_/DNA_8_ mixtures at 20 mM amino acid. These curves are used to determine **(b)** the phase diagram of the mixture. The CSC values were measured at different nucleotide concentrations.

**Fig. S5.** Phase diagram of the mixtures in **Figure 1** (main text), obtained by varying the concentration of the anionic monomer: nucleotide in the case of peptide/oligonucleotide mixtures **(a)**; glutamic acid in the case of the peptide/peptide coacervates **(b)**.

**Fig. S6.** Bright-field images of minimal coacervates in 25 mM HEPES pH 7.4, 25 mM imidazole pH 7.5 or 25 mM phosphate buffer pH 7.5. Scale bar = 10 μm.

**Fig. S7.** Minimal coacervates in 25 mM imidazole buffer at different pH values, observed by bright-field microscopy. Scale bar = 10 μm.

**Fig. S8. (a)** Extended dataset for the thermal stability of peptide/oligonucleotide mixtures as in **Figure 1** (main text). The R_4_/dsDNA_8_ mixture is added for comparison and shows an additional phase transition before room temperature. R_4_/DNA_16_, despite its similar CSC to R_4_/RNA_8_, did not fully dissolve in the heating ramp. All mixtures reassemble into droplets upon cooling, and coacervate fluorescence is recovered. Fluorophore: Cy3-(TGAC)_2_. (b) Thermal stability of R_10_/E_10_ coacervates, as prepared for the comparison in Figure 1, with the addition of 1% R_8_-FITC. Scale bar: 10 µm.

**Fig. S9.** Coacervates composed of R_2_ and oligonucleotides (8-20 nt). Required concentrations of the components are listed in Table S5. Scale bar = 10 μm, bright-field microscopy.

**Fig. S10.** CSC dependence on the inverse of the length of different peptides and oligonucleotides enabling the prediction of the shortest peptide to form coacervates with DNA_12_ or RNA_12_ (motif ACTG, open circles; or ACUG, purple triangles). The details of the linear regression are shown to calculate the length for CSC > 0.

**Fig. S11.** CSC dependence on the inverse of the length of different peptides and oligonucleotides enabling the prediction of the shortest peptide to form coacervates with a mixture of DNA_12_ and RNA_12_ (purple diamonds) or with the hybrid strand HNA_12_ (magenta circles). The linear fits overlap, and details are shown in **Table S5.** Amino acid concentrations required for the coacervation of dipeptides with oligonucleotides of different lengths. *N/A* stands for ‘non-applicable’.

| **Peptide dimer** | **Oligonucleotide** | **Phase** | **Amino acid concentration required** |
| --- | --- | --- | --- |
| **R2** | DNA8 | Soluble | *N/A* |
|  | DNA12 | Soluble | *N/A* |
|  | DNA20 | Soluble | *N/A* |
|  | RNA8 | Soluble | *N/A* |
|  | RNA12 | **Droplets** | 60 mM |
|  | RNA20 | **Droplets** | 40 mM |

**Table S66**. The details of the linear regression are shown to calculate the length for CSC > 0.

**Fig. S12.** **(a)** Mixtures of R_10_/dX_10_ imaged by epifluorescence microscopy (probe: Cy3-10nt). Decamers of C and G, the bases capable of three hydrogen bonds, lead to solid aggregation instead of liquid droplets. **(b)** Critical salt concentrations (CSCs) of R_10_/X_10_ mixtures that form coacervates, measured at 20 mM amino acid and 5 mM nucleotide, without any labelled oligo added. The CSC of solid aggregates is not a defined property.

**Fig. S13.** CSC dependence on the inverse of the length of different peptides and oligonucleotides enabling the prediction of the shortest homopolymeric DNA (polyA_N_, diamonds) to form coacervates with peptide R_6_, in comparison to when a heteropolymeric DNA sequence is used (inverted triangles). The details of the linear regression are shown to calculate the length for CSC > 0.

**Fig. S14.** R_4_ -based coacervates made with phosphate-modified oligos. Scale bar = 10 μm, bright-field microscopy.

**Fig. S15.** Number of contacts per nucleotide through all three interaction modes for the four systems simulated atomistically. Error bars represent the standard deviation across 5 repeats. Individual values corresponding to each repeat can be found in Table S7.

**Fig. S16.** Number of contacts *via* H-bonding, per nucleotide, established through the phosphate, sugar and base moieties. Error bars represent the standard deviation across 5 repeats. Individual values corresponding to each repeat can be found in Table S8.

**Fig. S17.** Number of contacts established per oligonucleotide, grouped into three types of contact: with any peptide moiety, with unique arginine residues, or with unique peptide chains. Error bars represent the standard deviation across 5 repeats. The number of contacts with any peptide moiety is the “total valency” represented in **Figure 2** (main text). Individual values corresponding to each repeat can be found in Table S9.

**Fig. S18.** Unique oligonucleotide chains contacted by a peptide chain for the different mixtures simulated atomistically. The mixture R_3_/DNA_8_ is the only one with a median <1, *i.e.*, on average, the peptide R_3_ is free in the presence of DNA_8_ chains. In this case, we represent each peptide chain sampled (36 chains, 5 repeats) instead of an average. Boxplots contain 50% of the data points measured for the 36 peptide chains in the simulation, averaged over time per simulation repeat (5x). The horizontal line represents the median. Individual values corresponding to each repeat can be found in Table S10.

**Fig. S19.** Cy_3_-DNA_5_ in R_4_/DNA_20_ coacervates, showing that a DNA pentamer is recruited in the droplets. Scale bar = 10 μm, epifluorescence microscopy.

**Fig. S20.** FRAP profiles of a series of coacervates where peptide and DNA length were varied. Non-complementary probes of three lengths were tested for each coacervate system: 8, 16 and 32nt.

**Fig. S21.** Expanded dataset of FRAP recovery times for peptide-, DNA- and probe-length series as shown in **Figure 3** (main text).

**Fig. S22.** FRAP profiles of coacervates showcasing the effect of the nature of the scaffold. **(a)** and **(b)** refer to the effect of homopolymers and heteropolymers of DNA. **(c)** probes the effect of DNA versus RNA as scaffold strands. **(d)** shows the effect of the scaffold polymer length (both peptide and DNA). **(e)** compares diffusion in DNA 12mers of different sequences. **(f)** shows the effect of having dsDNA as scaffold strands.

**Fig. S23. FRAP profiles of RNA- and DNA-based coacervates in the presence of R_6_**. **(a)** shows the effect of having salt (50mM NaCl) in solution, which partially dissolves the coacervates and seemingly fluidizes the system. **(b)** compares. RNA-based coacervates with coacervates made of mixtures of RNA and DNA of the same length (rA_12_, dA_12_) or different lengths (rA_12_, dA_3_) in equimolar quantities. The presence of DNA actively fluidizes the coacervates as they are more fluid than those made up of half concentration of RNA only (rA_12_ 5mM). **(c)** shows the length component in DNA polyA systems (dA_12_ and dA_16_, respectively).

**Fig. S24.** Confocal micrographs of aptamer reconstitution in coacervates. Scale bars are 10 µm. **(a)** Confocal fluorescence micrographs of different controls and test samples under constant irradiation conditions in the 488 nm channel (DFHBI). The DIC channel is only shown for (1) and (2) to confirm the presence of coacervates. **(b)** Quantification of the total DFHBI emission in the field of view shown in (a). Error bars come from measurements in triplicate. **(c)** DFHBI emission in the presence of coacervates, now separating emission from all droplets in the FOV and the background (dilute phase). Samples were prepared adding DFHBI as the last component.

**Fig. S25.** DFHBI/Broccoli aptamer emission measured by confocal microscopy and fluorescence spectroscopy (bulk). Expanded dataset shown in **Figure 4** (main text), including emission measured before and immediately after coacervation. Samples were prepared by adding the peptide as the last component, *i.e.*, coacervation occurs in the presence of the reconstituted aptamer.

**Fig. S26.** Stability of peptide/nucleic acid coacervates during primer extension. Micrographs of reaction mixtures for primer extension by transmission and epifluorescence (FAM-labelled primer). The coacervate scaffold is composed of: **(a)** 40 mM (aa) R_4_, 5 mM (nt) (ACTG)_3_, **(b)** 40 mM R_4_, 5 mM (ACUG)_3_. **(a)** and **(b)** contain 1% of the FAM-labelled primer strand and 1% of the template strand. (a-c) contain Mg^2+^ 5 mM, activated dimer 5.0 mM and 25 mM HEPES pH 7.4.

**a**

**b**

**Fig. S27.** Primer extension efficiencies over time in the presence or absence of bystander (host) oligonucleotides. PE was tested with a primer/template system with no complementarity with the host oligonucleotide **(a)**, and a primer/template system with complementarity with the host NA_12_ oligonucleotide **(b)**. Data were fit to first order exponential. Abbreviations: PE = primer extension, dA_12_ = 12-deoxyribonucleotide-long polyadenine oligonucleotide, rA_12_ = 12-ribonucleotide-long polyadenine oligonucleotide, mA_12_ = dA_12_:rA_12_ 1:1 ratio, Arich_12_ = 12-deoxyribonucleotide-long A-rich-sequence oligonucleotide, DNA_12_ = 12-deoxyribonucleotide-long mixed-sequence oligonucleotide ((ACTG)_3_).

**a**

**b**

**Fig. S28.** **(a)** Degradation profile of the activated imidazolium-bridged dicytidyl dimer in the absence (grey filled dots) and in the presence of glycine (red filled dots). **(b)** Representative NMR spectra after 24 hours, highlighting degradation products (e.g., cytidine monophosphate and activated monomer), are shown for the activated dimer with and without glycine.

**Fig. S29.** Primer extension efficiencies over time in the presence of dA_12_ and R_6_ (20:10 [Arg]:[nt] ratio) without NaCl to enable coacervation (filled circles) or with NaCl to prevent coacervation (empty circles). PE efficiencies were normalised against their respective control reactions. Data were fit to first order exponential.

**Fig. S30.** Primer extension efficiencies over time in the presence of dA_12_ and R_6_ (40:5 [Arg]:[nt] ratio) without NaCl to enable coacervation (filled circles) or with NaCl to prevent coacervation (empty circles). PE efficiencies were normalised against their respective control reactions. Data were fit to first order exponential.

**Fig. S31.** Primer extension efficiencies over time in the presence of dA_12_ and R_6_ (20:10 [Arg]:[nt] ratio) with two different primer/template (P/T) pairs. PE efficiencies were normalised against their respective control reactions. Data were fit to first order exponential.

**Fig. S32.** Coarse-grained simulation of U_12_ system. **(a)** Density profile and simulation snapshot for the 3 R4 : 1 U12 system, showing an approximately uniform density throughout the box, indicating that liquid-liquid phase separation (LLPS) does not occur for this mixture. **(b)** Density profile and simulation snapshot for the 2 R6 : 1 U12 system, showing a pronounced peak in the density of RNA and peptide at the center of the box, corresponding to the formation of a condensate. **(c)** Histogram showing differences in valency in the charge-balanced 3 R4 : 1 U12 and 2 R6 : 1 U12 systems. The R6 system shows many peptides simultaneously contacting 3,4, or 5 unique RNA chains, whilst in the R4 system very few peptides contact more than 2 RNA chains simultaneously. **(d)** Density profile and simulation snapshot for the 3 R6 : 1 U12 system, illustrating the effects of oversaturating with peptide. While the peptide:RNA ratio increases only moderately (~19%) in the central dense region, a much larger (~140%) increase is observed in the dilute phase.

**a**

**b**

**Fig. S33.** **(a)** Primer extension efficiencies over time in the presence of dA_12_ and R_6_ at different charge ratios between peptide and oligonucleotide. **(b)** Primer extension efficiencies over time in the presence of DNA_12_ and R_6_ at different charge ratios between peptide and oligonucleotide. A primer/template system that is non-complementary to the host strand was employed in this study. PE efficiencies were normalised against their respective control reactions. Data were fit to first order exponential.

**Fig. S34.** Primer extension efficiencies over time in the presence of R_6_ (20:10 [Arg]:[nt] ratio) and dA_12_, dA_16_, rA_12_ and rA_16_. PE efficiencies were normalised against their respective control reactions. Data were fit to first order exponential.

**Fig. S35.** Primer extension efficiencies over time in the presence of R_6_ (20:10 [Arg]:[nt] ratio) and dA_12_, mA_12_ (dA_12_:rA_12_ 1:1) and rA_12_. PE efficiencies were normalised against their respective control reactions. Data were fit to first order exponential.

**Fig. S36.** Primer extension efficiencies over time in the presence of DNA_12_ (40:5 [Arg]:[nt] ratio) and R_6_ or R_4_. A primer/template system that is non-complementary to the host strand was employed in this study. PE efficiencies were normalised against their respective control reactions. Data were fit to first order exponential.

**Fig. S37.** Primer extension efficiencies over time in the presence of R_6_ (20:10 [Arg]:[nt] ratio) and dA_12_, Arich_12_ and DNA_12_. PE efficiencies were normalised against their respective control reactions. Data were fit to first order exponential.

**Fig. S38.** Primer extension efficiencies over time in the presence of DNA_12_ and R_6_ with primer/template systems that are complementary or non-complementary to the host strand. PE efficiencies were normalised against their respective control reactions. Data were fit to first order exponential.
