# Supplementary material for: Differential stability and dynamics of DNA-based and RNA-based coacervates affect non-enzymatic RNA chemistry
Source: Nat Commun. 2025 Oct 21;16:9296. doi: 10.1038/s41467-025-64335-9 (PMC12540774; doi:10.1038/s41467-025-64335-9)
Supplement: Supplementary file 2 — Reporting Summary [file 41467_2025_64335_MOESM2_ESM.pdf]

nature portfolio

Corresponding author(s): Rosana Collepardo-Guevara, Derek O'Flaherty, Claudia Bonfio

Last updated by author(s): Sep 1, 2025

# Reporting Summary

Nature Portfolio wishes to improve the reproducibility of the work that we publish. This form provides structure for consistency and transparency in reporting. For further information on Nature Portfolio policies, see our Editorial Policies and the Editorial Policy Checklist.

## Statistics

For all statistical analyses, confirm that the following items are present in the figure legend, table legend, main text, or Methods section.

| n/a | Confirmed | |
|---|---|---|
| ☐ | ☒ | The exact sample size (*n*) for each experimental group/condition, given as a discrete number and unit of measurement |
| ☐ | ☒ | A statement on whether measurements were taken from distinct samples or whether the same sample was measured repeatedly |
| ☐ | ☒ | The statistical test(s) used AND whether they are one- or two-sided<br>*Only common tests should be described solely by name; describe more complex techniques in the Methods section.* |
| ☒ | ☐ | A description of all covariates tested |
| ☒ | ☐ | A description of any assumptions or corrections, such as tests of normality and adjustment for multiple comparisons |
| ☐ | ☒ | A full description of the statistical parameters including central tendency (e.g. means) or other basic estimates (e.g. regression coefficient) AND variation (e.g. standard deviation) or associated estimates of uncertainty (e.g. confidence intervals) |
| ☐ | ☒ | For null hypothesis testing, the test statistic (e.g. *F*, *t*, *r*) with confidence intervals, effect sizes, degrees of freedom and *P* value noted<br>*Give P values as exact values whenever suitable.* |
| ☒ | ☐ | For Bayesian analysis, information on the choice of priors and Markov chain Monte Carlo settings |
| ☒ | ☐ | For hierarchical and complex designs, identification of the appropriate level for tests and full reporting of outcomes |
| ☒ | ☐ | Estimates of effect sizes (e.g. Cohen's *d*, Pearson's *r*), indicating how they were calculated |

*Our web collection on statistics for biologists contains articles on many of the points above.*

## Software and code

Policy information about availability of computer code

**Data collection**

No code or software was involved in collecting experimental data.

All molecular dynamics simulations were performed using OpenMM 8.1.2, with PyMOL 2.5.7 and Packmol 20.14.4 used for atomistic structure preparation, along with OpenMM's Modeller feature for solvating and ionising structures. Coarse-grained simulations utilised the OpenMpipi package available at https://github.com/CollepardoLab/OpenMpipi. The Bede high-performance computing cluster was used to run all simulations with access provided through the HecBioSim consortium. Example scripts for all simulations are available at https://github.com/CollepardoLab/MinimalCoacervates.

**Data analysis**

Analysis of experimental data was done using commercial software: FRAP data was processed using a custom made MatLab script available upon request. All data fitting was performed using Graphpad Prism 10 and OriginPro 2021, as made available by the University of Cambridge. Image processing was done with the open-source software ImageJ 1.52p. All PAGE analyses were conducted with the standard ImageQuant(TM) software.

Analysis of atomistic and coarse-grained simulation trajectories was carried out using custom, in-house Python scripts using the CuPy and MDTraj Python packages. Analysis scripts are available at https://github.com/CollepardoLab/MinimalCoacervates.

For manuscripts utilizing custom algorithms or software that are central to the research but not yet described in published literature, software must be made available to editors and reviewers. We strongly encourage code deposition in a community repository (e.g. GitHub). See the Nature Portfolio guidelines for submitting code & software for further information.

## Data

Policy information about availability of data

All manuscripts must include a data availability statement. This statement should provide the following information, where applicable:

- Accession codes, unique identifiers, or web links for publicly available datasets
- A description of any restrictions on data availability
- For clinical datasets or third party data, please ensure that the statement adheres to our policy

All data needed to reproduce our work is available in the manuscript, supporting information tables and deposited in the repository: https://doi.org/10.17863/CAM.120272

Example scripts for all simulations and computational data analysis are available at https://github.com/orgs/CollepardoLab/MinimalCoacervates.

## Research involving human participants, their data, or biological material

Policy information about studies with human participants or human data. See also policy information about sex, gender (identity/presentation), and sexual orientation and race, ethnicity and racism.

| | |
|---|---|
| Reporting on sex and gender | - |
| Reporting on race, ethnicity, or other socially relevant groupings | - |
| Population characteristics | - |
| Recruitment | - |
| Ethics oversight | - |

Note that full information on the approval of the study protocol must also be provided in the manuscript.

# Field-specific reporting

Please select the one below that is the best fit for your research. If you are not sure, read the appropriate sections before making your selection.

☒ Life sciences ☐ Behavioural & social sciences ☐ Ecological, evolutionary & environmental sciences

For a reference copy of the document with all sections, see nature.com/documents/nr-reporting-summary-flat.pdf

# Life sciences study design

All studies must disclose on these points even when the disclosure is negative.

| | |
|---|---|
| Sample size | For partition coefficient determination, sample size was determined by the number of droplets in a field of view of the microscope (all droplets were analysed). For FRAP data, sample size is the amount of droplets irradiated across replicates.<br><br>Atomistic simulations were ran with 5 independently prepared replicates for each system, with 800 ns of simulation for each replica totaling 4 microseconds of sampling per system. Coarse-grained density profiles where collected from a single replica over 500 m timesteps, with a 500 m timestep equilibration phase preceding data collection. |
| Data exclusions | No data was excluded from the analyses. |
| Replication | Experiments were reproduced three times (i.e. three different samples) and average values are plotted in the manuscript. FRAP experiments were performed for at least 3 different droplets in a sample, and 2-3 samples. All primer extension experiments were conducted in duplicates.<br><br>Simulations were repeated five times, and measurements for each replicate are available in supporting information tables.<br><br>All replication attempts were successful. |
| Randomization | n/a |
| Blinding | For structure preparation of atomistic simulations, different monomer structures were taken and different random seeds for Packmol packing and OpenMM solvent/ion addition were used for each repeat. Additionally, different seeds where used for the Langevin integrator and Monte Carlo Barostat in each independent simulation run. |

# Reporting for specific materials, systems and methods

We require information from authors about some types of materials, experimental systems and methods used in many studies. Here, indicate whether each material, system or method listed is relevant to your study. If you are not sure if a list item applies to your research, read the appropriate section before selecting a response.

## Materials & experimental systems

| n/a | Involved in the study |
| --- | --- |
| ☒ | ☐ Antibodies |
| ☒ | ☐ Eukaryotic cell lines |
| ☒ | ☐ Palaeontology and archaeology |
| ☒ | ☐ Animals and other organisms |
| ☒ | ☐ Clinical data |
| ☒ | ☐ Dual use research of concern |
| ☒ | ☐ Plants |

## Methods

| n/a | Involved in the study |
| --- | --- |
| ☒ | ☐ ChIP-seq |
| ☒ | ☐ Flow cytometry |
| ☒ | ☐ MRI-based neuroimaging |

## Plants

| | |
| --- | --- |
| Seed stocks | - |
| Novel plant genotypes | - |
| Authentication | - |
